# Beta‐binomial model for meta‐analysis of odds ratios

**DOI:** 10.1002/sim.7233

**Published:** 2017-01-25

**Authors:** Ilyas Bakbergenuly, Elena Kulinskaya

**Affiliations:** ^1^ School of Computing SciencesUniversity of East AngliaNorwichU.K.

**Keywords:** Intra‐cluster correlation, odds ratio, fixed‐effect model, random‐effects model, beta‐binomial distribution, overdispersion, heterogeneity

## Abstract

In meta‐analysis of odds ratios (OR*s*), heterogeneity between the studies is usually modelled via the additive random effects model (REM). An alternative, multiplicative REM for OR*s* uses overdispersion. The multiplicative factor in this overdispersion model (ODM) can be interpreted as an intra‐class correlation (ICC) parameter. This model naturally arises when the probabilities of an event in one or both arms of a comparative study are themselves beta‐distributed, resulting in beta‐binomial distributions. We propose two new estimators of the ICC for meta‐analysis in this setting. One is based on the inverted Breslow‐Day test, and the other on the improved gamma approximation by Kulinskaya and Dollinger (2015, p. 26) to the distribution of Cochran's *Q*. The performance of these and several other estimators of ICC on bias and coverage is studied by simulation. Additionally, the Mantel‐Haenszel approach to estimation of ORs is extended to the beta‐binomial model, and we study performance of various ICC estimators when used in the Mantel‐Haenszel or the inverse‐variance method to combine ORs in meta‐analysis. The results of the simulations show that the improved gamma‐based estimator of ICC is superior for small sample sizes, and the Breslow‐Day‐based estimator is the best for n⩾100. The Mantel‐Haenszel‐based estimator of OR is very biased and is not recommended. The inverse‐variance approach is also somewhat biased for OR*s*≠1, but this bias is not very large in practical settings. Developed methods and R programs, provided in the Web Appendix, make the beta‐binomial model a feasible alternative to the standard REM for meta‐analysis of ORs. © 2017 The Authors. *Statistics in Medicine* Published by John Wiley & Sons Ltd.

## 1 Introduction

Meta‐analysis aims to combine effects estimated from a number of studies to achieve greater precision of the conclusions. The great majority of meta‐analyses use the odds ratio (OR) or its logarithm (LOR) as the effect measure of interest. The OR arises as an effect of interest when the aim is to compare binary outcomes in the treatment and control groups of *K* studies, or to quantify the relation between disease and exposure. Standard models of meta‐analysis are the fixed effect model (FEM) and the random effects model (REM). The former assumes that the LORs *θ*_*j*_,*j*=1,⋯,*K*, do not differ across the studies, that is, *θ*_*j*_≡*θ*; the latter assumes that the LORs themselves are a random sample from, usually, a normal distribution, *θ*_*j*_∼*N*(*θ*,*τ* ^2^) with the between‐studies variance *τ* ^2^. Further, for large sample sizes, estimated LORs are approximately normally distributed, ${\hat{\theta }}_{j}∼N(\theta_{j},\sigma_{j}^{2})$. Therefore, the REM considers that ${\hat{\theta }}_{j}∼N(\theta ,\sigma_{j}^{2}+\tau^{2})$, and the FEM follows for *τ* ^2^=0. Importantly, the variances $\sigma_{j}^{2}$ are of order *O*(1/*N*_*j*_) for sample sizes *N*_*j*_,*j*=1,⋯,*K* of the studies. Standard inference concerns the combined effect $\hat{\theta }$, estimated as the weighted mean of the individual effects

(1.1) $${\hat{\theta }}_{IV}=\overset{K}{\underset{j=1}{\sum }}w_{j}{\hat{\theta }}_{j}/\overset{K}{\underset{j=1}{\sum }}w_{j},$$

with weights equal to inverse estimated variances, $w_{j}={\hat{\sigma }}_{j}^{-2}$ in FEM, and $w_{j}={\left({\hat{\sigma }}_{j}^{2}+{\hat{\tau }}^{2}\right)}^{-1}$ in REM. The distribution of the combined effect $\hat{\theta }$ is customarily approximated by a normal distribution, $N(\theta ,{\left(\sum_{j=1}^{K}w_{j}\right)}^{-1})$. Estimated within‐studies variances ${\hat{\sigma }}_{j}^{2}$ are often assumed to be known. Establishing an effect of treatment corresponds to testing the null hypothesis *θ*=0, and a confidence interval calculation in REM requires an estimate of the between‐studies variance *τ* ^2^, which is also of interest for quantifying heterogeneity. See 1 for further details on traditional meta‐analytic techniques.

The shortcomings of the inverse‐variance method, as described earlier, in meta‐analysis in general and in its application to the LORs are well known. They include the bias in estimation of the combined effect, underestimation of its variance, and poor coverage of the obtained confidence intervals, especially for sparse data and/or small sample sizes, see 2 for discussion and further references. Under FEM, a considerably better way to combine ORs is the Mantel‐Haenzsel (1959) 3 method. Unfortunately, there is no analogue to this method under the REM.

Further, the most popular method of estimating the between‐studies variance *τ* ^2^ in REM is the DerSimonian and Laird (1986) 4 method, based on the approximate moments of the Cochran's *Q* statistic, 5, but this method is not satisfactory, both in general and in application to the heterogeneity estimation of LORs, see 6, 7, 8. 7 recommend the use of the Breslow‐Day (BD) test 9 for testing the heterogeneity of ORs, and also provide a new gamma‐based approximation to the distribution of *Q*.

Alternative approaches to REM include the use of fixed weights 10, 11 and the overdispersion model (ODM) introduced by 12. The ODM allows the interpretation of overdispersion through intra‐cluster correlation (ICC) *ρ* or its transformation.

In this paper, we study the beta‐binomial (BB) model, an important particular case of the ODM for ORs. Both REM and the BB model are compound random‐effects models. Both models include binomial distributions for positive responses in both arms, conditional on the probabilities. The standard REM accounts for between‐study variation imposing a normal distribution on LORs, or on log‐odds in treatment and control arms 13. Similarly, the BB model includes beta‐distributed variation of the probabilities of events in one or both arms across the studies.

For the log‐odds‐ratios from a pair of BB distributions, a normal approximation has been suggested by 14 and 15. To obtain the combined effect, we study the standard inverse‐variance method and a version of the Mantel‐Haenszel (MH) method adjusted for clustering, 16. Both methods require estimation of the ICC *ρ*. We study several methods of estimating *ρ*, including two new methods, one based on the profiling of the BD test, and another based on the gamma approximation to the distribution of *Q* by 7.

The structure of this paper is as follows. In Section 2, we briefly provide some background for methods of meta‐analysis of LORs. The proposed BB model for meta‐analysis of ORs and the MH‐inspired estimation of the combined OR are introduced in Section 3. Five methods for estimation of an overdispersion parameter *ρ*, including a new method based on the BD test, are given in Section 4. An example is provided in Section 5. A large simulation study is described in Section 6. Discussion and conclusions are in Section 7.

## 2 Background on meta‐analysis of 2×2 tables

### 2.1 Fixed effect model

Consider *K* comparative studies reporting summary binary outcomes. The data from each study, *j*=1,⋯,*K*, constitutes a pair of independent binomial variables, *X*_1*j*_ and *X*_2*j*_, the numbers of events out of *n*_1*j*_ and *n*_2*j*_ subjects in the treatment and control arms

$$X_{1j}∽\text{Binom}(n_{1j},p_{1j})\;\text{and}\;X_{2j}∽\text{Binom}(n_{2j},p_{2j}),$$

where *p*_*ij*_ for *i*=1,2 are the risks in the treatment and the control arms, respectively.

In meta‐analysis of these data, the effect measure that we focus on is the logarithm of the OR, $\theta_{j}=log(\psi_{j})$, where the OR for study *j* is

(2.1) $$\psi_{j}=\frac{p_{1j}(1-p_{2j})}{p_{2j}(1-p_{1j})}\;\text{estimated by}\;{\hat{\psi }}_{j}=\frac{X_{1j}(n_{2j}-X_{2j})}{X_{2j}(n_{1j}-X_{1j})}.$$

The approximate variance of the log‐odds‐ratio ${\hat{\theta }}_{j}=log({\hat{\psi }}_{j})$ derived by the delta method is

(2.2) $$\sigma_{j}^{2}=\mathrm{Var}({\hat{\theta }}_{j})=\frac{1}{n_{1j}p_{1j}(1-p_{1j})}+\frac{1}{n_{2j}p_{2j}(1-p_{2j})}.$$

For a study of sample size *N*_*j*_=*n*_1*j*_+*n*_2*j*_, this variance is of order *O*(1/*N*_*j*_). The variance is estimated by substituting the estimates ${\hat{p}}_{1j}=X_{1j}/n_{1j}$ and ${\hat{p}}_{2j}=X_{2j}/n_{2j}$ in (2.2), 17, resulting in

(2.3) $${\hat{\sigma }}_{j}^{2}=\mathrm{Var}({\hat{\psi }}_{j})=\frac{1}{X_{1j}}+\frac{1}{X_{2j}}+\frac{1}{n_{1j}-X_{1j}}+\frac{1}{n_{2j}-X_{2j}}.$$

To reduce the bias of $\hat{\psi }$ and its estimated variance, or in the case of zero entries, a correction *a* is usually added to each cell. The most common choice is *a*=1/2 18, but alternatives are available, 19.

The FEM assumes homogeneity of ORs across the studies. The inverse‐variance‐weighted method for pooling LOR estimates from individual studies uses ${\hat{\theta }}_{IV}$ given by (1.1) with weights $w_{j}={\hat{\sigma }}_{j}^{-2}$.

An attractive alternative is the MH method, introduced by 3 to combine ORs from stratified 2×2 contingency tables. The MH estimator for pooling ORs ${\hat{\psi }}_{j}$ from *K* tables is

(2.4) $${\hat{\psi }}_{MH}=\frac{\overset{K}{\underset{j=1}{\sum }}X_{1j}(n_{2j}-X_{2j})/N_{j}}{\overset{K}{\underset{j=1}{\sum }}X_{2j}(n_{1j}-X_{1j})/N_{j}}=\frac{\overset{K}{\underset{j=1}{\sum }}W_{j}{\hat{\psi }}_{j}}{\overset{K}{\underset{j=1}{\sum }}W_{j}},$$

with weights $W_{j}=\left[\right.\frac{1}{n_{1j}}+\frac{1}{n_{2j}}{]}^{-1}(1-{\hat{p}}_{1j}){\hat{p}}_{2j}$ for the ORs ${\hat{\psi }}_{j}$ given by (2.1). The advantages of MH estimator are its robustness and its ability to handle empty cells without corrections, 20. The variance of the MH estimator, for both sparse data ( K→∞) and large‐strata ( $N_{j}\to \infty$) limiting models was derived by 21 and 22 and can be used to obtain confidence intervals for LOR.

### 2.2 Random effects models

The FEM is usually too restrictive. REMs take into account between‐studies heterogeneity, usually by introducing a mixing distribution for *θ*_*j*_, or by directly inflating the within‐study variances. The standard REM described in Section 1 uses a normal mixing distribution. More generally, let individual

(2.5) $${\hat{\theta }}_{j}∽F(\theta_{j},\nu_{j})\;\text{and}\;h(\theta_{j})∽G(h(\theta ),\tau^{2}),$$

where *θ*_*j*_ and *ν*_*j*_ are within‐study parameter of interest and (if relevant) a nuisance parameter, (for instance, location and scale parameters), *θ* is the overall parameter of interest, *h*(·) is a transformation, and *τ* ^2^ is an unknown variance parameter of the between‐study mixing distribution *G*, describing the variability and heterogeneity of the effect measures. The distributions *F* and *G* are commonly assumed to be normal. However, other combinations of distributions are possible. Examples of non‐normal *G* distributions include *t*, skewed normal or skewed *t*, whereas *F* can be binomial, 23. For a binomial distribution *F*, the transformation *h* can be a log or log‐odds transformation. The REM for ORs discussed by 13 combines an exact non‐central hypergeometric distribution for *F* with a normal mixing distribution for *G*. This hypergeometric‐normal model is a generalized linear mixed model.When both *F* and *G* are normal and $\nu_{j}=\sigma_{j}^{2}$, the standard REM is obtained. In this case, the marginal REM for an estimated effect measure ${\hat{\theta }}_{j}$ in study *j* is

(2.6) $${\hat{\theta }}_{j}∽N(\theta ,\sigma_{j}^{2}+\tau^{2}),\;\text{for}\;j=1,\ldots ,K.$$

For the estimated log‐odds‐ratio ${\hat{\theta }}_{j}$, the within‐study variance $\sigma_{j}^{2}$ is approximated by (2.2). For the case of risk difference, 24 discuss the dangers of treating the within‐study variances as known. The main concern in the REM lies in estimating the unknown between‐studies variance *τ* ^2^. A variety of moment‐based or likelihood‐based estimators of *τ* ^2^ have been proposed, see 25, 26. Among the moment‐based estimators, the one derived by 4 is commonly used in practise. Among the likelihood based estimators, the restricted maximum likelihood (REML) estimator is popular because of its reduced bias. An alternative approach to REM, aimed at incorporating heterogeneity through overdispersion, was proposed by 27 and found advantageous by 28. 27 introduced a multiplicative REM in the form

(2.7) $${\hat{\theta }}_{j}∽N(\theta ,\varphi \sigma_{j}^{2}),$$

where *φ* is a multiplicative random effects parameter. This parameter allows deflation and inflation in the variance of ${\hat{\theta }}_{j}$.

A new REM for meta‐analysis based on overdispersion is proposed by 12. In their model, the multiplicative parameter *φ* becomes study‐specific and is defined by *φ*_*j*_=1+*a*_*j*_ *γ*, where *a*_*j*_=*a*(*N*_*j*_) is a linear function of sample size *N*_*j*_. The advantage of this model is an interpretation of the overdispersion parameter *γ* as an ICC or a transformation of it. For comparative studies, the within‐study variance $\sigma_{j}^{2}$ can be written as $\sigma_{j}^{2}=v_{j}(R_{j})/N_{j}$, where *R*_*j*_=*n*_1*j*_/*n*_2*j*_ is the allocation ratio of treatment to control group sizes and *v*_*j*_ is some function of *R*_*j*_ and relevant distribution parameters of order *O*(1). The ODM by 12 for two‐sample effect measures is

(2.8) $${\hat{\theta }}_{j}∽N\left(\theta ,\frac{v_{j}(R_{j})}{N_{j}}(1+a_{j}\gamma )\right)\;\text{for}\;\gamma >-\frac{1}{\max(a_{j})}.$$

The variance in this model consists of two parts: the FEM variance *v*_*j*_(*R*_*j*_)/*N*_*j*_ and the variance‐inflation term [*v*_*j*_(*R*_*j*_)*a*_*j*_/*N*_*j*_]*γ* of order *O*(1). Therefore, this model is intermediate between the standard additive REM (2.6), which adds the constant term *τ* ^2^ of order *O*(1) to each within‐study variance, and the multiplicative REM (2.7), which, inflating variances by a constant factor *φ*, keeps them of order *O*(1/*N*_*j*_), similar to the FEM.

## 3 Overdispersed binary data

### 3.1 The beta‐binomial distribution

The BB distribution is obtained by mixing a binomial distribution Binom (*n*,*p*) by a beta distribution for its success probability *p*. When *Y*∼ Binom (*n*,*p*) and *p*∼ Beta (*α*,*β*), then unconditionally, *Y* follows a BB distribution with parameters *α*,*β*, and *n*. This distribution arises naturally in Bayesian statistics as the beta distribution is the conjugate prior distribution for the parameter *p* if the data are binomial. The expected value and variance of *Y* are

$$E(Y)=\frac{n\alpha }{\alpha +\beta },\;\mathrm{Var}(Y)=\frac{n\alpha \beta (n+\alpha +\beta )}{{\left(\alpha +\beta \right)}^{2}(\alpha +\beta +1)}.$$

It is more convenient to re‐parametrize this distribution as BetaBinom (*n*,*π*,*ρ*), where *π*=*α*/(*α*+*β*) and *ρ*=1/(*α*+*β*+1). Then

(3.1) E(Y)=nπ,Var(Y)=nπ(1−π)(1+(n−1)ρ),

which shows the BB distribution to be an overdispersed binomial distribution. A distribution with the same two moments can be obtained as the distribution of the sum of *n* Bernoulli(*π*) random variables with ICC *ρ*. Such distributions may be obtained through various generation mechanisms 29, 30 and differ in the higher moments. To guarantee approximate normality of LORs, we consider only the BB distribution. This distribution is widely used in combining overdispersed binomial data, see, for example, 31.

### 3.2 Odds‐ratios under beta‐binomial model

In the context of meta‐analysis, we assume that the observations in the treatment and control arms of each study are independent, and that within each arm, the observations follow a BB distribution with the same parameter *ρ*, that is,

$$X_{1j}∽\text{BetaBinom}(n_{1j},p_{1j},\rho )\;\text{and}\;X_{2j}∽\text{BetaBinom}(n_{2j},p_{2j},\rho ).$$

This is equivalent to requiring that the sum of parameters *α*+*β* of the mixing beta distributions not differ between the two arms. The ORs *ψ*_*j*_ and their estimates ${\hat{\psi }}_{j}$ are defined as in (2.1), and the log‐odds‐ratios ${\hat{\theta }}_{j}$ are approximately normally distributed. However, the variances of individual ORs and log‐odds‐ratios have to be adjusted for overdispersion. The adjusted approximate variance of LOR, obtained by using the delta method, is

(3.2) $$\mathrm{Var}({\hat{\theta }}_{j})=\frac{1+(n_{1j}-1)\rho }{n_{1j}p_{1j}(1-p_{1j})}+\frac{1+(n_{2j}-1)\rho }{n_{2j}p_{2j}(1-p_{2j})},$$

with restriction for *ρ*

$$\rho >\max_{1⩽\;j⩽K}\left\{-\frac{1}{n_{1j}-1},\;\;-\frac{1}{n_{2j}-1}\right\}.$$

For *ρ*=0 this model is the standard FEM for binomially distributed data. In this case, the variance of LOR (2.2) can be written as $\sigma_{j}^{2}=v_{j}(R_{j})/N_{j}$ with

$$v_{j}(R_{j})=(R_{j}+1)\left(\frac{1}{R_{j}p_{1j}(1-p_{1j})}+\frac{1}{p_{2j}(1-p_{2j})}\right).$$

Substituting this expression into (3.2), the variance of LOR for overdispersed binomial data is

$$\mathrm{Var}({\hat{\theta }}_{j})=\frac{v_{j}(R_{j})}{N_{j}}+\left[\frac{\left(n_{1j}-1\right)}{n_{1j}p_{1j}(1-p_{1j})}+\frac{\left(n_{2j}-1\right)}{n_{2j}p_{2j}(1-p_{2j})}\right]\rho .$$

This variance is clearly inflated in comparison with the standard variance (2.2). For large *n*_1*j*_ and *n*_2*j*_, the inflation term is $\left[{\left[p_{1j}(1-p_{1j})\right]}^{-1}+{\left[p_{2j}(1-p_{2j})\right]}^{-1}\right]\rho$, therefore it is of order *O*(1) and increases with ICC. It also may be large for probabilities in either arm close to 0 or 1.

Defining

(3.3) $$\tau_{j}^{2}=\left[\frac{\left(n_{1j}-1\right)}{n_{1j}p_{1j}(1-p_{1j})}+\frac{\left(n_{2j}-1\right)}{n_{2j}p_{2j}(1-p_{2j})}\right]\rho ,$$

it is clear that when both parts of (3.3) do not depend on *j*, the BB model results in the same first and second moments of the LORs ${\hat{\theta }}_{j}$ as the standard REM, with the relation between *ρ* and *τ* ^2^ given by (3.3). This is the case when the probabilities in the treatment and control arms do not differ, *p*_*ij*_≡*p*_*i*_, and the sample sizes are all equal, or are large enough so that (*n*_*ij*_−1)/*n*_*ij*_≈1.

The variance of the sample log‐odds‐ratio can also be written in the ODM (2.8) form as $\mathrm{Var}({\hat{\theta }}_{j})=(v_{j}(R_{j})/N_{j})(1\;+\;a_{j}\rho )$ for

$$a_{j}=\frac{N_{j}R_{j}[{\left(1-p_{2j}(1-\psi_{j})\right)}^{2}+\psi_{j}]}{\left(R_{j}+1)[{\left(1-p_{2j}(1-\psi_{j})\right)}^{2}+R_{j}\psi_{j}\right]}-1.$$

Thus, *a*_*j*_ is a linear function of *N*_*j*_ and has the same order as *N*_*j*_. For balanced studies *R*_*j*_=1, and *a*_*j*_ simplifies to *a*_*j*_=*N*_*j*_/2−1.

An alternative model considers overdispersion only in the treatment arm:

$$X_{1j}∽\text{BetaBinom}(n_{1j},\alpha_{1j},\beta_{1j}),\;X_{2j}∽\text{Bin}(n_{2j},p_{2j})$$

This is perhaps closer to the standard REM, which usually has a random effect only in the treatment arm 27. In this case, the variance for OR is

$$\mathrm{Var}({\hat{\psi }}_{j})=\frac{1+(n_{1j}-1)\rho }{n_{1j}p_{1j}(1-p_{1j})}+\frac{1}{n_{2j}p_{2j}(1-p_{2j})},$$

which is still inflated, in comparison to the FEM variance, by the term [(*n*_1*j*_−1)/(*n*_1*j*_ *p*_1*j*_(1−*p*_1*j*_))]*ρ* . Subsequent methods are easily adapted to this version of the BB model, and we do not pursue this model further.

### 3.3 Adjusted Mantel‐Haenszel method for combining odds ratios

Applying the standard MH method for overdispersed binary data results in a bias and a wrong variance of the combined OR, and a wrong type I error of the associated test, 32. An adjusted version of the MH test and related estimator of the combined OR appropriate for the meta‐analysis of cluster‐randomized trials were studied by 16, 33, 32. In cluster‐randomized trials, arm *i* of trial *j* contains *m*_*ij*_ clusters of size *n*_*ij*_, and an ICC *ρ*_*j*_ is common to all clusters in trial *j*. This can be adapted to a case of one cluster in each arm, which is equivalent to the ODM by 12. This adapted MH method is as follows.

Introduce a set of correction factors

(3.4) $$C_{ij}=1+(n_{ij}-1)\rho \;\;\text{for}\;\;i=1,2;\;j=1,\cdots \;,K.$$

These are referred to as design effects by 34. Next, define the adjusted weights for the ORs ${\hat{\psi }}_{j},\;j=1,\cdots \;,K$

$$W_{jC}=\left[\right.\frac{C_{1j}}{n_{1j}}+\frac{C_{2j}}{n_{2j}}{]}^{-1}(1-{\hat{p}}_{1j}){\hat{p}}_{2j}.$$

The corrected MH estimate of the OR for overdispersed binomial data is

(3.5) $${\hat{\psi }}_{cMH}=\frac{\overset{K}{\underset{j=1}{\sum }}W_{jC}{\hat{\psi }}_{j}}{\overset{K}{\underset{j=1}{\sum }}W_{jC}}\;\text{for}\;{\hat{\psi }}_{j}=\frac{(1-{\hat{p}}_{2j}){\hat{p}}_{1j}}{(1-{\hat{p}}_{1j}){\hat{p}}_{2j}}.$$

We propose to use this corrected version of the MH estimator for combining ORs in the BB model. When there is no overdispersion, *ρ*=0,*C*_*ij*_=1 and the expression (3.5) reduces to standard MH estimator (2.4) for the fixed effects model.When $\rho \to -1/\max(a_{j})$,

$${\hat{\psi }}_{cMH}\to \frac{\underset{n_{1j}=n_{2j}=\max(n_{j})}{\sum }n_{1j}n_{2j}p_{1j}(1-p_{2j})/(n_{1j}+n_{2j})}{\underset{n_{1j}=n_{2j}=\max(n_{j})}{\sum }n_{1j}n_{2j}p_{2j}(1-p_{1j})/(n_{1j}+n_{2j})},$$

which is the standard MH estimator for the subset of the studies of the largest sample size. The proof is provided in the Web Appendix.To obtain the asymptotic variance of ${\hat{\psi }}_{cMH}$, we adjusted for overdispersion the asymptotic variance of the MH estimator derived by 21 and 22:

$$\mathrm{Var}({\hat{\psi }}_{cMH})=\frac{\overset{K}{\underset{j=1}{\sum }}R_{j}P_{j}}{2R^{2}}+\frac{\overset{K}{\underset{j=1}{\sum }}(P_{j}S_{j}+Q_{j}R_{j})}{2RS}+\frac{\overset{K}{\underset{j=1}{\sum }}S_{j}Q_{j}}{2S^{2}},$$

where

$$\begin{array}{ccc} P_{j} & =\frac{C_{1j}(n_{2j}-X_{2j})+C_{2j}X_{1j}}{C_{1j}n_{2j}+C_{2j}n_{1j}},\;\;R_{j}=\frac{(n_{2j}-X_{2j})X_{1j}}{C_{1j}n_{2j}+C_{2j}n_{1j}}, & \\ Q_{j} & =\frac{C_{2j}(n_{1j}-X_{1j})+C_{1j}X_{2j}}{C_{1j}n_{2j}+C_{2j}n_{1j}},\;\;S_{j}=\frac{(n_{1j}-X_{1j})X_{2j}}{C_{1j}n_{2j}+C_{2j}n_{1j}}, \end{array}$$

and $R=\sum_{j=1}^{K}R_{j}$ and $S=\sum_{j=1}^{K}S_{j}$.

## 4 Estimation of *ρ*

To evaluate the corrected MH estimator (3.5), an estimate of the ICC *ρ* is required. We consider two modifications of established methods, namely a moment estimator based on Cochran's *Q* statistic similar to the DerSimonian and Laird (1986) 4 estimator of *τ* ^2^, and a REML estimator. We also consider related confidence intervals: an interval based on profiling the *Q* statistic as in 35 and a REML‐based interval. According to 35, these two approaches perform the best for estimation of the between‐studies variance *τ* ^2^ in the additive REM. We also use a version of the Mandel and Paule (1970) 36 method to estimate *ρ* from the large‐sample approximation of *Q* by the *χ* ^2^ distribution. All three methods were proposed in 12 for estimation of *ρ*, but have not so far been explored by simulation. However, the chi‐square distribution is a poor approximation to the distribution of the *Q* statistic for LORs 7, and the modified *Q* test based on the improved gamma approximation developed by 7 and the Breslow and Day (1980) 9 (BD) test are attractive alternatives for testing the heterogeneity of ORs. In the section, we propose two new methods of point and interval estimation of *ρ*, based on inverting the modified *Q* test and the BD test. The point estimation is based on an adaptation of the Mandel and Paule (1970) 36 method, and the interval estimation is achieved through profiling the modified *Q* and BD tests.

### 4.1 Restricted maximum likelihood estimation of *ρ*

The restricted likelihood for the normal distribution with mean *θ* and variances *v*_*j*_(1+*a*_*j*_ *ρ*)/*n*_*j*_ is

(4.1) $$l_{R}(\rho ,\theta )=-\frac{1}{2}log\left(\sum \overset{*}{\underset{j}{w}}\right)-\frac{1}{2}\sum \overset{*}{\underset{j}{w}}{\left(\theta_{j}-\theta \right)}^{2}+\frac{1}{2}\sum log(\overset{*}{\underset{j}{w}}),$$

for inverse‐variance weights *w* *j*∗=*w*_*j*_/(1+*a*_*j*_ *ρ*). Following 12, the REML equation for *ρ* is

(4.2) $${\left(W^{*}\right)}^{-1}\sum \overset{*}{\underset{j}{w}}\frac{a_{j}}{1+a_{j}\rho }+\sum \overset{*}{\underset{j}{w}}{\left(\theta_{j}-\theta \right)}^{2}\frac{a_{j}}{1+a_{j}\rho }=\sum \frac{a_{j}}{1+a_{j}\rho },$$

where $W^{*}=\sum w_{j}^{*}$. The mean *θ*_*REML*_ is obtained as $\theta_{REML}=\sum w_{j}^{*}{\hat{\theta }}_{j}/W^{*}$, and an iterative procedure readily yields a solution, denoted by ${\hat{\rho }}_{REML}$.

The REML confidence intervals are given by all values of *ρ* that satisfy

(4.3) $$l_{R}(\rho )⩾l_{R}({\hat{\rho }}_{REML})-\chi_{1;1-\alpha }^{2}/2,$$

where $\chi_{1;1-\alpha }^{2}$ is the (1−*α*) quantile of the chi‐square distribution with 1 degree of freedom. We will see in Section 6 that the REML‐based point and interval estimators of *ρ* are generally inferior to other methods, and we will not recommend them.

### 4.2 *Q*‐statistic‐based estimation of *ρ*

Cochran's statistic is $Q=\sum {ŵ}_{j}{\left({\hat{\theta }}_{j}-{\bar{\theta }}_{w}\right)}^{2}$, for the inverse‐variance weights ${ŵ}_{j}={\hat{\sigma }}_{j}^{-2}$ and ${\bar{\theta }}_{w}=\sum {ŵ}_{j}{\hat{\theta }}_{j}/\sum {ŵ}_{j}$. Under the null hypothesis of no over‐ or underdispersion *ρ*=0, the *Q*‐statistic is approximately chi‐square distributed with *K*−1 degrees of freedom, so that E(*Q*)=*K*−1. This approximation is extremely conservative whenever either binomial parameter is far from 0.5, but its performance is reasonable when the binomial parameters are relatively close to 0.5, 7. Under the ODM (2.8),

(4.4) $$E(Q)=K-1+(Kā-{ā}_{w})\rho ,$$

where $ā=\sum a_{j}/K,{ā}_{w}=\sum w_{j}a_{j}/W$, and $W=\sum w_{j}$, 12. The estimate of *ρ* from (4.4) should satisfy the condition $\hat{\rho }>-1/\max(a_{j})$.

A moment (M) estimator in the spirit of DerSimonian and Laird (1986) 4 proposed by 12 is

(4.5) $${\hat{\rho }}_{M}=\max\left(\frac{Q-(K-1)}{Kā-{ā}_{w}},-\frac{1}{\max(a_{j})}\right);$$

underdispersion may be present for *Q*<*K*−1. If only positive values of *ρ* are acceptable, then $\hat{\rho }$ can be truncated at zero.

When the correct weights *w* *j*∗=*w*_*j*_(*ρ*)=*w*_*j*_/(1+*a*_*j*_ *ρ*) are used, ${\bar{\theta }}_{w^{*}}=\sum {ŵ}_{j}^{*}{\hat{\theta }}_{j}/\sum {ŵ}_{j}^{*}$, and the corrected *Q* statistic given by $Q^{*}(\rho )=\sum w_{j}^{*}{\left({\hat{\theta }}_{j}-{\bar{\theta }}_{w^{*}}\right)}^{2}$ has approximately the $\chi_{K-1}^{2}$ distribution.

The Mandel and Paule (1970) 36 method of estimating between‐studies variance *τ* ^2^ in the standard REM was studied subsequently by 37 and 25. This method uses the approximate chi‐square distribution $\chi_{K-1}^{2}$ of the Cochran's *Q* statistic under homogeneity to find an estimate of *τ* ^2^. In our context, the estimating equation *Q* ^∗^(*ρ*)=*K*−1 provides another moment‐based estimator of *ρ* in the spirit of Mandel and Paule (1970) 36, also proposed by 12. We refer to this estimator as *ρ*_*MP*_.

A related confidence interval is obtained by inverting the *Q* test. The confidence interval is constructed as

(4.6) $$\left\{\rho >-1/\max(a_{j}):\chi_{K-1;\alpha /2}^{2}⩽Q^{*}(\rho )⩽\chi_{K-1;1-\alpha /2}^{2}\right\},$$

where $\chi_{K-1,\alpha }^{2}$ are the quantiles of the *χ* ^2^ distribution with *K*−1 degrees of freedom. 35 shows that the standard REM confidence intervals for *τ* ^2^ based on this approach, named *Q*‐profile, perform very well, better than the REML confidence intervals described in Section 4.1. However, we will see in Section 6 that the methods introduced in this section are generally inferior to the methods based on the corrected distribution of *Q* or on the BD test, described in Sections 4.3 and 4.4, respectively, and we will not recommend them.

### 4.3 Corrected *Q*‐statistic based estimation of *ρ*

According to 7, the distribution of the *Q* statistic can be well approximated by a gamma distribution with shape and scale parameters

$$r(\rho )=\frac{E{\left(Q\right)}^{2}}{\mathrm{Var}(Q)}\;\text{and}\;\lambda (\rho )=\frac{\mathrm{Var}(Q)}{E(Q)}.$$

The expected value and variance of the *Q* statistic for log‐odds‐ratio based on this gamma approximation can be estimated from the relations

(4.7) $$\left(K-1)-E(Q)=0.678[(K-1)-E_{th}(Q)\right]$$

and

$$\mathrm{Var}(Q)=4.74(K-1)-12.17E[Q]+9.42E{\left[Q\right]}^{2}/(K-1),$$

where E_*th*_(*Q*) is the theoretical approximation to the mean of *Q* for log‐odds‐ ratio, 7. The Mandel‐Paule estimator of *ρ* based on the gamma approximation to the distribution of the *Q* statistic, denoted by ${\hat{\rho }}_{cMP}$, is found from *Q* ^∗^(*ρ*)=*E*(*Q*), given that a solution exists, where *E*(*Q*) is the solution of equation  (4.7). The related confidence interval based on the gamma approximation to the distribution of *Q* statistic can be obtained from

(4.8) $$\left\{\rho >-1/\max(a_{j}):\Gamma_{r(\rho ),\lambda (\rho );\alpha /2}⩽Q^{*}(\rho )⩽\Gamma_{r(\rho ),\lambda (\rho );1-\alpha /2}\right\},$$

where Γ_*r*(*ρ*),*λ*(*ρ*);*α*_ are the quantiles of the gamma distribution with *r*(*ρ*) and *λ*(*ρ*) as shape and scale parameters. We will see from the simulations described in Section 6 that this method performs well when sample sizes are small (up to 100).

### 4.4 Breslow‐Day‐based estimation of *ρ*

The BD test is based on the statistic

(4.9) $$X_{BD}^{2}=\overset{K}{\underset{j=1}{\sum }}\frac{{\left(X_{1j}-E(X_{1j}|{\hat{\psi }}_{MH})\right)}^{2}}{\mathrm{Var}(X_{1j}|{\hat{\psi }}_{MH})}∽\chi_{K-1}^{2},$$

where $E(X_{1j}|{\hat{\psi }}_{MH})$ and $\mathrm{Var}(X_{1j}|{\hat{\psi }}_{MH})$ denote the expected number and the asymptotic variance, respectively, of the number of cases in the treatment arm under the assumption of homogeneity of odds ratios, given the fitted MH OR ${\hat{\psi }}_{MH}$. The expected value $E(X_{1j}|{\hat{\psi }}_{MH})$ in (4.9) is obtained from the quadratic equation

(4.10) $$\frac{E(X_{1j}|{\hat{\psi }}_{MH})[N_{j}-m_{1j}-n_{1j}+E(X_{1j}|{\hat{\psi }}_{MH})]}{\left[m_{1j}-E(X_{1j}|{\hat{\psi }}_{MH})][n_{1j}-E(X_{1j}|{\hat{\psi }}_{MH})\right]}={\hat{\psi }}_{MH},$$

where *m*_1*j*_=*X*_1*j*_+*X*_2*j*_. Its asymptotic variance $\mathrm{Var}(X_{1j}|{\hat{\psi }}_{MH})$ is a particular case, for *ρ*=0, of the variance of *X*_1*j*_ under overdispersion given by 38

(4.11) $$\begin{array}{ccc} \mathrm{Var}(X_{1j}|{\hat{\psi }}_{MH}) & =\left[\right.\frac{1}{E(X_{1j}|{\hat{\psi }}_{MH})C_{1j}}+\frac{1}{(m_{1j}-E(X_{1j}|{\hat{\psi }}_{MH}))C_{2j}} & \\ & \;+\frac{1}{(n_{1j}-E(X_{1j}|{\hat{\psi }}_{MH}))C_{1j}}+\frac{1}{(N_{j}-m_{1j}-n_{1j}+E(X_{1j}|{\hat{\psi }}_{MH}))C_{2j}}{]}^{-1}, \end{array}$$

where the *C*_*ij*_ are given by equation  (3.4). The asymptotic variance given in (4.11) is not defined when any of the cells of the *j*‐th table are empty. In these cases, a correction of 0.5 is added to each cell of the table.

The BD statistic $X_{BD}^{2}=X_{BD}^{2}(\rho )$ is now a function of *ρ*, and $X_{BD}^{2}(\hat{\rho })$ has a $\chi_{K-1}^{2}$ distribution under the homogeneity of ORs when the value of *ρ* is estimated correctly. Equating the BD statistic to its first moment,

(4.12) $$\overset{K}{\underset{j=1}{\sum }}\frac{{\left(X_{1j}-E(X_{1j}|{\hat{\psi }}_{MH})\right)}^{2}}{\mathrm{Var}(X_{1j}|{\hat{\psi }}_{MH})}=K-1,$$

and solving this estimating equation for $\hat{\rho }$ result in the Mandel‐Paule‐type estimator ${\hat{\rho }}_{BD}$, which can be used for the calculation of ${\hat{\psi }}_{cMH}$ given by (3.5). The range of values for the overdispersion parameter *ρ* is the interval $\left(\max\{-1/a_{\max},-1/\max(n_{ij}-1)\},\;1\right)$. When $\rho \to -1/(n_{ij}-1)$, the variance in the denominator of $X_{BD}^{2}(\rho )$ converges to zero, and the BD statistic tends to infinity. When $X_{BD}^{2}(\rho =0)<K-1$, the solution of equation  (4.12) always exists and is negative. On the other hand, *ρ*=1 provides the lower limit for the BD statistic, so if this lower limit of $X_{BD}^{2}(\rho =1)>K-1$, the equation  (4.12) does not have a solution; in this case we set $\hat{\rho }=1$.

The confidence interval for *ρ* can be obtained by profiling the BD test, similarly to confidence interval for *τ* ^2^ obtained by profiling Cochran's *Q* under REM by 35. The confidence interval with 95% coverage probability for the ICC parameter *ρ* based on the modified BD test is given by

(4.13) $$\left\{1>\rho >\max\{-\frac{1}{a_{\max}},-\frac{1}{\max(n_{ij}-1)}\}:\;\overset{2}{\underset{K-1,0.025}{\chi }}⩽X_{BD}^{2}(\rho )⩽\chi_{K-1,0.975}^{2}\right\}.$$

We will see from the simulations described in Section 6 that the methods developed in this Section are best utilized when sample sizes are large (greater than about 100).

## 5 Example: effects of diuretics on pre‐eclampsia

A well‐known meta‐analysis of nine trials which include the total of 6942 patients, evaluated the effect of diuretics on pre‐eclampsia 39. These data have been studied repeatedly, for example in 40, 41, 35, and 12. The basic data with ORs and their logs are provided in [12, Table 2a] and are reproduced in the Web Appendix. These data demonstrate considerable heterogeneity in incidence of pre‐eclampsia in both the treatment and the control groups, 12, suggesting that the BB model may be appropriate. There is also considerable heterogeneity in effect sizes. The overall incidence of pre‐eclampsia varies from 0.015 in study 6 to 0.412 in study 9. The ORs of effect of diuretics vary from 0.229 in study 4, a study with high incidence of 0.308, to 2.971 in study 8, a study with low incidence of 0.038. Cochran's *Q*‐statistic is *Q*=27.265, and the total sample size *N*=6942. Estimated values of *τ* ^2^ for standard REM, and of *ρ* assuming the BB model and using various estimating methods are provided in Table 1. The Der‐Simonian‐Laird estimate of the variance component in standard REM is $\tau_{DL}^{2}=0.23$, and $\tau_{REML}^{2}=0.30$. For *τ* ^2^ estimation in REM, Q‐profile confidence intervals 35 are given for the DL, and profile likelihood confidence intervals 40 for the REML method. In BB model, five methods of estimation provide estimates of $\hat{\rho }$ varying from 0.008 for the moment estimator to 0.019 for the BD‐based estimator. The confidence interval for *ρ* is shortest for REML and longest for the BD estimator. These values are directly interpretable as the estimated ICCs and their confidence limits. To see the effect of these estimates of heterogeneity on the inference about the OR, we compare the corresponding estimates for LOR and OR, and their confidence intervals, in the same table. The OR is highest (0.672) in the FEM, and, not surprisingly, its confidence interval is the shortest. The OR is lowest (0.596) in the standard REM, and various estimators based on inverse variances or the MH method provide intermediate values of OR, the one based on MH and method‐of‐moments estimator *ρ*_*M*_ providing the highest value of OR, 0.652. For each estimator of *ρ*, the MH estimation of OR results in a somewhat higher value of OR than the inverse‐variance‐based estimation, with a somewhat shorter confidence interval for OR. The sample sizes are reasonably large in all included studies, and based on the results of simulations reported in Section 6, we recommend to use the estimated ICC ${\hat{\rho }}_{BD}=0.019$ and corresponding value of the pooled OR ${\hat{\psi }}_{IV}=0.622$ with confidence interval (0.389,0.994).In this example, the CI's for *ρ* and for *τ* ^2^ do not include 0 so the evidence for REM or BB is stronger compared with FEM. Unfortunately, it is very difficult to distinguish between the REM and BB (see Section D.4 in Web Appendix.)

**Table 1:** Values and confidence intervals for *ρ*, for log odds ratios and for odds ratios for diuretics on pre‐eclampsia example; FEM is the fixed effect, REM is the random effects, and BB is the beta‐binomial model. Heterogeneity parameter estimated is *τ* ^2^ in REM, and *ρ* in BB model. *L* and *U* are the lower and upper limits of the respective confidence intervals (CIs).

Model	Method	Hetero geneity	*L*	*U*	LOR	*L*	*U*	length of CI	OR	*L*	*U*
FEM		0.000			−0.398	−0.573	−0.223	0.530	0.672	0.564	0.800
REM	DL&IV	0.230	0.072	2.202	−0.517	−0.916	−0.117	0.799	0.596	0.400	0.889
REM	REML&IV	0.300	0.043	1.475	−0.518	−0.956	−0.080	0.876	0.596	0.384	0.923
BB	M&IV	0.008	0.002	0.095	−0.436	−0.792	−0.080	0.712	0.647	0.453	0.923
	M&MH				−0.427	−0.775	−0.080	0.695	0.652	0.461	0.923
BB	REML&IV	0.010	0.001	0.060	−0.447	−0.835	−0.059	0.776	0.640	0.434	0.942
	REML&MH				−0.431	−0.809	−0.053	0.756	0.650	0.445	0.949
BB	MP&IV	0.017	0.002	0.095	−0.469	−0.920	−0.018	0.902	0.626	0.399	0.982
	MP&MH				−0.459	−0.898	−0.020	0.879	0.632	0.407	0.981
BB	cMP&IV	0.018	0.003	0.094	−0.474	−0.942	−0.007	0.936	0.623	0.390	0.993
	cMP&MH				−0.472	−0.927	−0.016	0.911	0.624	0.396	0.984
BB	BD&IV	0.019	0.003	0.107	−0.475	−0.944	−0.006	0.938	0.622	0.389	0.994
	BD&MH				−0.463	−0.920	−0.021	0.899	0.630	0.399	0.980

## 6 Simulation study

In this section, we provide a simulation study to assess the performance of point and interval estimators of the overdispersion parameter *ρ* and the combined LOR *θ* in the BB model of meta‐analysis. We assess the bias of five point estimators of *ρ*: the moment method (4.5), the Mandel‐Paule‐inspired method *ρ*_*MP*_, the corrected Mandel‐Paule estimator based on the gamma approximation to *Q* distribution *ρ*_*cMP*_, the REML method (4.2), and the BD‐based method (4.12). We also assess four related confidence intervals for *ρ* (4.6), (4.8), (4.3), and (4.13) for their coverage at the 95% confidence level. Additionally, we compare two estimation methods for obtaining point and interval estimators of the combined odds ratio or its log, the inverse‐variance method ${\hat{\theta }}_{w}=\sum w_{i}(\rho ){\hat{\theta }}_{i}/\sum w_{i}(\rho )$ and the modified MH method (3.5). We combine the five above‐mentioned point estimators of *ρ* with these two methods of obtaining the combined effect $\hat{\theta }$, resulting in 10 possible combinations, and we assess these estimators of $\hat{\theta }$ for bias and for coverage.

Typically, small values of *ρ*, below 0.1, appear in bio‐medical applications, 42, 43. Overdispersion is mostly due to clustering by healthcare provider. However, our range of values of *ρ* up to 0.3 is comparable with *τ* ^2^ values of up to 5 in the standard REM for our choice of values of probabilities and LORs provided below. This correspondence between heterogeneity in the additive REM and BB model is given by equation  (3.3).

### 6.1 Simulation design

Sizes of the control and treatment groups were taken equal *n*_1*j*_=*n*_2*j*_=*n*_*j*_ and were generated from a normal distribution with mean *n* and variance *n*/4 rounded to the nearest integer and left truncated at 5. For a given probability *p*_2*j*_, the number of events in the control group *X*_2*j*_ was simulated from a BB (*n*_*j*_,*p*_2*j*_,*ρ*) distribution using the R package *emdbook* 44. The number of events in the treatment group *X*_1*j*_ was generated from a BB (*n*_*j*_,*p*_1*j*_,*ρ*) distribution with $p_{1j}=p_{2j}\exp(\theta )/(1-p_{2j}+p_{2j}\exp(\theta ))$ for a given LOR value of *θ*. When *ρ*=0, the numbers of events for treatment and control arm *X*_*ij*_ were generated from binomial distributions with sample size *n*_*j*_ and probabilities *p*_*ij*_, preserving the relation between the probabilities in the treatment and control arms.

The following configurations of parameters were included in the simulations. The number of studies *K*=(5,10,20,30,50,80); average sample sizes in each arm are *n*=(10,20,40,50,80,100,160,250,640,1000); overdispersion parameter *ρ* varies between 0 and 0.1 in steps of 0.01, and between 0.1 and 0.3 in steps of 0.05. The values of LOR *θ* vary from 0 to 3 in steps of 1. The probability in the control group *p*_2*j*_ takes values 0.1,0.2,0.4. A total of 10000 simulations were produced for each combination.

### 6.2 Simulation results

**Figure 1:**
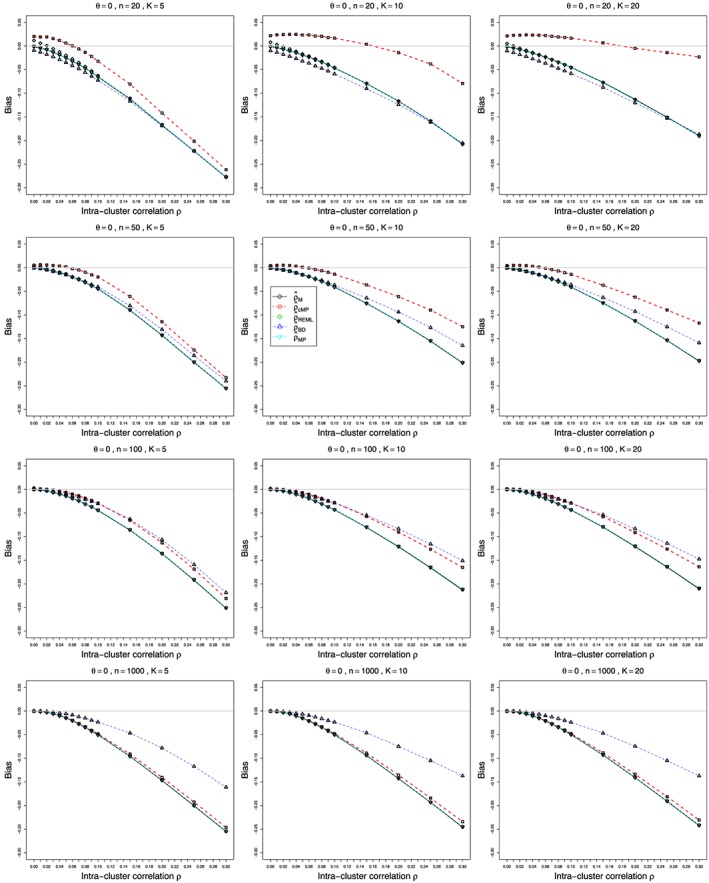
Bias estimated from *K* studies of the intra‐cluster correlation *ρ* in the beta‐binomial model for *p*_2*j*_=0.1, *θ*=0 and 0⩽ρ⩽0.3 for average sample sizes *n*=20,50,100, and 1000. Estimation methods: circles – Moment estimator ${\hat{\rho }}_{M}$, squares – Corrected Mandel‐Paule estimator ${\hat{\rho }}_{cMP}$), diamonds – ${\hat{\rho }}_{REML}$, triangles‐ Breslow‐Day based estimator ${\hat{\rho }}_{BD}$, reverse‐triangles – Mandel‐Paule estimator ${\hat{\rho }}_{MP}$. Light grey line at 0.

**Figure 2:**
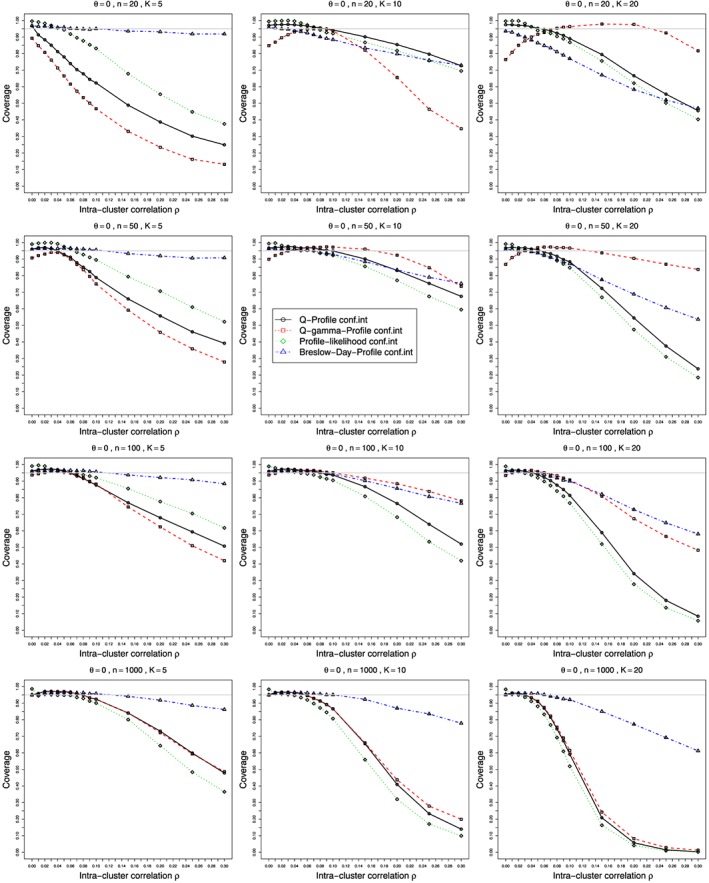
Coverage at the nominal confidence level of 0.95 of the intra‐cluster correlation *ρ* estimated from *K* studies in the beta‐binomial model for *p*_2*j*_=0.1, *θ*=0 and 0⩽ρ⩽0.3 for average sample sizes *n*=20,50,100, and 1000. Interval estimation methods: circles – Q‐profile confidence interval for *ρ* based on *χ* ^2^ distribution, squares – Q‐profile confidence interval for *ρ* based on Γ_*r*(*ρ*),*λ*(*ρ*)_ distribution), diamonds – Profile likelihood confidence intervals, triangles – Breslow‐Day‐Profile confidence intervals. Light grey line at 0.95.

**Figure 3:**
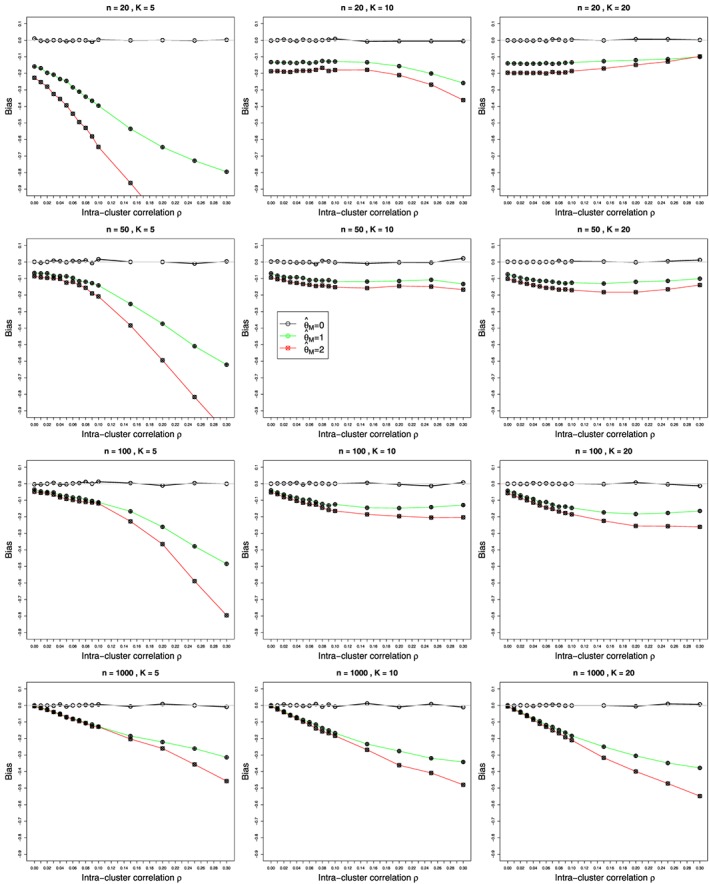
Bias of overall odds ratio ${\hat{\psi }}_{IV}$ obtained from *K* studies by the inverse‐variance method with the moment estimator ${\hat{\rho }}_{M}$ in the weights, for *p*_2*j*_=0.1, and 0⩽ρ⩽0.3 for average sample sizes *n*=20,50,100, and 1000. The biases are given for *θ*=0 (circles), *θ*=1 (circle plus), and *θ*=2 (circle cross). Light grey line at 0.

**Figure 4:**
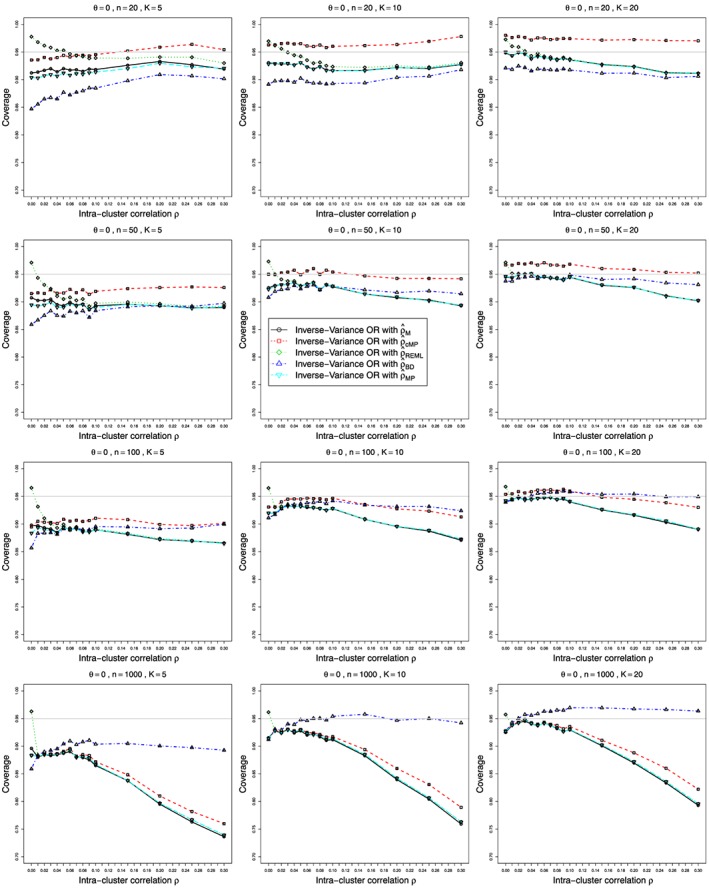
Coverage at the nominal confidence level of 0.95 of the overall odds ratio *ψ* obtained from *K* studies by the inverse‐variance method, for *p*_2*j*_=0.1, *θ*=0 and 0⩽ρ⩽0.3. The inverse‐variance weights use the following estimators of *ρ*: circles – ${\hat{\rho }}_{M}$, squares – Corrected Mandel‐Paule estimator ${\hat{\rho }}_{cMP}$, diamonds – restricted maximum likelihood estimator ${\hat{\rho }}_{REML}$, triangles – Breslow‐Day estimator ${\hat{\rho }}_{BD}$ and reverse‐triangles – Mandel‐Paule estimator ${\hat{\rho }}_{MP}$. Light grey line at 0.95.

**Figure 5:**
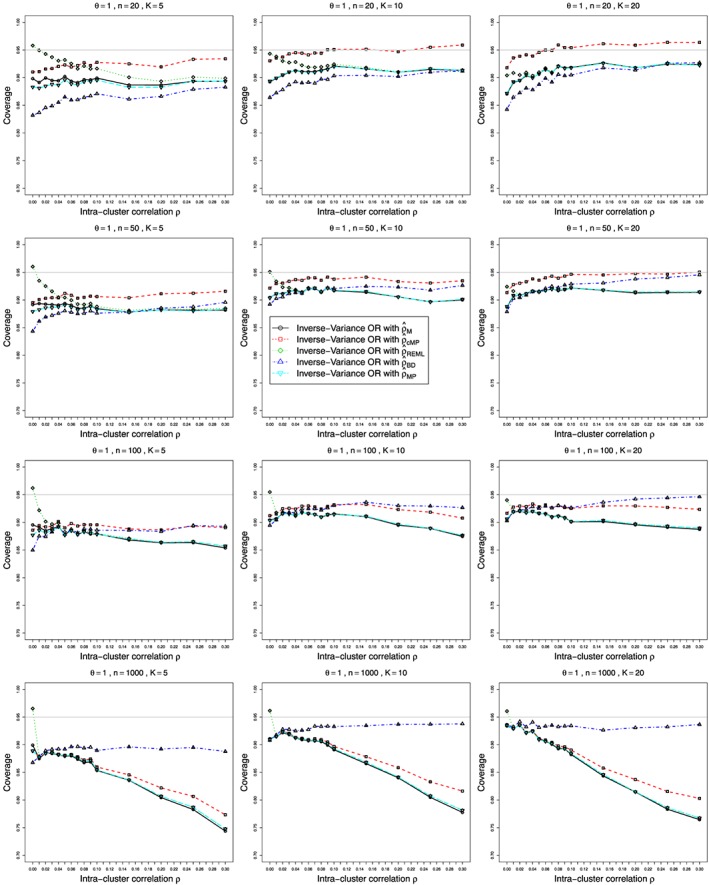
Coverage at the nominal confidence level of 0.95 of the overall odds ratio *ψ* obtained from *K* studies by the inverse‐variance method, for *p*_2*j*_=0.1, *θ*=1 and 0⩽ρ⩽0.3. The inverse‐variance weights use the following estimators of *ρ*: circles – ${\hat{\rho }}_{M}$, squares – Corrected Mandel‐Paule estimator ${\hat{\rho }}_{cMP}$, diamonds – restricted maximum likelihood estimator ${\hat{\rho }}_{REML}$, triangles – Breslow‐Day estimator ${\hat{\rho }}_{BD}$ and reverse‐triangles – Mandel‐Paule estimator ${\hat{\rho }}_{MP}$. Light grey line at 0.95.

**Figure 6:**
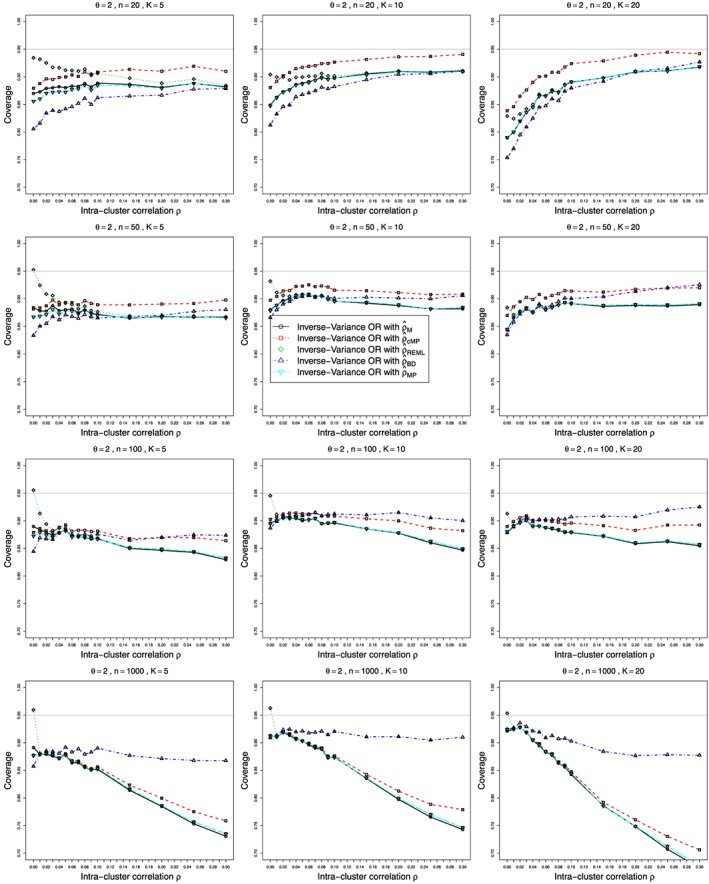
Coverage at the nominal confidence level of 0.95 of the overall odds ratio *ψ* obtained from *K* studies by the inverse‐variance method, for *p*_2*j*_=0.1, *θ*=2 and 0⩽ρ⩽0.3. The inverse‐variance weights use the following estimators of *ρ*: circles – ${\hat{\rho }}_{M}$, squares – Corrected Mandel‐Paule estimator ${\hat{\rho }}_{cMP}$, diamonds – restricted maximum likelihood estimator ${\hat{\rho }}_{REML}$, triangles – Breslow‐Day estimator ${\hat{\rho }}_{BD}$ and reverse‐triangles – Mandel‐Paule estimator ${\hat{\rho }}_{MP}$) Light grey line at 0.95.

Figures 1 and 2 show the bias and coverage of *ρ* estimated by the five methods mentioned earlier for 12 combinations of *K* and *n* for the case of *p*_2*j*_≡0.1 and *θ*=0 and varying values of 0⩽ρ⩽0.3. The bias and coverage of true log‐odds‐ratio *θ* estimated by the inverse‐variance (*θ*_*IV*_) for values of *θ*=0,1,2, are shown in Figures 3, 4, 5, 6, respectively. Similar figures for bias and coverage of *θ* by the modified MH method ( ${\hat{\psi }}_{cMH}$) are given in the Web Appendix, Figure B25–Figure B28. A number of figures show fewer than five lines, because the standard methods including moment based estimation, REML and the Mandel‐Paule method perform similarly. The differences between the standard methods and two new methods, the corrected Mandel‐Paule method and the BD based method, are clearly visible.

#### 6.2.1 Bias and coverage in estimation of intra‐cluster correlation *ρ*

Bias of estimated ICC *ρ* is negative, and its magnitude clearly increases in *ρ*, Figure 1 for *p*_2*j*_=0.1, Figure B1 and Figure B2 in Web Appendix for *p*_2*j*_=0.2 and 0.4. For small numbers of studies *K* combined with small sample sizes ( n⩽50), ${\hat{\rho }}_{cMP}$ estimation appears to be the best option. However, for larger sample sizes ( n⩾100), the BD‐based estimator ${\hat{\rho }}_{BD}$ is the clear winner. Still, its negative bias increases almost linearly with *ρ* and is acceptable only for *ρ*<0.1. Coming to coverage of *ρ* (Figure 2 and Figure B3, Figure B4 in the Web Appendix), once more, the BD‐based estimator appears to be the safest option, apart from the case of very small sample sizes n⩽50, where the gamma‐based approximation appears to provide better coverage for K⩾10. Figure B5 and Figure B6 in the Web Appendix show the bias and coverage in estimation of *ρ* for four values of *θ* and increasing sample size *n*, keeping *ρ*=0.1 fixed. Similar plots of bias and coverage of *ρ* for *p*_2*j*_≡0.2 and *p*_2*j*_≡0.4 are given in Figure B7–Figure B10 in the Web Appendix. BD‐based estimator *ρ*_*BD*_ remains the best estimator of *ρ* for all scenarios for n⩾100, although it acquires a small positive bias when *p*_2*j*_=0.4 and *θ*=3, the case corresponding to *p*_1*j*_=0.93. Similarly, in Figure B11–Figure B16 in the Web Appendix, the results are presented for three values of *p*_2*j*_ and increasing sample size *n*, keeping the value of *θ* fixed. Both bias and coverage improve when the probabilities in both arms are farther from the edges.

#### 6.2.2 Bias in estimation of odds ratio *ψ*

Bias of the estimated OR $\hat{\psi }$ was practically the same regardless of the method used for estimation of ICC *ρ*. This may be due to similarity of sample sizes across studies in our simulations, as the inflation terms (1+(*n*_*i*_−1)*ρ*) in the normalized individual weights ‘almost’ cancel. Without loss of generality, we plotted the results for bias of $\hat{\psi }$ obtained when using the moment estimator ${\hat{\rho }}_{M}$ in Figure 3 for values of log‐odds *θ*=0,1 and 2. There is no bias when *θ*=0, that is, when the probabilities of an event in two arms are the same, but the bias clearly increases with increasing values of *θ* and/or *ρ*. The bias for the inverse‐variance weights is within 10*%* for ρ⩽0.1 or θ⩽2, which would cover the major part of values of these parameters in practice, as *θ*=2 corresponds to an OR of 7.39, and the values of ICC *ρ* are usually small. An explanation of this bias and a possible remedy are provided in Section 6.2.3. Unfortunately, the bias is substantially higher for the modified MH method, especially for small numbers of studies *K* and large values of *ρ* and *n*, and the coverage deteriorates accordingly, see Figure B25–Figure B34 in the Web Appendix, and therefore we do not pursue this estimator further.

#### 6.2.3 Bias of sample log‐odds‐ratio under beta‐binomial model

The bias of a number of popular effect measures used for binary data under REMs is discussed in 45. For log‐odds, it is well known that the sample log‐odds‐ratio

$$\hat{\theta }=log\left(\frac{{\hat{p}}_{1}}{1-{\hat{p}}_{1}}\right)-log\left(\frac{{\hat{p}}_{2}}{1-{\hat{p}}_{2}}\right),$$

where the probabilities of events *p*_1_ and *p*_2_ in treatment and control groups are estimated by sample proportions ${\hat{p}}_{i}=X_{i}/n_{i},i=1,2$, has a bias of order 1/*n* under the FEM *ρ*=0. The standard bias correction studied by Gart *et al.* (1985) adds 1/2 to *X*_*i*_ and to *n*_*i*_−*X*_*i*_, that is, uses ${\tilde{p}}_{i}=(X_{i}+1/2)/(n_{i}+1)$ when estimating the log‐odds to eliminate the 1/*n* bias term at the null model *ρ*=0.

Expanding the log‐odds by Taylor series for a general *ρ*, and taking expectations, (see 45 for details of the derivation)

$$E_{\rho }\left(log\left(\frac{\hat{p}}{1-\hat{p}}\right)\right)=log\left(\frac{p}{1-p}\right)-\frac{\left(1-2p_{})(1+(n-1)\rho \right)}{2np(1-p)}+\cdots \;,$$

where, importantly, the second term includes a bias of order *O*(1) when *ρ*≠0. Therefore, the bias of the sample log‐odds‐ratio $\hat{\theta }$ is

$$bias(\hat{\theta })=-\frac{\left(1-2p_{1})(1+(n_{1}-1)\rho \right)}{2n_{1}p_{1}(1-p_{1})}+\frac{\left(1-2p_{2})(1+(n_{2}-1)\rho \right)}{2n_{2}p_{2}(1-p_{2})}.$$

When log‐odds‐ratio *θ*=0, that is, when the probabilities in the two arms are equal, the biases for sample log‐odds in the two arms cancel out. Thus, the estimate $\hat{\theta }$ is unbiased to order 1/*n*. However, when *θ*≠0 and the probabilities in the two arms are not equal, the sample OR is biased to order *O*(1), and this bias is not ameliorated by the correction. For example, when *p*_1_=0.1 and *p*_2_=0.4, that is, *θ*=−1.791, the main bias term is (−4.444+0.417)*ρ*, increasing linearly with the ICC *ρ*. Figure C35 in the Web Appendix illustrates the quality of this linear approximation to bias. It works well for small values of *ρ*, but the bias increases and higher‐order terms become more important for larger values of *ρ*.

In meta‐analysis with fixed weights, it would be possible to correct the resulting bias of the overall effect measure for small values of *ρ*, but the use of inverse‐variance weights also affects the bias and makes such a correction much more difficult. Luckily, the resulting bias is not very large, as we have seen in Section 6.2.2. We believe that the origin of the higher bias in the corrected MH method is similar, but its consequences are graver.

#### 6.2.4 Coverage of odds‐ratio *ψ*

The method used for estimation of ICC *ρ* is of utmost importance for correct estimation of variance, and therefore the coverage of the OR *ψ*. Plots of coverage for *p*_2_ *j*=0.1 are is presented in Figures 4, 5, 6 for *θ*=0,1 and 2, respectively. Overall, as with bias, the modified Mandel‐Paule estimator ${\hat{\rho }}_{cMP}$ results in the best coverage for small sample sizes up to 50, and *ρ*_*BD*_ provides superior coverage for n⩾100. All other estimators of *ρ* result in inferior coverage, especially for large values of *ρ*. However, there are important differences in coverage when using the best estimators of *ρ* because of differences in true value of the OR. For the small number of studies *K*=5, the coverage is too low for all values of *θ*, but it drifts from about 90*%* to about 87*%* even when the best estimator of *ρ* is used. Starting from *K*=10, the coverage is good for *θ*=0, but becomes lower than nominal when *θ* increases. It is still reasonable, at about 93*%*, for *θ*=1, but reaches 90*%* or even somewhat lower for ${\hat{\rho }}_{BD}$ used with large sample sizes *n*=1000. This is due to the increasing biases in the estimation of *ψ* combined with the ‘improved’ precision for larger sample sizes. Similar plots of coverage for *p*_2*j*_=0.2 and 0.4 when *θ*=0 are given in the Web Appendix (Figure B17, Figure B18). Figure B19 ‐ Figure B24 in the Web Appendix present the bias and coverage when estimating *θ* by ${\hat{\theta }}_{IV}$ for different values of *p*_2*j*_ and increasing sample size *n*, keeping the value of *θ* fixed. These figures clearly show the biases and reduced coverage of OR due to transformation bias discussed in the previous section. Coverage achieved when ${\hat{\rho }}_{BD}$ is used in the weights is superior for moderate to large sample sizes.

## 7 Discussion

In this paper, we developed theory of meta‐analysis of ORs based on the BB model. This model is a natural alternative to the standard REM based on normality of random effects. Of course, other combinations of distributions are possible for meta‐analysis of binomially distributed data. 13 suggest using exact hypergeometric likelihood for individual studies combined with normally distributed random effect for log‐odds. 46 discuss a family of compound binomial distributions obtained by using mixing distributions from the generalized inverse gaussian family of distributions, but these distributions have not been used so far in meta‐analysis.

We have concentrated on the case of two independent BB distributions in two arms of each study. We have proposed two new methods of estimation of the ICC *ρ* in meta‐analysis based on this model. Both our methods work considerably better than other, more traditional methods suggested by 12, and they complement each other by being applicable to meta‐analyses of smaller or larger studies. This model is similar to bivariate binomial‐normal REM for log‐odds‐ratios discussed by [13, p.3056]. The latter model can also incorporate a correlation between the two arms of the same study. However, a similar extension of the BB model is not straightforward.

A version of a bivariate BB distribution was proposed by 47, but this distribution has a strictly positive lower bound for correlation between the marginals, so it does not include the case of independent BB distributions. Moreover, 47 show that ‘independence cannot be obtained as a limit in the parameters without sacrificing the overdispersion’. They also discuss other, previously suggested, versions of a bivariate BB distribution, and possible extensions aimed at resolving this problem, but none are satisfactory. However, a different version that allows a range of correlation values, including zero correlation, was applied to meta‐analysis in 48. A new bivariate beta distribution was recently proposed by 49, but so far it has not been used for mixing binomial distributions.

An important question is how to differentiate between possible REMs, and how robust are the standard REM methods for meta‐analysis of LORs when the BB model is true. A good summary of existing diagnostic methods to differentiate between BB and logistic‐normal model is provided by 50, however this is a difficult task, 51. In Section D of Web Appendix, we provide a simulation study to answer the second question. Briefly, the heterogeneity is best assessed by the moment‐based DerSimonian‐Laird method, in agreement with 52, who do not recommend the use of likelihood based methods for the dependent binary data, because these methods are not robust against model misspecification. The bias of LOR *θ* is very considerable for *θ*=1 and 2, Figure D42, but it is larger than and in the opposite direction to that of *θ* estimated from the true BB model, Figure 3. This bias does not visibly depend on the method of estimation of *τ* ^2^.

We also briefly considered a model with a BB distribution in the treatment arm only. This model is analogous to a version of unconditional random effects logistic regression by 53. In this model, the study‐specific log‐odds of the control groups constitute *K* additional parameters, and this model is not appropriate when K→∞, 13.

We also proposed a variant of the MH method for meta‐analysis of ORs. Unfortunately, in simulations this method was very biased, especially for ORs greater than 1. Elimination of this bias will be pursued elsewhere. The traditional inverse‐variance approach to combining LORs using ICC *ρ* in the weights estimated by one of our methods results in reasonable, although somewhat low coverage for a realistic range of ORs and ICCs. Developed methods and R programs, provided in the Web Appendix, make the BB model a feasible alternative to the standard REM for meta‐analysis of ORs.

## Supporting information

Supporting info item

Supporting info item

Supporting info item

Supporting info item
