# Supplementary material for: Beta‐binomial model for meta‐analysis of odds ratios
Source: Stat Med. 2017 Jan 25;36(11):1715–34. doi: 10.1002/sim.7233 (PMC5434808; doi:10.1002/sim.7233)
Supplement: Supplementary file 1 — Supporting info item [file SIM-36-1715-s001.pdf]

Web Appendix

A. Limits of Mantel-Haenzsel and Inverse-variance odds ratios

A.1. Limit of $\hat{\psi}_{MH}$ when $\rho \to -1/max(a_j)$

The corrected Mantel-Haenzsel odds ratio can be rewritten in the form

$$\hat{\psi}_{cMH} = \frac{\sum_{j=1}^{K} \frac{n_{1j}n_{2j}p_{1j}(1-p_{2j})}{C_{1j}n_{2j}+C_{2j}n_{1j}}}{\sum_{j=1}^{K} \frac{n_{1j}n_{2j}p_{2j}(1-p_{1j})}{C_{1j}n_{2j}+C_{2j}n_{1j}}}$$

When $\rho \to -1/\max(a_j)$, $C_{ij} = 1 - (n_{ij} - 1)/\max(a_j)$. Then the correction factors are

$$C_{1j} = 1 - (n_{1j} - 1)/\max(a_j) \quad \text{and} \quad C_{2j} = 1 - (n_{2j} - 1)/\max(a_j)$$

For balanced studies $n_{1j} = n_{2j} = n_j$, $a_j = n_j - 1$ and $C_{1j} = C_{2j} = C(\rho) = 1 + (n_j - 1)\rho$. For $\rho \to -1/\max(a_j)$

$$C(\rho) = 1 - (n_j - 1)/\max(n_j - 1).$$

The corrected Mantel-Haenzsel odds ratio is

$$\hat{\psi}_{cMH} = \frac{\sum_{n_j=\max(n_j)} \frac{n_{1j}n_{2j}p_{1j}(1-p_{2j})}{2C(\rho)n_j} + \sum_{n_j\neq\max(n_j)} \frac{n_{1j}n_{2j}p_{1j}(1-p_{2j})}{C_{1j}n_{2j}+C_{2j}n_{1j}}}{\sum_{n_j=\max(n_j)} \frac{n_{1j}n_{2j}p_{2j}(1-p_{1j})}{2C(\rho)n_j} + \sum_{n_j\neq\max(n_j)} \frac{n_{1j}n_{2j}p_{1j}(1-p_{2j})}{C_{1j}n_{2j}+C_{2j}n_{1j}}}$$

which is the same as

$$\hat{\psi}_{cMH} = \frac{\sum_{n_j=\max(n_j)} n_{1j}n_{2j}p_{1j}(1-p_{2j}) + 2C(\rho)n_j \sum_{n_j\neq\max(n_j)} \frac{n_{1j}n_{2j}p_{1j}(1-p_{2j})}{C_{1j}n_{2j}+C_{2j}n_{1j}}}{\sum_{n_j=\max(n_j)} n_{1j}n_{2j}p_{2j}(1-p_{1j}) + 2C(\rho)n_j \sum_{n_j\neq\max(n_j)} \frac{n_{1j}n_{2j}p_{1j}(1-p_{2j})}{C_{1j}n_{2j}+C_{2j}n_{1j}}}$$

When $\rho \to -1/\max(a_j)$, $C(\rho) \to 0$, so

$$\hat{\psi}_{cMH} = \frac{\sum_{n_j=\max(n_j)} n_{1j}n_{2j}p_{1j}(1-p_{2j})}{\sum_{n_j=\max(n_j)} n_{1j}n_{2j}p_{2j}(1-p_{1j})} = \frac{\sum_{n_j=\max(n_j)} n_j^2 p_{1j}(1-p_{2j})}{\sum_{n_j=\max(n_j)} n_j^2 p_{2j}(1-p_{1j})}$$

$$\hat{\psi}_{cMH} = \sum_{n_j=\max(n_j)} \frac{p_{1j}(1-p_{2j})}{p_{2j}(1-p_{1j})}$$

A.2. Limit of $\hat{\theta}_{IV}$ when $\rho \to -1/max(a_j)$

The inverse variance odds ratio with weight $w_j(\rho)$ is

$$\hat{\theta}_w = \frac{\sum_{j=1}^{K} w_j(\rho)\hat{\theta}_j}{\sum_{j=1}^{K} w_j(\rho)} = \frac{\sum_{j=1}^{K} \frac{\hat{\theta}_j}{\sigma_j^2(1+a_j\rho)}}{\sum_{j=1}^{K} \frac{1}{\sigma_j^2(1+a_j\rho)}}$$

Similarly to $\hat{\psi}_{MH}$, when $\rho \to -1/\max(a_j)$, $\hat{\theta}_w$ should be subdivided into two components

$$\hat{\theta}_w = \frac{\sum_{n_j=\max(n_j)} \frac{\hat{\theta}_j}{\sigma_j^2(1+a_j\rho)} + \sum_{n_j\neq\max(n_j)} \frac{\hat{\theta}_j}{\sigma_j^2(1+a_j\rho)}}{\sum_{n_j=\max(n_j)} \frac{1}{\sigma_j^2(1+a_j\rho)} + \sum_{n_j\neq\max(n_j)} \frac{1}{\sigma_j^2(1+a_j\rho)}}$$

For balanced studies $n_{1j} = n_{2j} = n_j$, $a_j = n_j - 1$. So, when $\rho \to -1/\max(a_j)$, $1 + a_j\rho \to 0$. Thus,

$$\hat{\theta}_w = \frac{\sum_{n_j=\max(n_j)} \frac{\hat{\theta}_j}{\sigma_j^2} + (1+a_j\rho)|_{n_j=\max(n_j)} \sum_{n_j\neq\max(n_j)} \frac{\hat{\theta}_j}{\sigma_j^2(1+a_j\rho)}}{\sum_{n_j=\max(n_j)} \frac{1}{\sigma_j^2} + (1+a_j\rho)|_{n_j=\max(n_j)} \sum_{n_j\neq\max(n_j)} \frac{1}{\sigma_j^2(1+a_j\rho)}}$$

and

$$\hat{\theta}_w = \frac{\sum_{n_j=\max(n_j)} \frac{\hat{\theta}_j}{\sigma_j^2}}{\sum_{n_j=\max(n_j)} \frac{1}{\sigma_j^2}}$$

Copyright © 2016 John Wiley & Sons, Ltd.

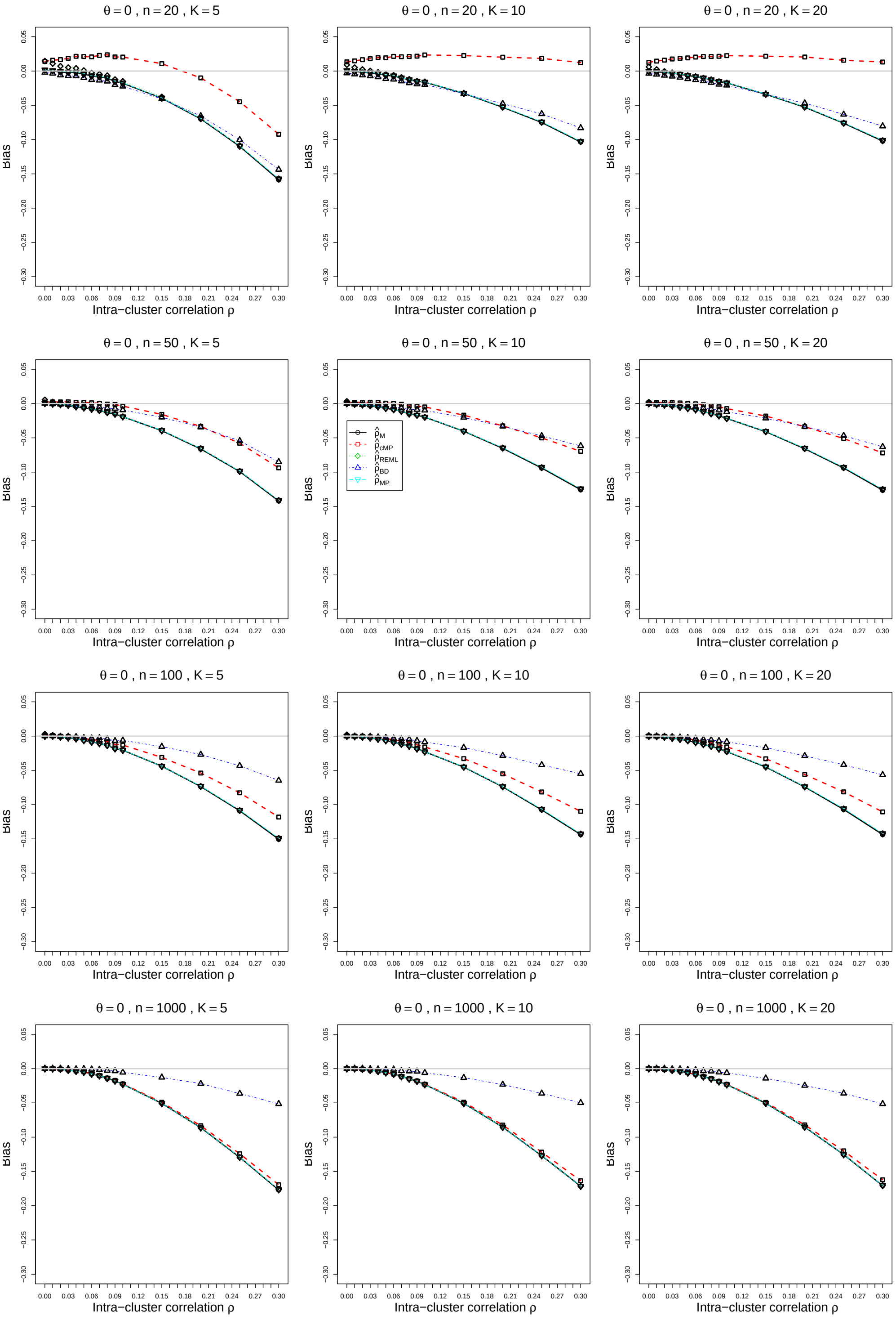

Figure B1. Bias estimated from K studies of the intra-cluster correlation ρ in beta-binomial model for $p_{2j} = 0.2$, $\theta = 0$ and $0 \leq \rho \leq 0.3$ for average sample sizes $n = 20,\ 50,\ 100$ and 1000. The method of estimators for ρ:circles (Moment estimator of ρ - $\hat{\rho}_M$), squares (Corrected Mandel-Paule moment estimator for ρ based on $\Gamma_{r(\rho),\lambda(\rho)}$ distribution - $\hat{\rho}_{cMP}$), diamonds (Restricted maximum likelihood estimator for ρ - $\hat{\rho}_{REML}$), triangles (Breslow-Day estimator for ρ based on χ^2 distribution - $\hat{\rho}_{BD}$) and reverse-triangles (Mandel-Paule estimator of ρ - $\hat{\rho}_{MP}$). Light grey line at 0 for bias.

B. Simulation results

B.1. *Bias and coverage in estimation of intra-cluster correlation* ρ

B.1.1. *Fixed* ρ

Copyright © 2016 John Wiley & Sons, Ltd.

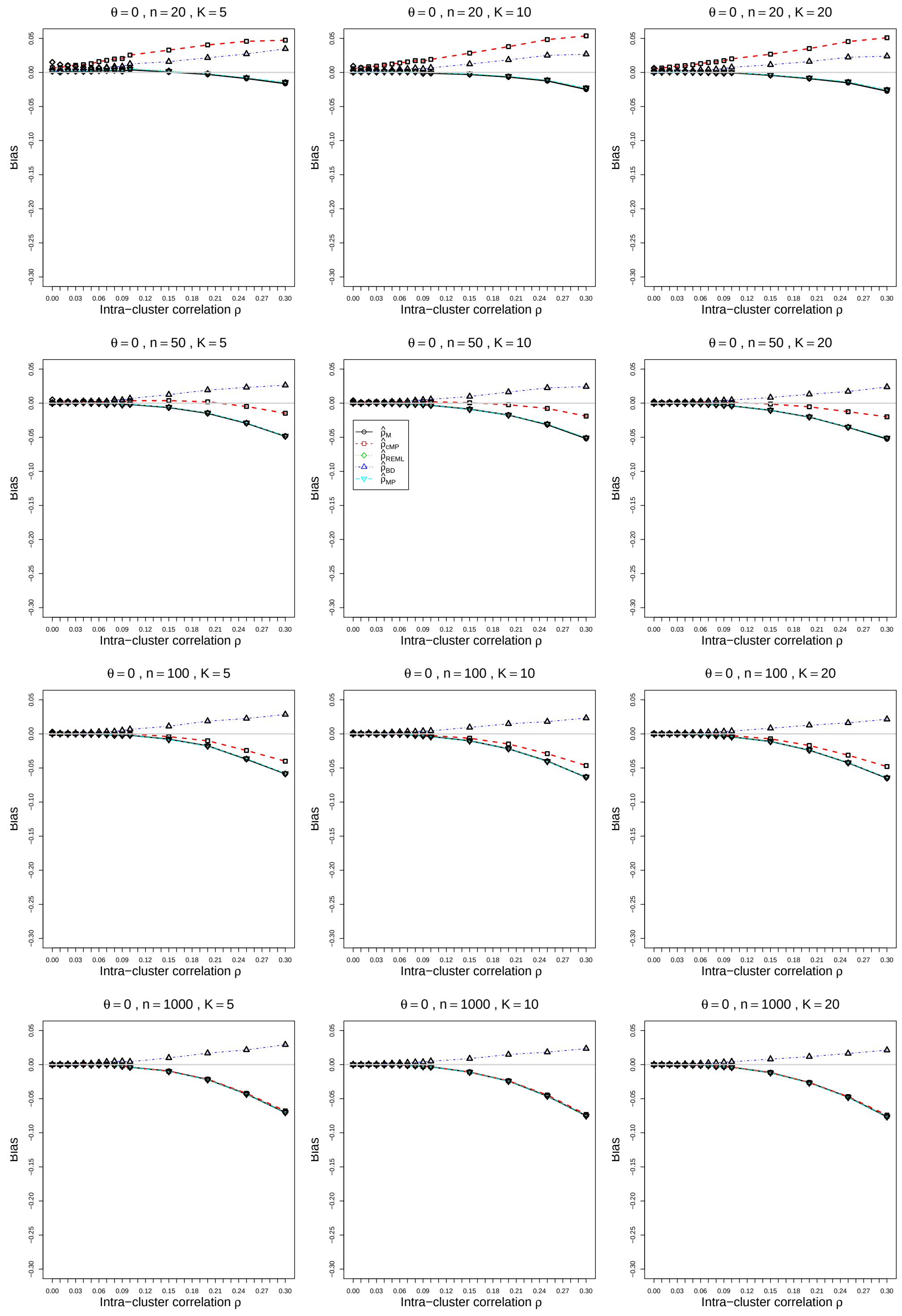

Figure B2. Bias estimated from K studies of the intra-cluster correlation ρ in beta-binomial model for $p_{2j} = 0.4$, $\theta = 0$ and $0 \leq \rho \leq 0.3$ for average sample sizes $n = 20,\ 50,\ 100$ and 1000. The method of estimators for ρ: circles (Moment estimator of ρ - $\hat{\rho}_M$), squares (Corrected Mandel-Paule moment estimator for ρ based on gamma approximation for Q distribution - $\hat{\rho}_{cMP}$), diamonds (Restricted maximum likelihood estimator for ρ - $\hat{\rho}_{REML}$), triangles (Breslow-Day estimator for ρ based on χ^2 distribution - $\hat{\rho}_{BD}$) and reverse-triangles (Mandel-Paule estimator of ρ - $\hat{\rho}_{MP}$). Light grey line at 0 for bias.

Copyright © 2016 John Wiley & Sons, Ltd.

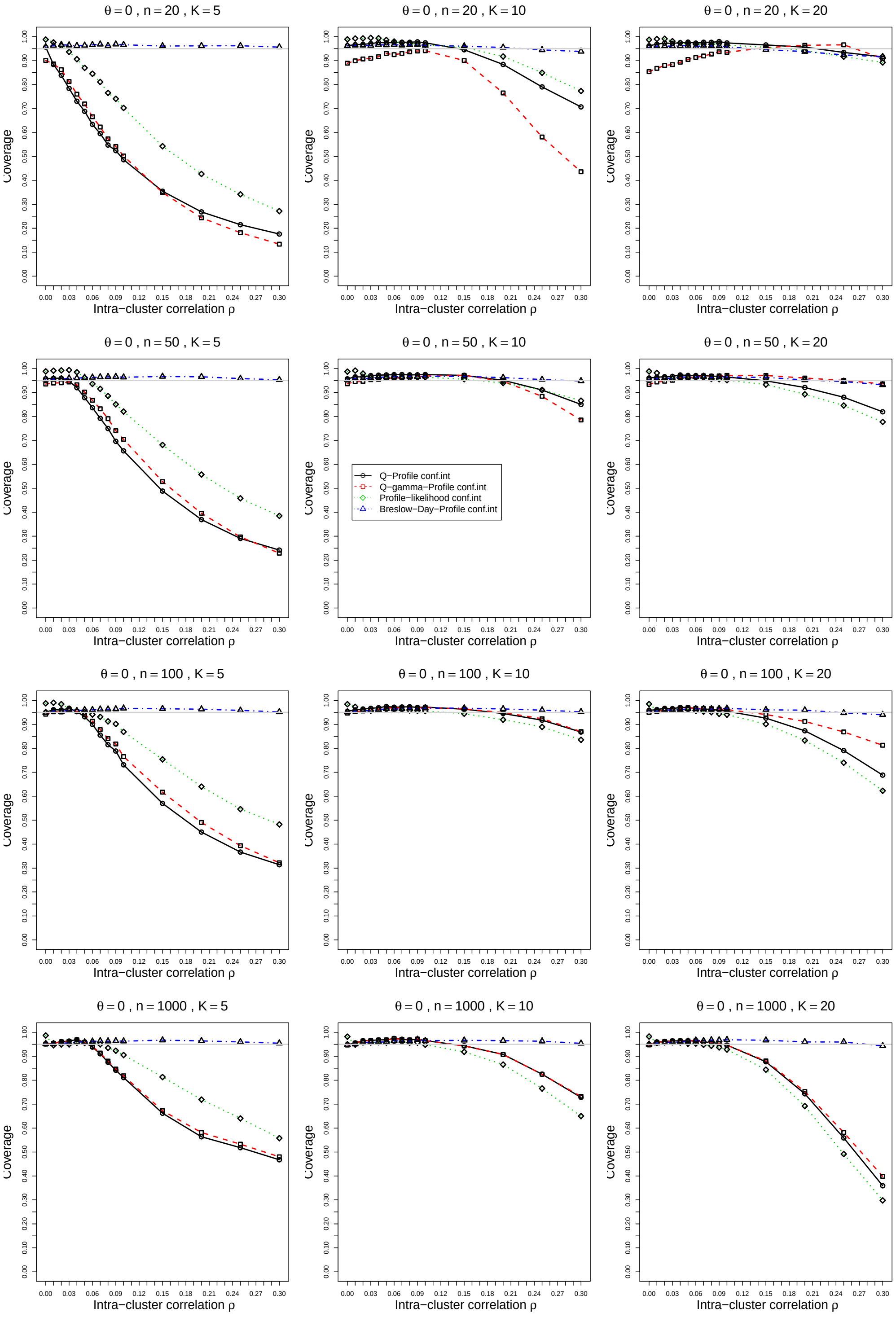

Figure B3. Coverage at the nominal confidence level of 0.95 of the intra-cluster correlation ρ from K studies in beta-binomial model for $p_{2j} = 0.2, \theta = 0$ and $0 \leq \rho \leq 0.3$ for average sample sizes $n = 20,\ 50,\ 100$ and 1000. The method for obtaining the confidence interval are shown as follows: circles (Q-profile confidence interval for ρ based on χ^2 distribution), squares (Q-profile confidence interval for ρ based on $\Gamma_{r(\rho),\lambda(\rho)}$ distribution), diamonds (Profile likelihood confidence intervals) and triangles (Breslow-Day-Profile confidence interval for ρ based on χ^2 distribution). Light grey line at 0.95 for coverage.

 Copyright © 2016 John Wiley & Sons, Ltd.

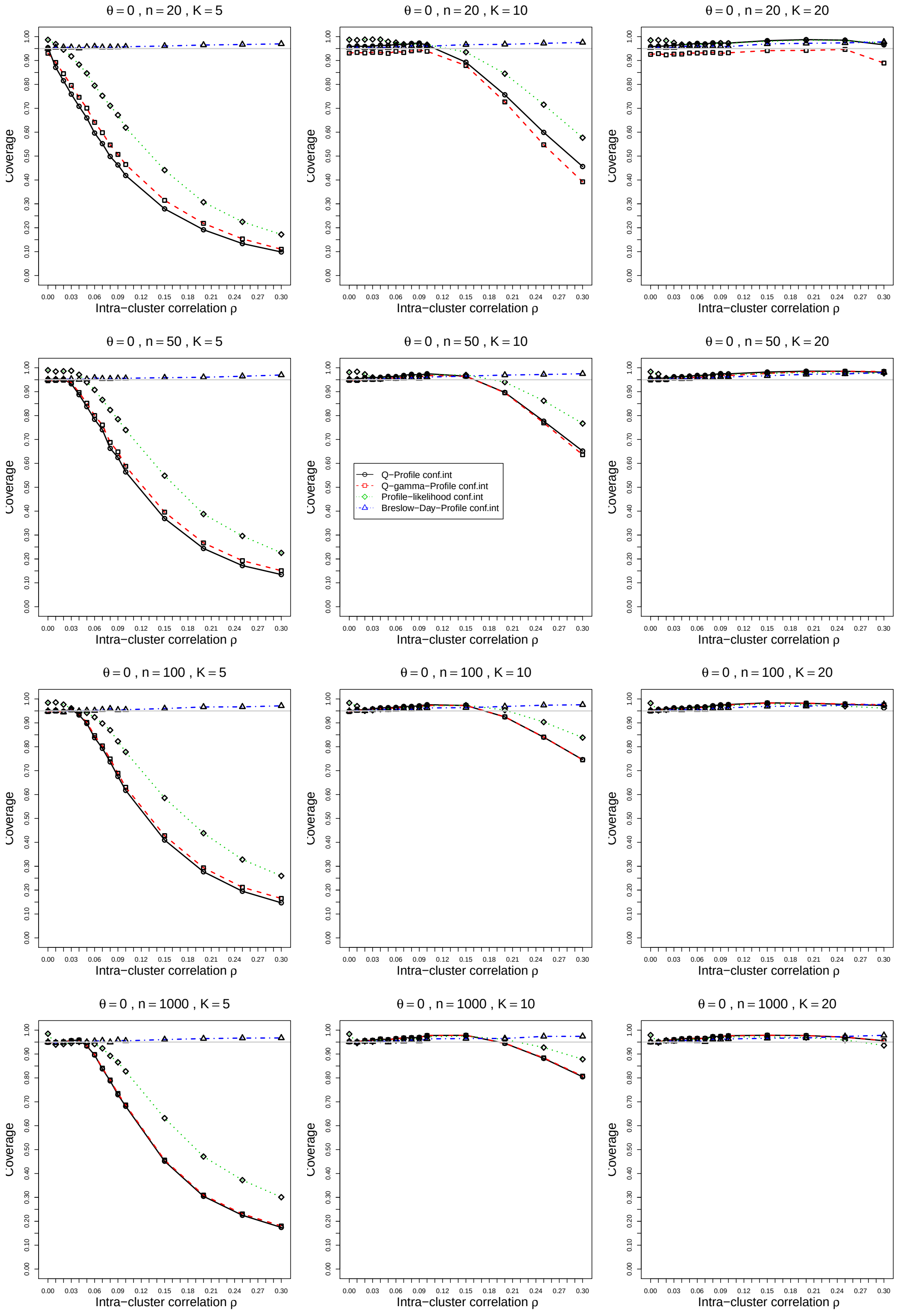

Figure B4. Coverage at the nominal confidence level of 0.95 of the intra-cluster correlation ρ from K studies in beta-binomial model for $p_{2j} = 0.4$, $\theta = 0$ and $0 \leq \rho \leq 0.3$ for average sample sizes $n = 20,\ 50,\ 100$ and 1000. The method for obtaining the confidence interval are shown as follows: circles (Q-profile confidence interval for ρ based on χ^2 distribution), squares (Q-profile confidence interval for ρ based on $\Gamma_{r(\rho),\lambda(\rho)}$ distribution), diamonds (Profile likelihood confidence intervals) and triangles (Breslow-Day-Profile confidence interval for ρ based on χ^2 distribution) Light grey line at 0.95 for coverage.

Copyright © 2016 John Wiley & Sons, Ltd.

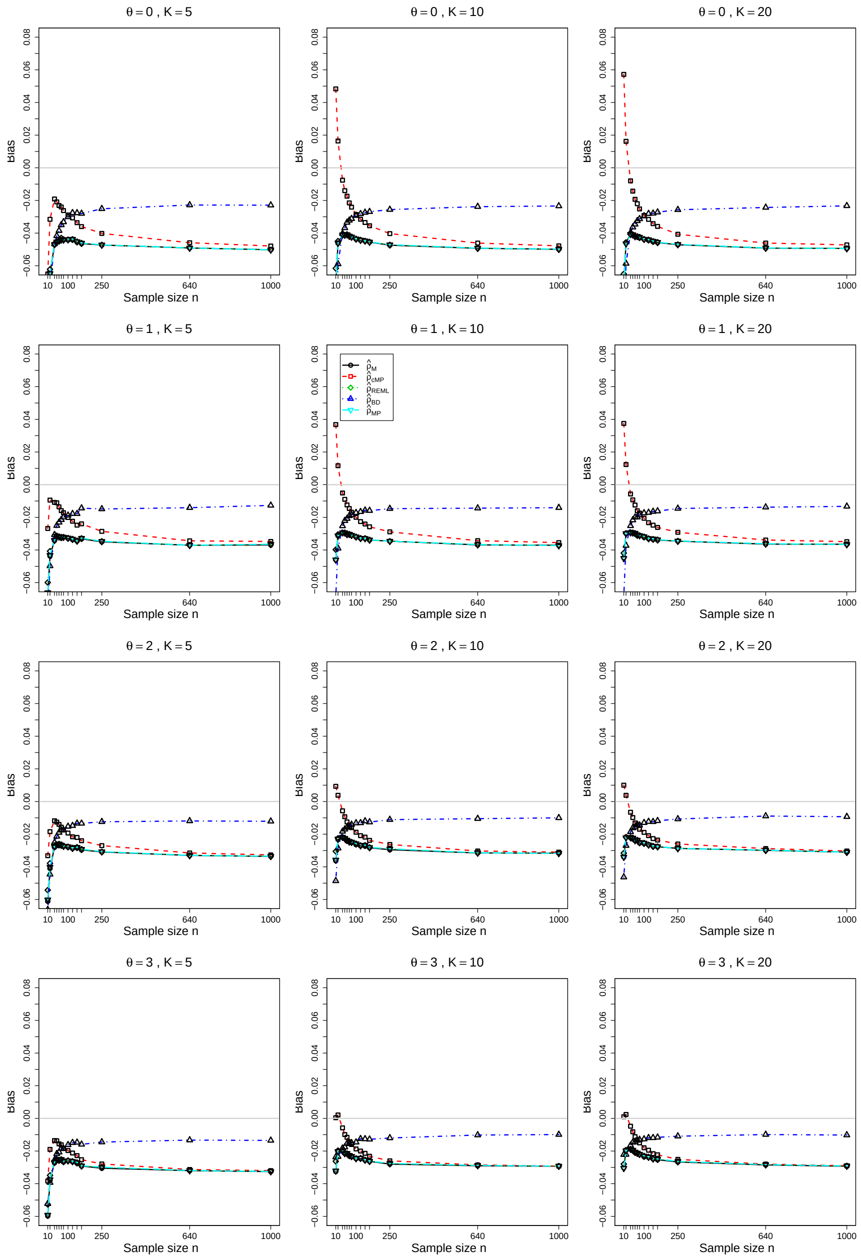

Figure B5. Bias estimated from K studies of the intra-cluster correlation ρ in beta-binomial model for $p_{2j} = 0.1$, $0 \leq \theta \leq 3$, $\rho = 0.1$ and $10 \leq n \leq 1000$. The method of estimators for ρ: circles (Moment estimator of ρ - $\hat{\rho}_M$), squares (Corrected Mandel-Paule moment estimator for ρ based on $\Gamma_{r(\rho),\lambda(\rho)}$ distribution - $\hat{\rho}_{cMP}$), diamonds (Restricted maximum likelihood estimator for ρ - $\hat{\rho}_{REML}$), triangles (Breslow-Day estimator for ρ based on χ^2 distribution - $\hat{\rho}_{BD}$) and reverse-triangles (Mandel-Paule estimator of ρ - $\hat{\rho}_{MP}$). Light grey line at 0 for bias.

B.1.2. Fixed p_{2j}

Copyright © 2016 John Wiley & Sons, Ltd.

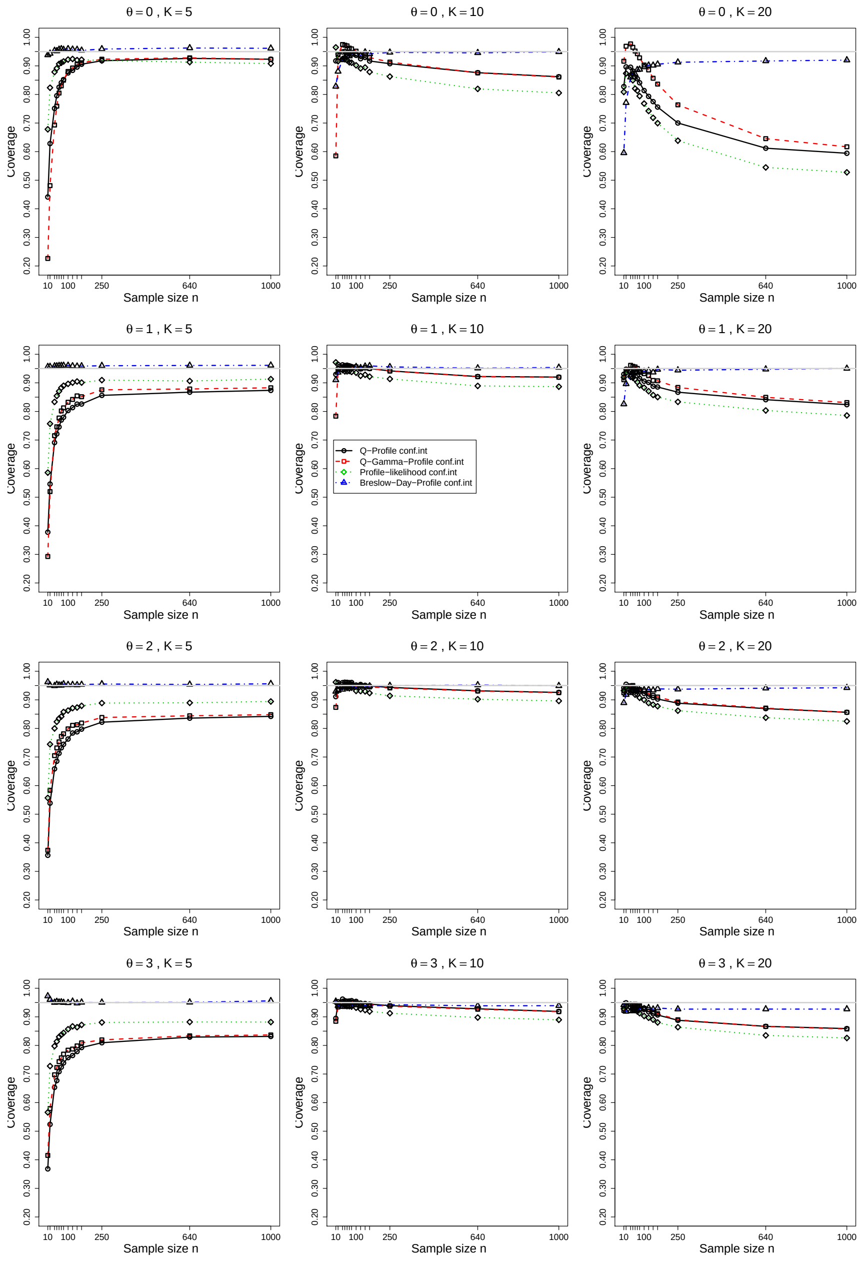

Figure B6. Coverage at the nominal confidence level of 0.95 of the intra-cluster correlation ρ from K studies in beta-binomial model for $p_{2j} = 0.1$, $0 \leq \theta \leq 3$, $\rho = 0.1$ and $10 \leq n \leq 1000$. The method for obtaining the confidence interval are shown as follows: circles (Q-profile confidence interval for ρ based on χ^2 distribution), squares (Q-profile confidence interval for ρ based on $\Gamma_{r(\rho),\lambda(\rho)}$ distribution), diamonds (Profile likelihood confidence intervals) and triangles (Breslow-Day-Profile confidence interval for ρ based on χ^2 distribution). Light grey line at 0.95 for coverage.

Copyright © 2016 John Wiley & Sons, Ltd.

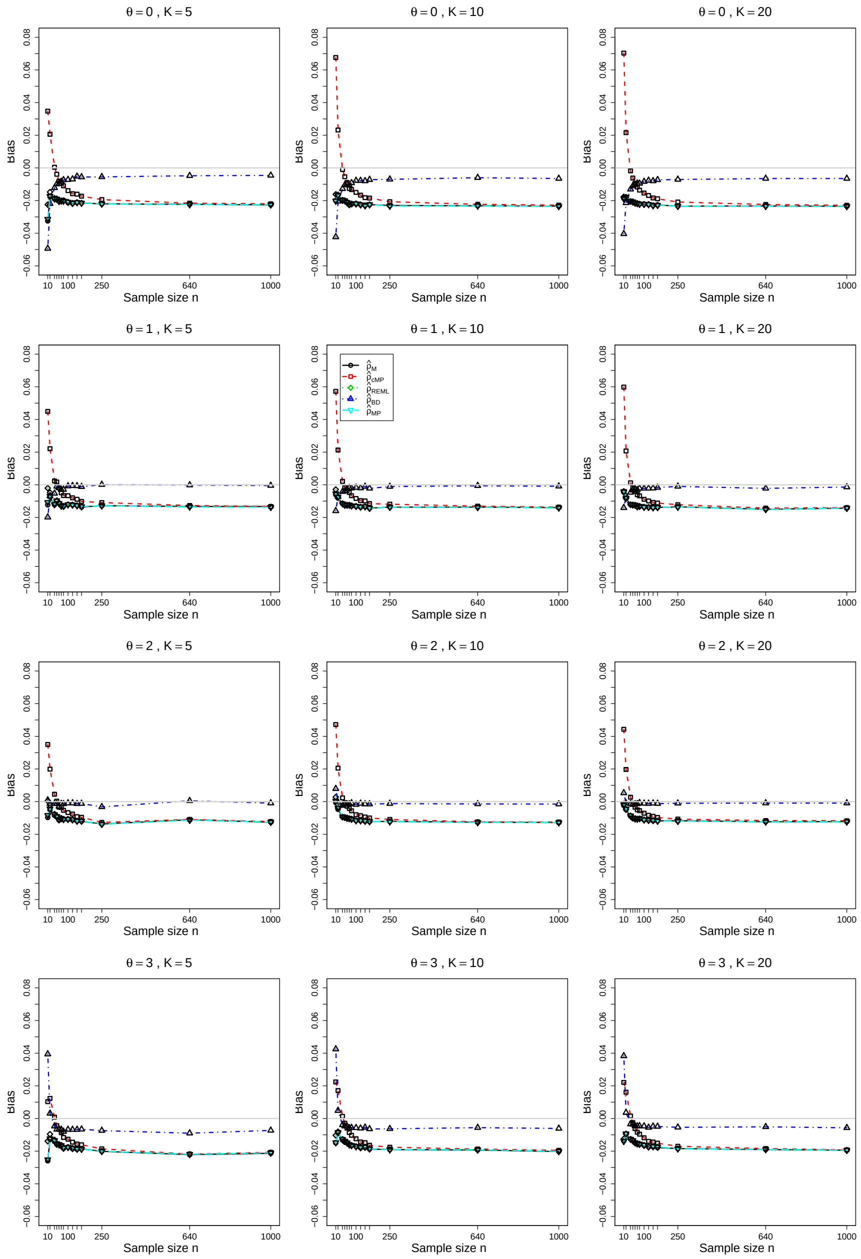

Figure B7. Bias estimated from K studies of the intra-cluster correlation ρ in beta-binomial model for $p_{2j} = 0.2$, $0 \leq \theta \leq 3$, $\rho = 0.1$ and $10 \leq n \leq 1000$. The method of estimators for ρ: circles (Moment estimator of ρ - $\hat{\rho}_M$), squares (Corrected Mandel-Paule moment estimator for ρ based on $\Gamma_{r(\rho),\lambda(\rho)}$ distribution - $\hat{\rho}_{cMP}$), diamonds (Restricted maximum likelihood estimator for ρ - $\hat{\rho}_{REML}$), triangles (Breslow-Day estimator for ρ based on χ^2 distribution - $\hat{\rho}_{BD}$) and reverse-triangles (Mandel-Paule estimator of ρ - $\hat{\rho}_{MP}$). Light grey line at 0 for bias.

 Copyright © 2016 John Wiley & Sons, Ltd.

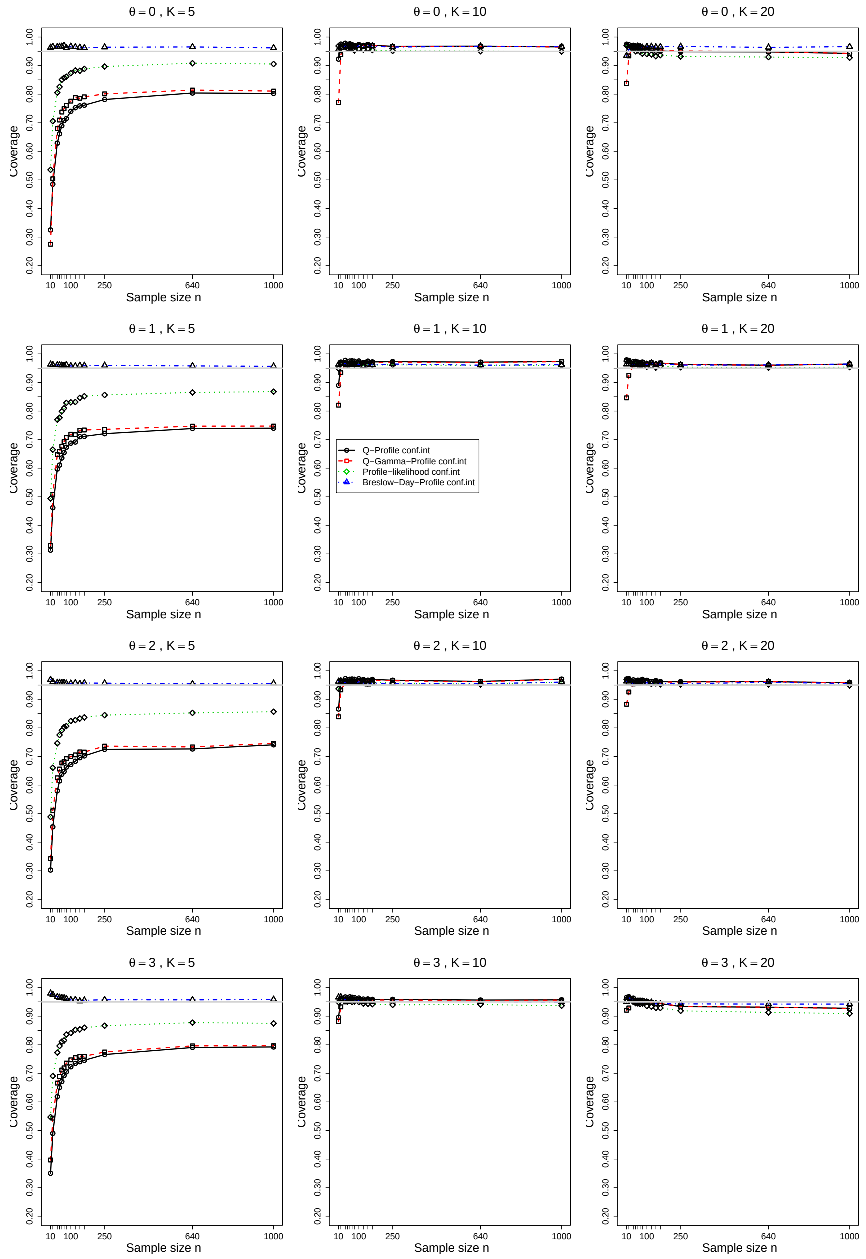

Figure B8. Coverage at the nominal confidence level of 0.95 of the intra-cluster correlation ρ from K studies in beta-binomial model for $p_{2j} = 0.2$, $0 \leq \theta \leq 3$, $\rho = 0.1$ and $10 \leq n \leq 1000$. The method for obtaining the confidence interval are shown as follows: circles (Q-profile confidence interval for ρ based on χ^2 distribution), squares (Q-profile confidence interval for ρ based on $\Gamma_{r(\rho),\lambda(\rho)}$ distribution), diamonds (Profile likelihood confidence intervals) and triangles (Breslow-Day-Profile confidence interval for ρ based on χ^2 distribution). Light grey line at 0.95 for coverage.

Copyright © 2016 John Wiley & Sons, Ltd.

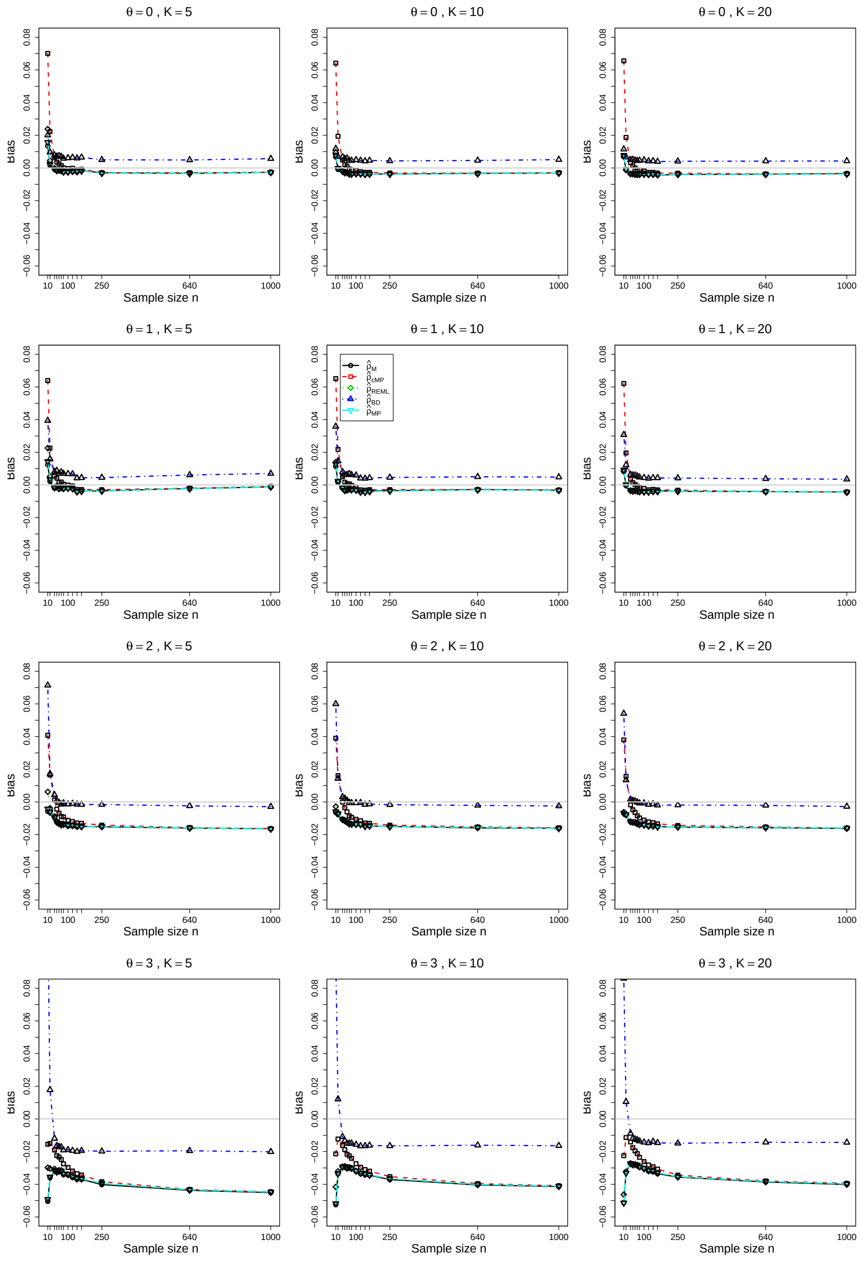

Figure B9. Bias estimated from K studies of the intra-cluster correlation ρ in beta-binomial model for $p_{2j} = 0.4$, $0 \leq \theta \leq 3$, $\rho = 0.1$ and $10 \leq n \leq 1000$. The method of estimators for ρ: circles (Moment estimator of ρ - $\hat{\rho}_M$), squares (Corrected Mandel-Paule moment estimator for ρ based on $\Gamma_{r(\rho),\lambda(\rho)}$ distribution - $\hat{\rho}_{cMP}$), diamonds (Restricted maximum likelihood estimator for ρ - $\hat{\rho}_{REML}$), triangles (Breslow-Day estimator for ρ based on χ^2 distribution - $\hat{\rho}_{BD}$) and reverse-triangles (Mandel-Paule estimator of ρ - $\hat{\rho}_{MP}$). Light grey line at 0 for bias.

 Copyright © 2016 John Wiley & Sons, Ltd.

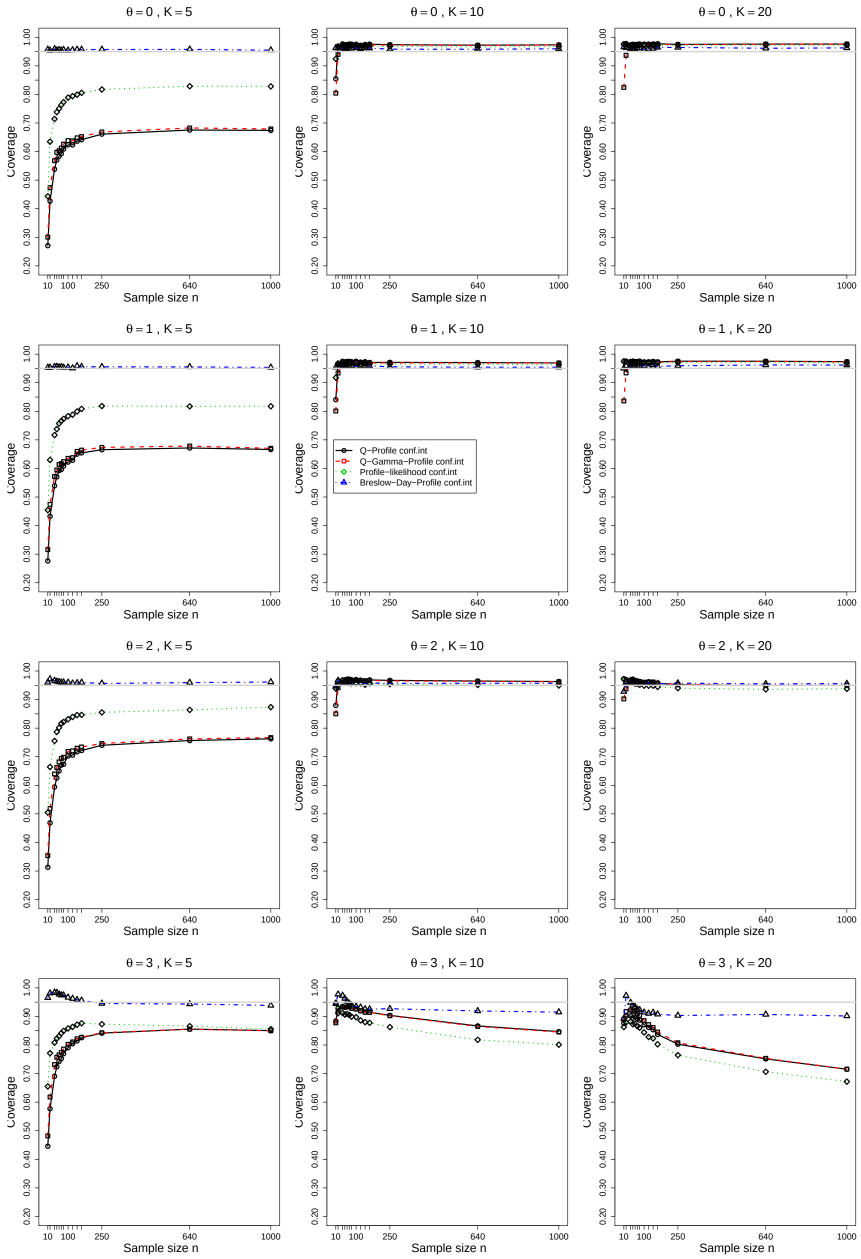

Figure B10. Coverage at the nominal confidence level of 0.95 of the intra-cluster correlation ρ from K studies in beta-binomial model for $p_{2j} = 0.4$, $0 \le \theta \le 3$, $\rho = 0.1$ and $10 \le n \le 1000$. The method for obtaining the confidence interval are shown as follows: circles (Q-profile confidence interval for ρ based on χ^2 distribution), squares (Q-profile confidence interval for ρ based on $\Gamma_{r(\rho),\lambda(\rho)}$ distribution), diamonds (Profile likelihood confidence intervals) and triangles (Breslow-Day-Profile confidence interval for ρ based on χ^2 distribution). Light grey line at 0.95 for coverage.

Copyright © 2016 John Wiley & Sons, Ltd.

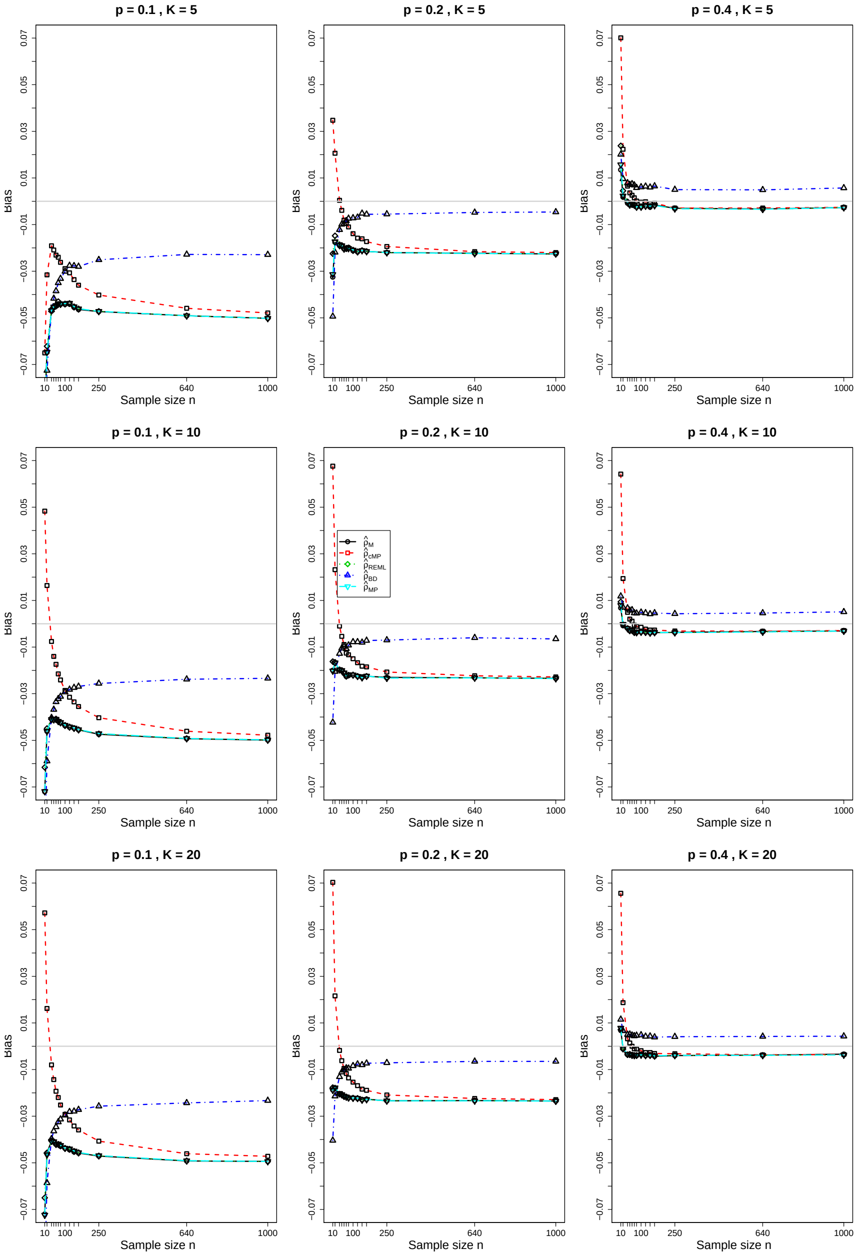

Figure B11. Bias estimated from K studies of the intra-cluster correlation ρ in beta-binomial model for $0.1 \leq p_{2j} \leq 0.4$, $\theta = 0$, $\rho = 0.1$ and $10 \leq n \leq 1000$. The method of estimators for ρ: circles (Moment estimator of ρ - $\hat{\rho}_M$), squares (Corrected Mandel-Paule moment estimator for ρ based on $\Gamma_{r(\rho),\lambda(\rho)}$ distribution - $\hat{\rho}_{cMP}$), diamonds (Restricted maximum likelihood estimator for ρ - $\hat{\rho}_{REML}$), triangles (Breslow-Day estimator for ρ based on χ^2 distribution - $\hat{\rho}_{BD}$) and reverse-triangles (Mandel-Paule estimator of ρ - $\hat{\rho}_{MP}$). Light grey line at 0 for bias.

B.1.3. Fixed θ

 Copyright © 2016 John Wiley & Sons, Ltd.

Statistics
in Medicine

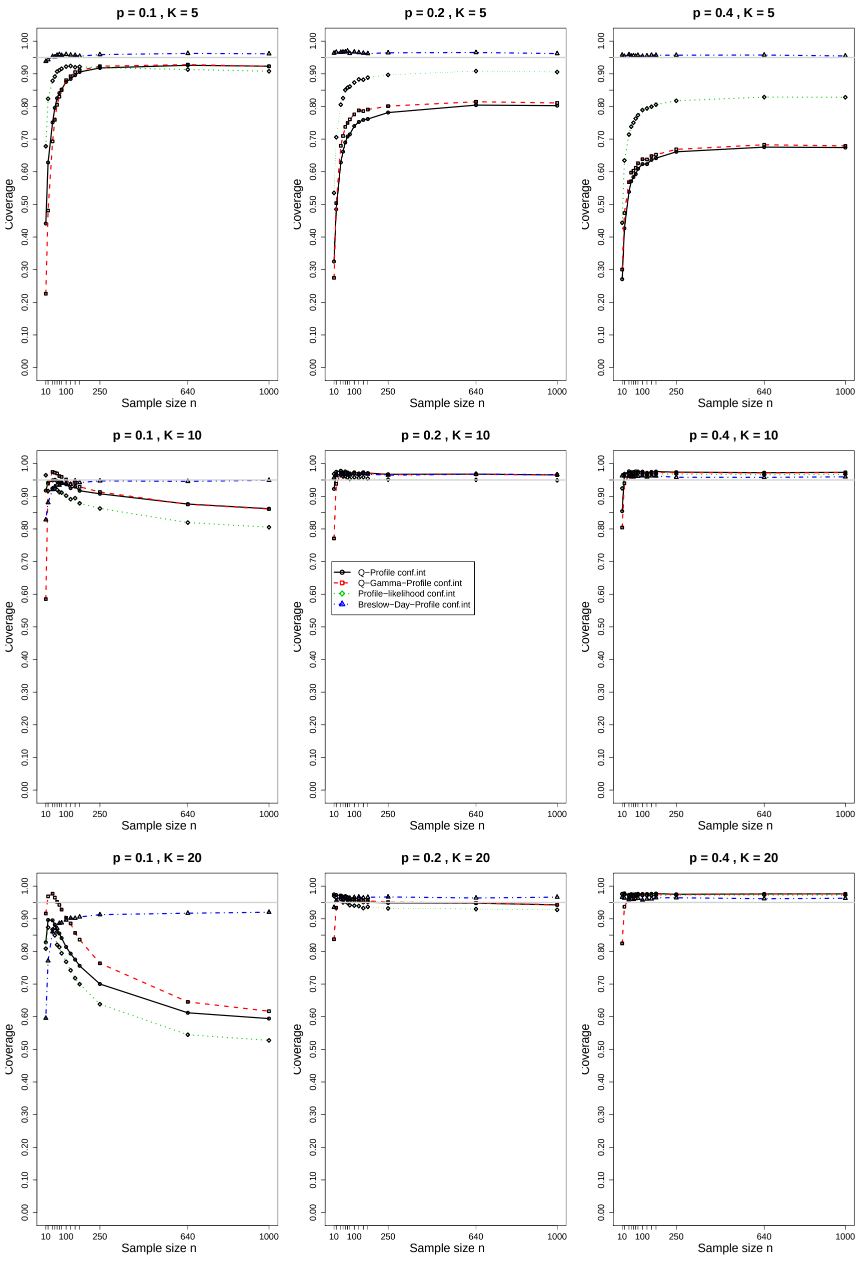

Figure B12. Coverage at the nominal confidence level of 0.95 of the intra-cluster correlation ρ from K studies in beta-binomial model for $0.1 \leq p_{2j} \leq 0.4$, $\theta = 0$, $\rho = 0.1$ and $10 \leq n \leq 1000$. The method for obtaining the confidence interval are shown as follows: circles (Q-profile confidence interval for ρ based on χ^2 distribution), squares (Q-profile confidence interval for ρ based on $\Gamma_{r(\rho),\lambda(\rho)}$ distribution), diamonds (Profile likelihood confidence intervals) and triangles (Breslow-Day-Profile confidence interval for ρ based on χ^2 distribution). Light grey line at 0.95 for coverage.

Copyright © 2016 John Wiley & Sons, Ltd.

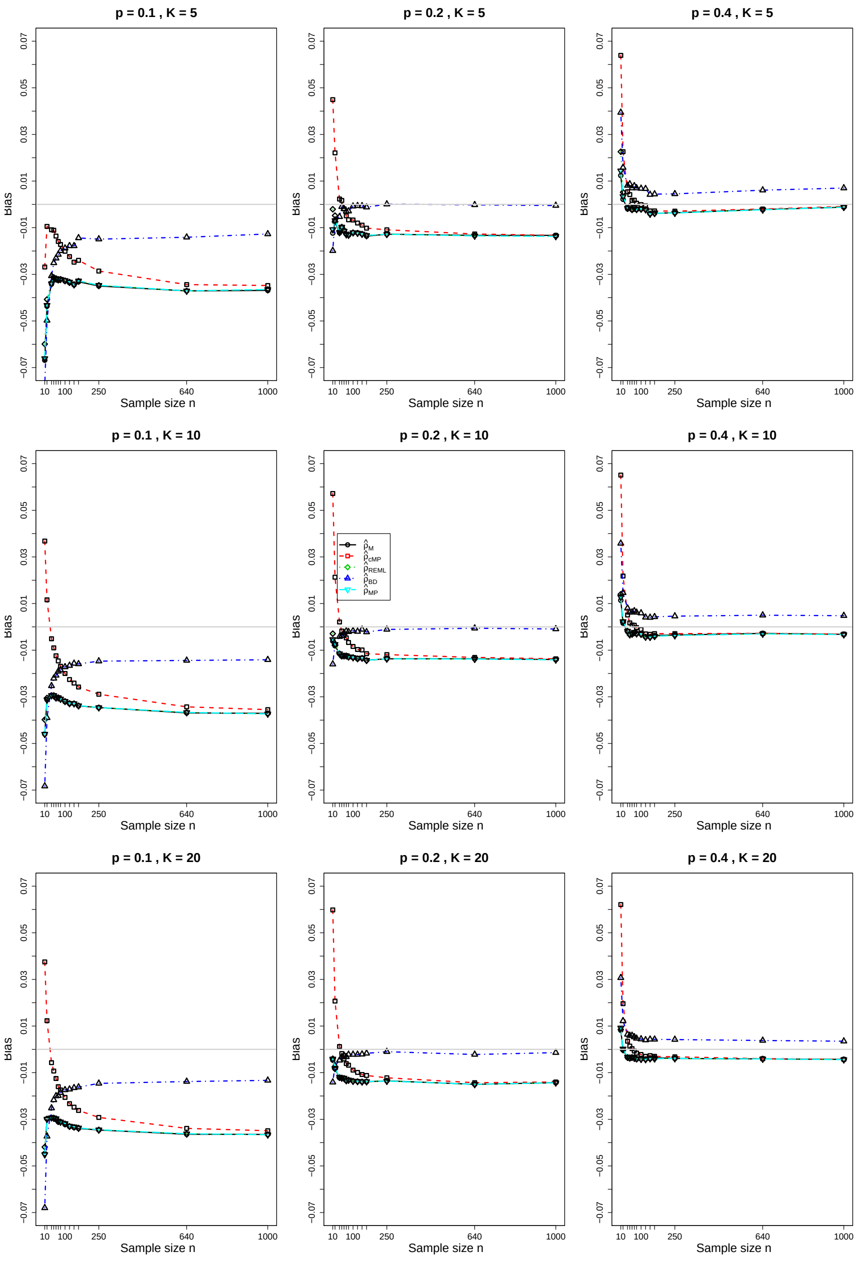

Figure B13. Bias estimated from K studies of the intra-cluster correlation ρ in beta-binomial model for $0.1 \leq p_{2j} \leq 0.4$, $\theta = 1$, $\rho = 0.1$ and $10 \leq n \leq 1000$. The method of estimators for ρ: circles (Moment estimator of ρ - $\hat{\rho}_M$), squares (Corrected Mandel-Paule moment estimator for ρ based on $\Gamma_{r(\rho),\lambda(\rho)}$ distribution - $\hat{\rho}_{cMP}$), diamonds (Restricted maximum likelihood estimator for ρ - $\hat{\rho}_{REML}$), triangles (Breslow-Day estimator for ρ based on χ^2 distribution - $\hat{\rho}_{BD}$) and reverse-triangles (Mandel-Paule estimator of ρ - $\hat{\rho}_{MP}$). Light grey line at 0 for bias.

 Copyright © 2016 John Wiley & Sons, Ltd.

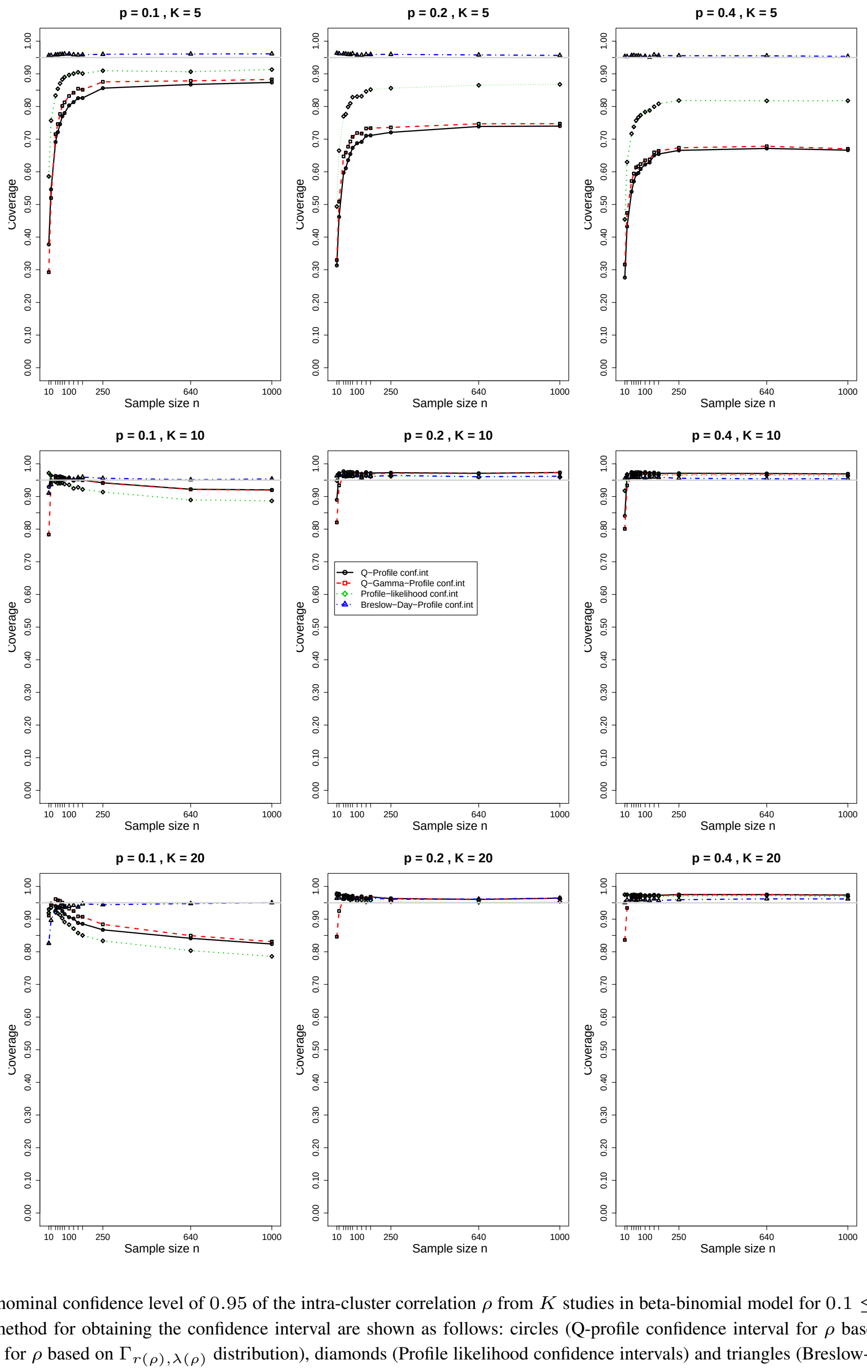

Figure B14. Coverage at the nominal confidence level of 0.95 of the intra-cluster correlation ρ from K studies in beta-binomial model for $0.1 \le p_{2j} \le 0.4$, $\theta = 1$, $\rho = 0.1$ and $10 \le n \le 1000$. The method for obtaining the confidence interval are shown as follows: circles (Q-profile confidence interval for ρ based on χ^2 distribution), squares (Q-profile confidence interval for ρ based on $\Gamma_{r(\rho),\lambda(\rho)}$ distribution), diamonds (Profile likelihood confidence intervals) and triangles (Breslow-Day-Profile confidence interval for ρ based on χ^2 distribution). Light grey line at 0.95 for coverage.

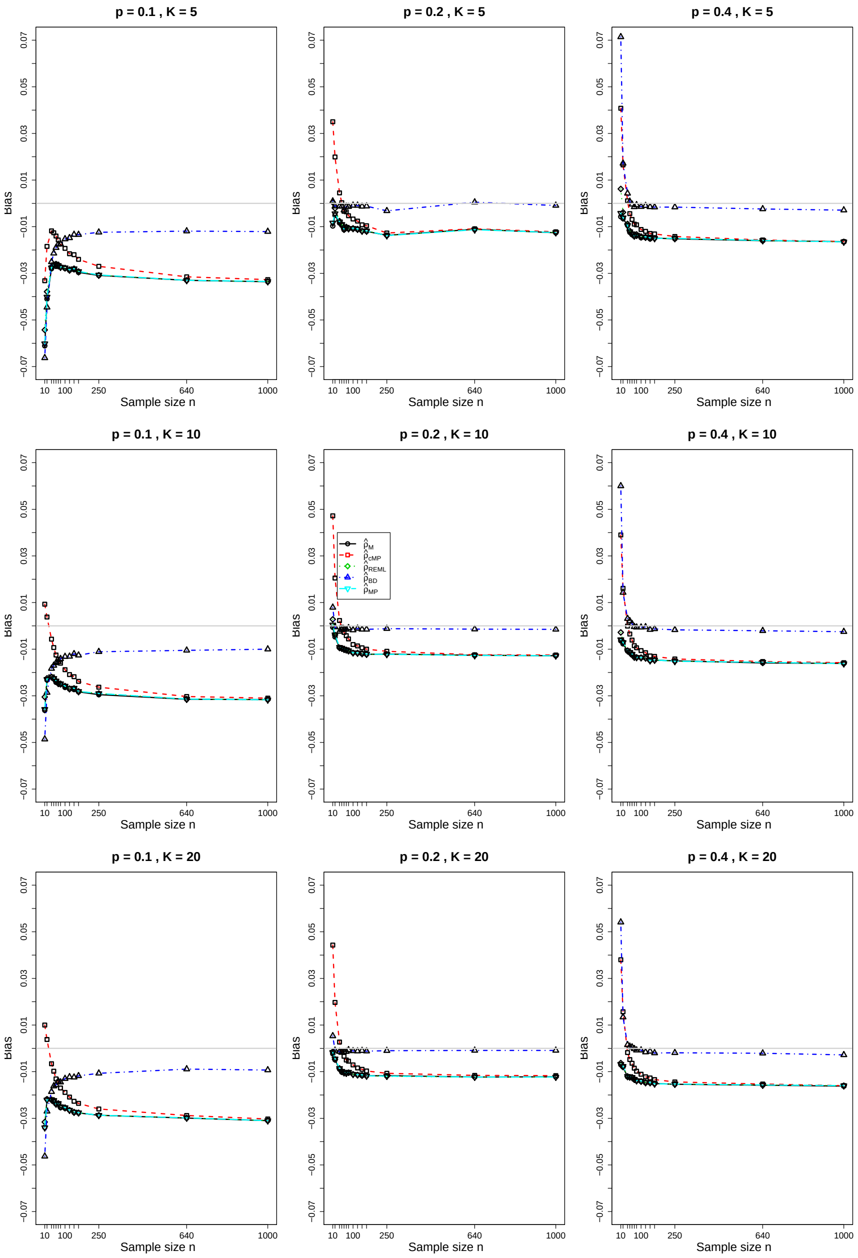

Figure B15. Bias estimated from K studies of the intra-cluster correlation ρ in beta-binomial model for $0.1 \leq p_{2j} \leq 0.4$, $\theta = 2$, $\rho = 0.1$ and $10 \leq n \leq 1000$. The method of estimators for ρ: circles (Moment estimator of ρ - $\hat{\rho}_M$), squares (Corrected Mandel-Paule moment estimator for ρ based on $\Gamma_{r(\rho),\lambda(\rho)}$ distribution - $\hat{\rho}_{cMP}$), diamonds (Restricted maximum likelihood estimator for ρ - $\hat{\rho}_{REML}$), triangles (Breslow-Day estimator for ρ based on χ^2 distribution - $\hat{\rho}_{BD}$) and reverse-triangles (Mandel-Paule estimator of ρ - $\hat{\rho}_{MP}$). Light grey line at 0 for bias.

 Copyright © 2016 John Wiley & Sons, Ltd.

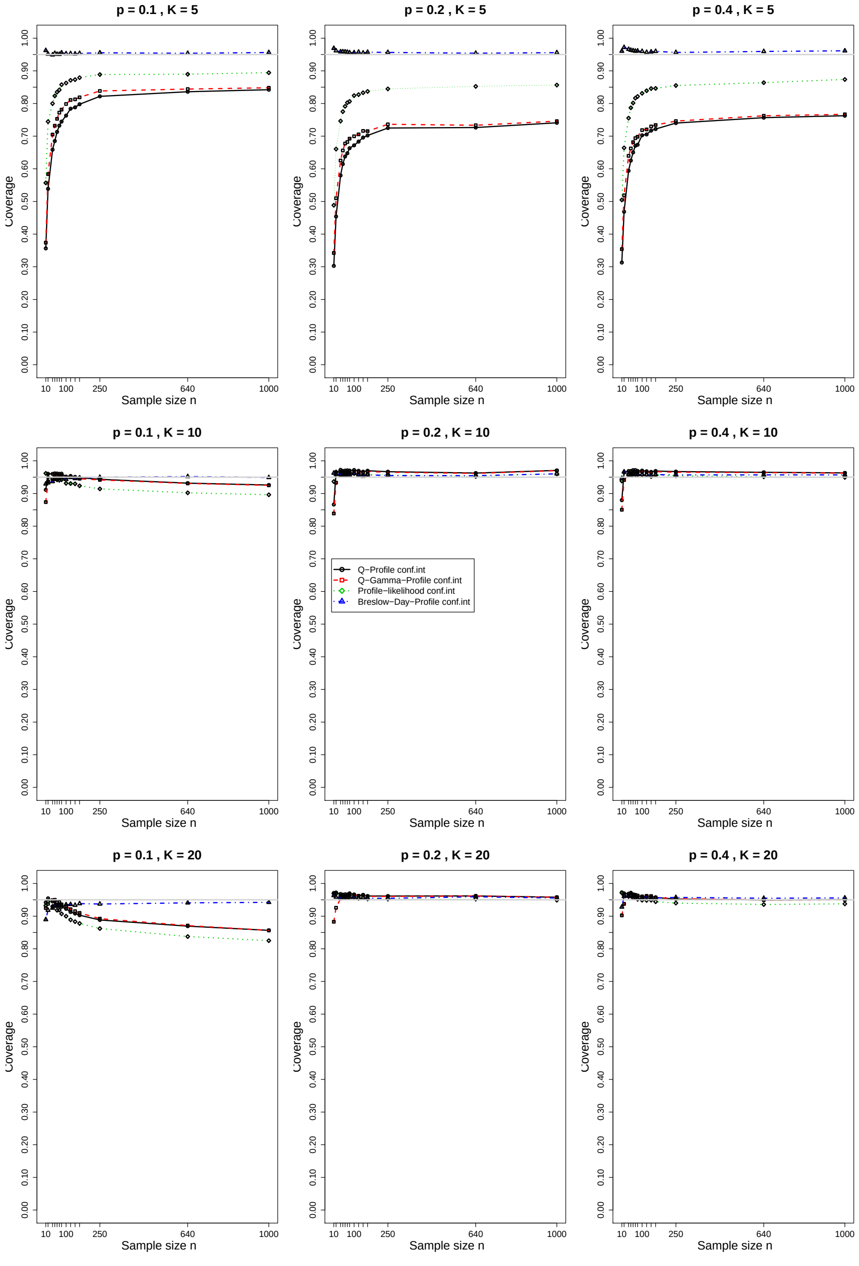

Figure B16. Coverage at the nominal confidence level of 0.95 of the intra-cluster correlation ρ from K studies in beta-binomial model for $0.1 \leq p_{2j} \leq 0.4$, $\theta = 2$, $\rho = 0.1$ and $10 \leq n \leq 1000$. The method for obtaining the confidence interval are shown as follows: circles (Q-profile confidence interval for ρ based on χ^2 distribution), squares (Q-profile confidence interval for ρ based on $\Gamma_{r(\rho),\lambda(\rho)}$ distribution), diamonds (Profile likelihood confidence intervals) and triangles (Breslow-Day-Profile confidence interval for ρ based on χ^2 distribution). Light grey line at 0.95 for coverage.

Copyright © 2016 John Wiley & Sons, Ltd.

Statistics
in Medicine

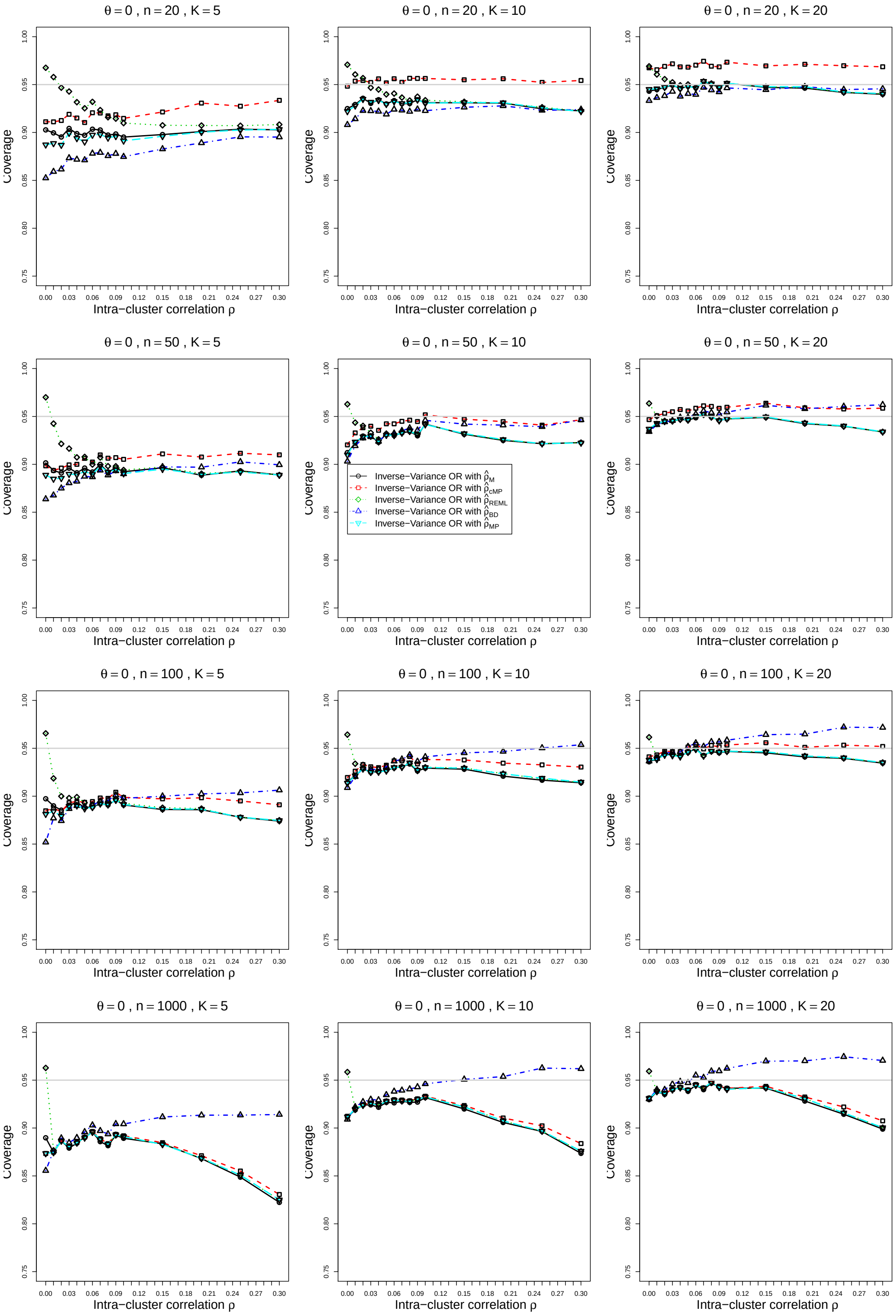

Figure B17. Coverage at the nominal confidence level of 0.95 of the inverse-variance overall effect measure θ_{IV} from K studies in beta-binomial model for $p_{2j} = 0.2$, $\theta = 0$ and $0 \leq \rho \leq 0.3$. The inverse-variance weights use the estimators of ρ: circles (Moment estimator of ρ - $\hat{\rho}_M$), squares (Corrected Mandel-Paule moment estimator for ρ based on gamma approximation for Q distribution - $\hat{\rho}_{cMP}$), diamonds (Restricted maximum likelihood estimator for ρ - $\hat{\rho}_{REML}$), triangles (Breslow-Day estimator for ρ based on χ^2 distribution - $\hat{\rho}_{BD}$) and reverse-triangles (Mandel-Paule estimator of ρ - $\hat{\rho}_{MP}$). Light grey line at 0.95 for coverage.

B.2. Bias and coverage in estimation of overall effect measure θ_{IV}

 Copyright © 2016 John Wiley & Sons, Ltd.

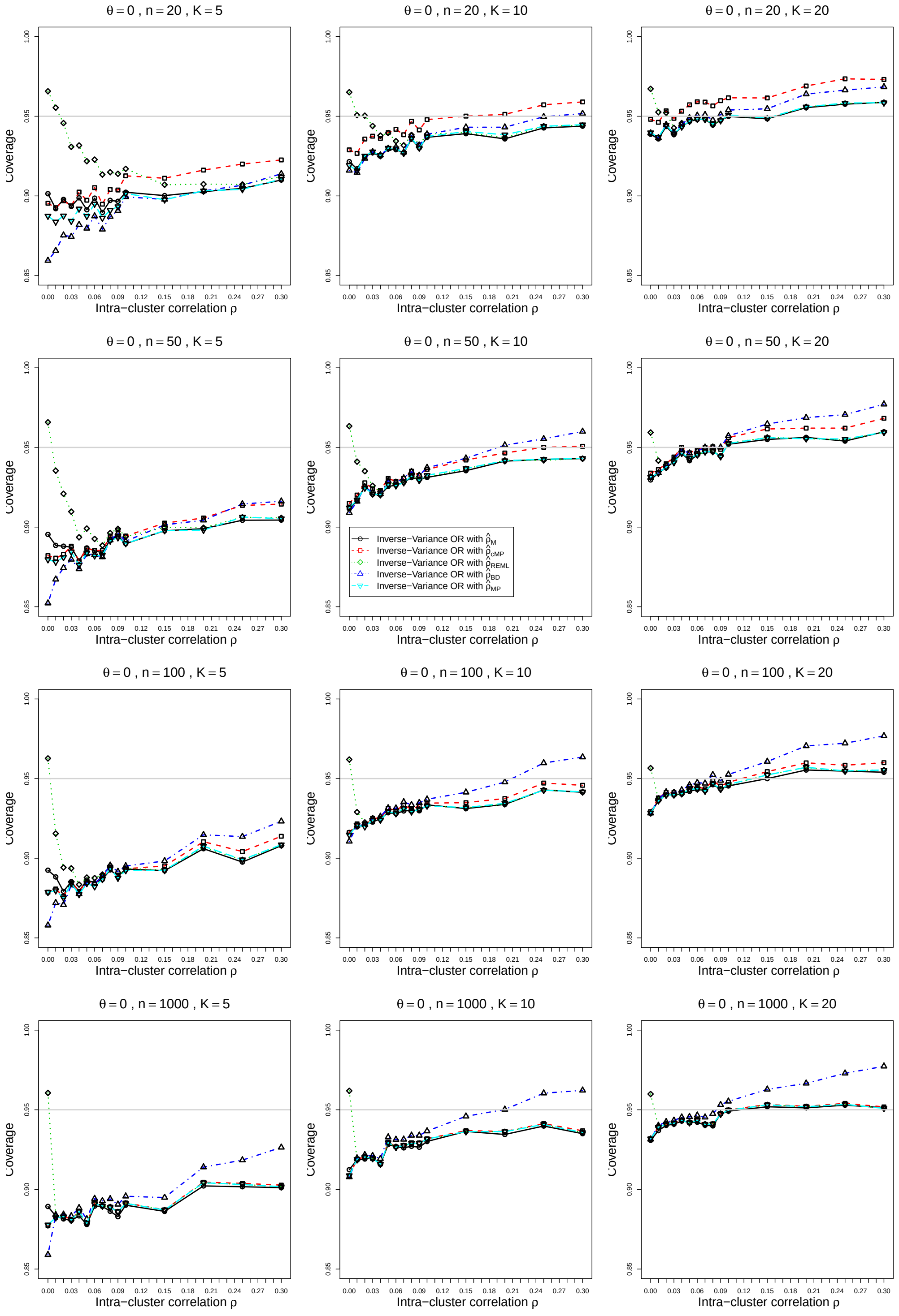

Figure B18. Coverage at the nominal confidence level of 0.95 of the inverse-variance overall effect measure θ_{IV} from K studies in beta-binomial model for $p_{2j} = 0.4$, $\theta = 0$ and $0 \leq \rho \leq 0.3$. The inverse-variance weights use the estimators of ρ: circles (Moment estimator of ρ - $\hat{\rho}_M$), squares (Corrected Mandel-Paule moment estimator for ρ based on gamma approximation for Q distribution - $\hat{\rho}_{cMP}$), diamonds (Restricted maximum likelihood estimator for ρ - $\hat{\rho}_{REML}$), triangles (Breslow-Day estimator for ρ based on χ^2 distribution - $\hat{\rho}_{BD}$) and reverse-triangles (Mandel-Paule estimator of ρ - $\hat{\rho}_{MP}$). Light grey line at 0.95 for coverage.

Copyright © 2016 John Wiley & Sons, Ltd.

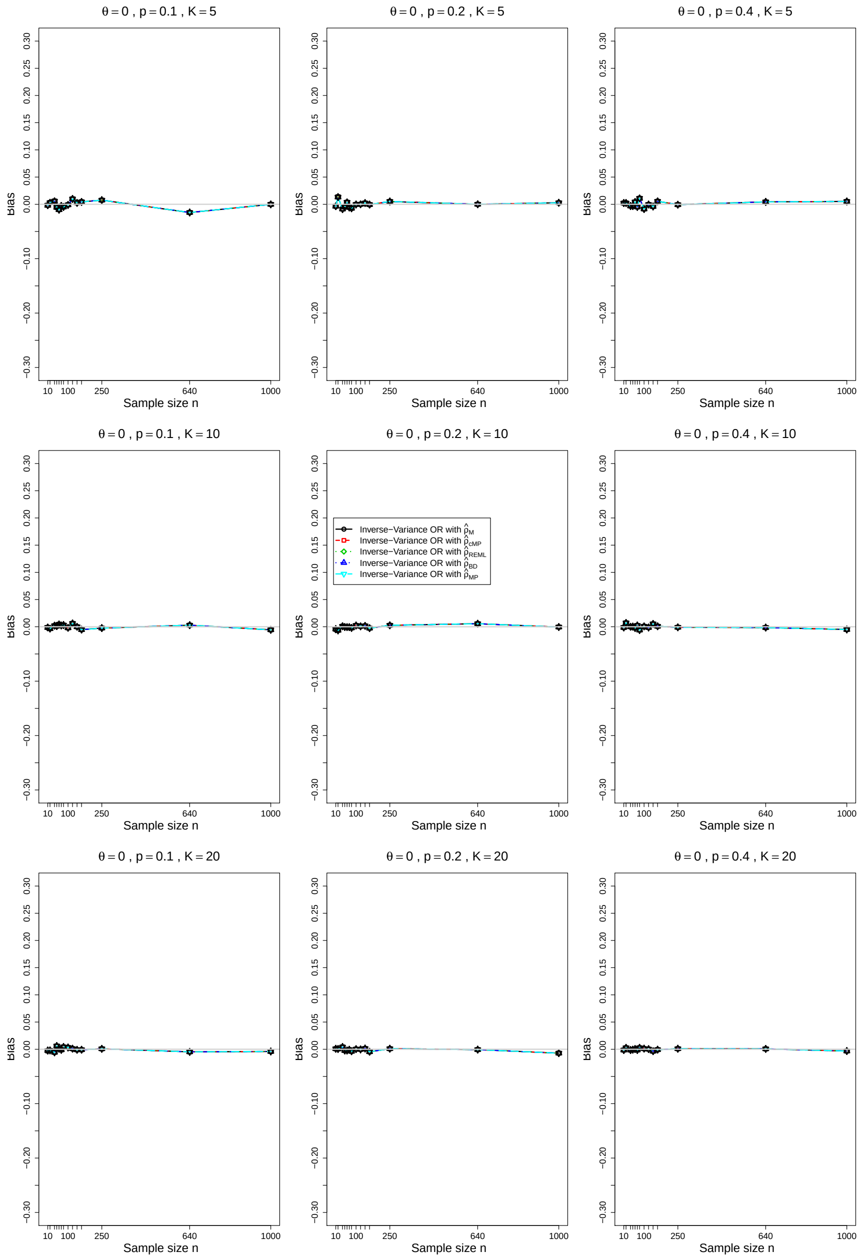

Figure B19. Bias of the inverse-variance overall effect measure ψ_{IV} from K studies in beta-binomial model for $0.1 \leq p_{2j} \leq 0.4$, $\theta = 0$, $\rho = 0.1$ and $10 \leq n \leq 1000$. The inverse-variance weights use the estimators of ρ: circles (Moment estimator of ρ - $\hat{\rho}_M$), squares (Corrected Mandel-Paule moment estimator for ρ based on gamma approximation for Q distribution - $\hat{\rho}_{cMP}$), diamonds (Restricted maximum likelihood estimator for ρ - $\hat{\rho}_{REML}$), triangles (Breslow-Day estimator for ρ based on χ^2 distribution - $\hat{\rho}_{BD}$) and reverse-triangles (Mandel-Paule estimator of ρ - $\hat{\rho}_{MP}$). Light grey line at 0 for bias.

B.3. Fixed θ

 Copyright © 2016 John Wiley & Sons, Ltd.

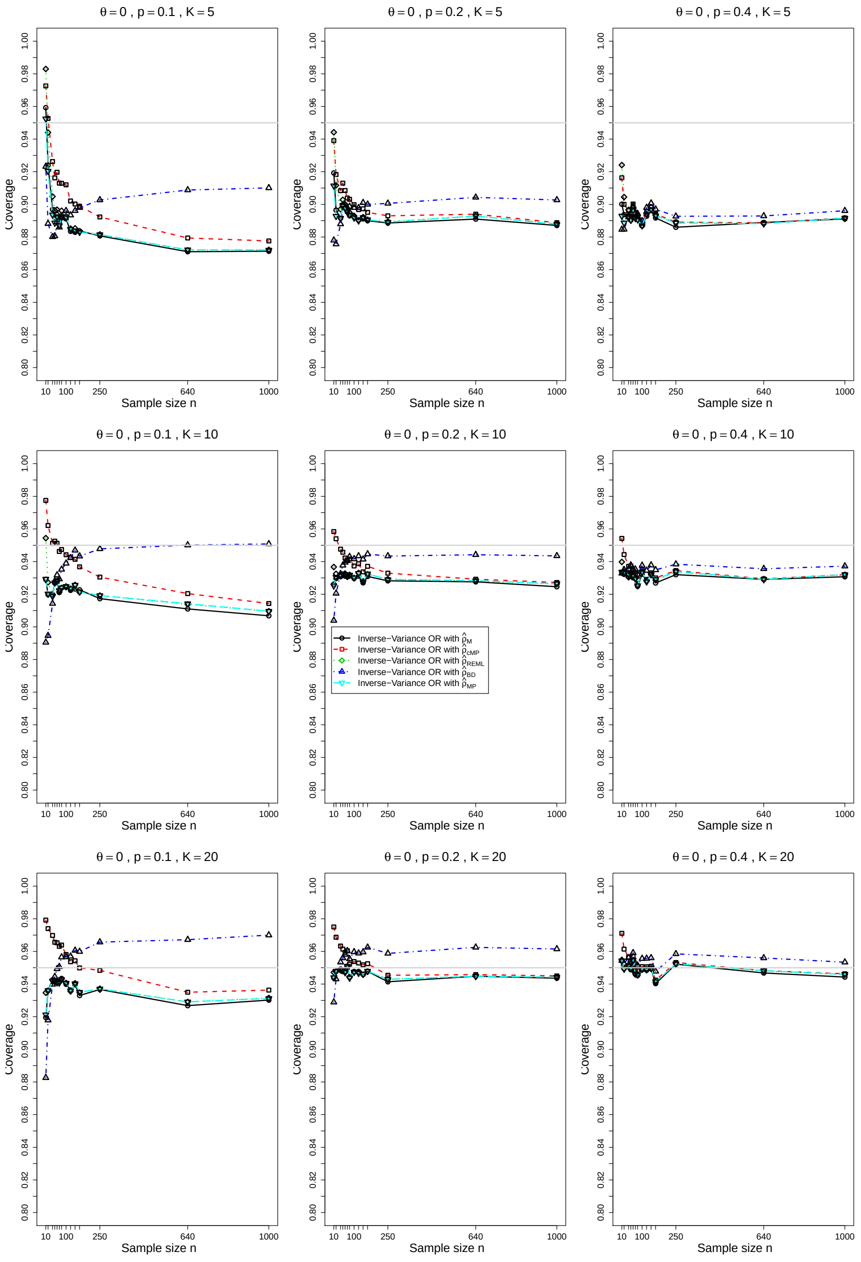

Figure B20. Coverage at the nominal confidence level of 0.95 of the Inverse-Variance overall effect measure θ_{IV} from K studies in beta-binomial model for $0.1 \leq p_{2j} \leq 0.4$, $\theta = 0$, $\rho = 0.1$ and $10 \leq n \leq 1000$. The weights of the Mandel-Haenzsel odds ratio use the estimators of ρ: circles (Moment estimator of ρ - $\hat{\rho}_M$), squares (Corrected Mandel-Paule moment estimator for ρ based on gamma approximation for Q distribution - $\hat{\rho}_{cMP}$), diamonds (Restricted maximum likelihood estimator for ρ - $\hat{\rho}_{REML}$), triangles (Breslow-Day estimator for ρ based on χ^2 distribution - $\hat{\rho}_{BD}$) and reverse-triangles (Mandel-Paule estimator of ρ - $\hat{\rho}_{MP}$). Light grey line at 0.95 for coverage.

Copyright © 2016 John Wiley & Sons, Ltd.

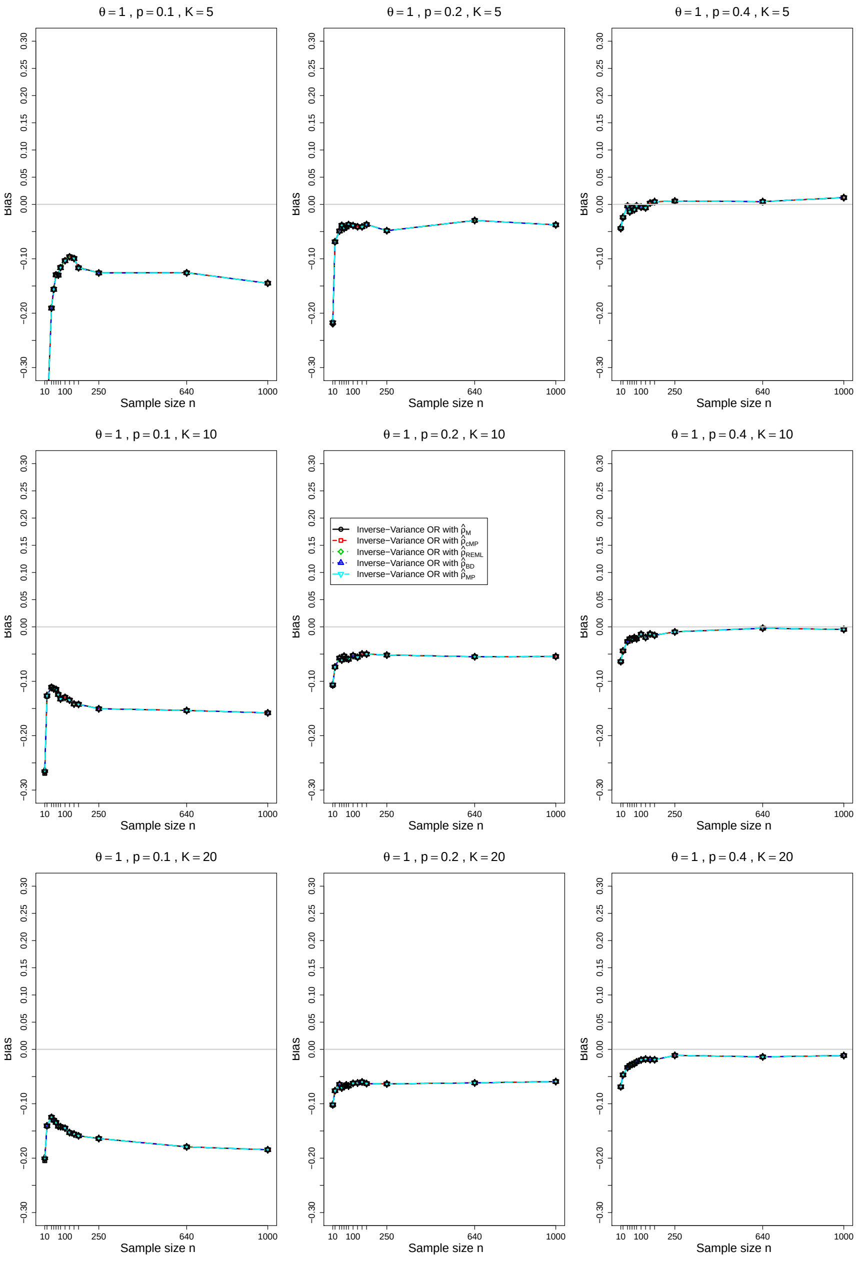

Figure B21. Bias of the inverse-variance overall effect measure ψ_{IV} from K studies in beta-binomial model for $0.1 \leq p_{2j} \leq 0.4$, $\theta = 1$, $\rho = 0.1$ and $10 \leq n \leq 1000$. The inverse-variance weights use the estimators of ρ: circles (Moment estimator of ρ - $\hat{\rho}_M$), squares (Corrected Mandel-Paule moment estimator for ρ based on gamma approximation for Q distribution - $\hat{\rho}_{cMP}$), diamonds (Restricted maximum likelihood estimator for ρ - $\hat{\rho}_{REML}$), triangles (Breslow-Day estimator for ρ based on χ^2 distribution - $\hat{\rho}_{BD}$) and reverse-triangles (Mandel-Paule estimator of ρ - $\hat{\rho}_{MP}$). Light grey line at 0 for bias.

 Copyright © 2016 John Wiley & Sons, Ltd.

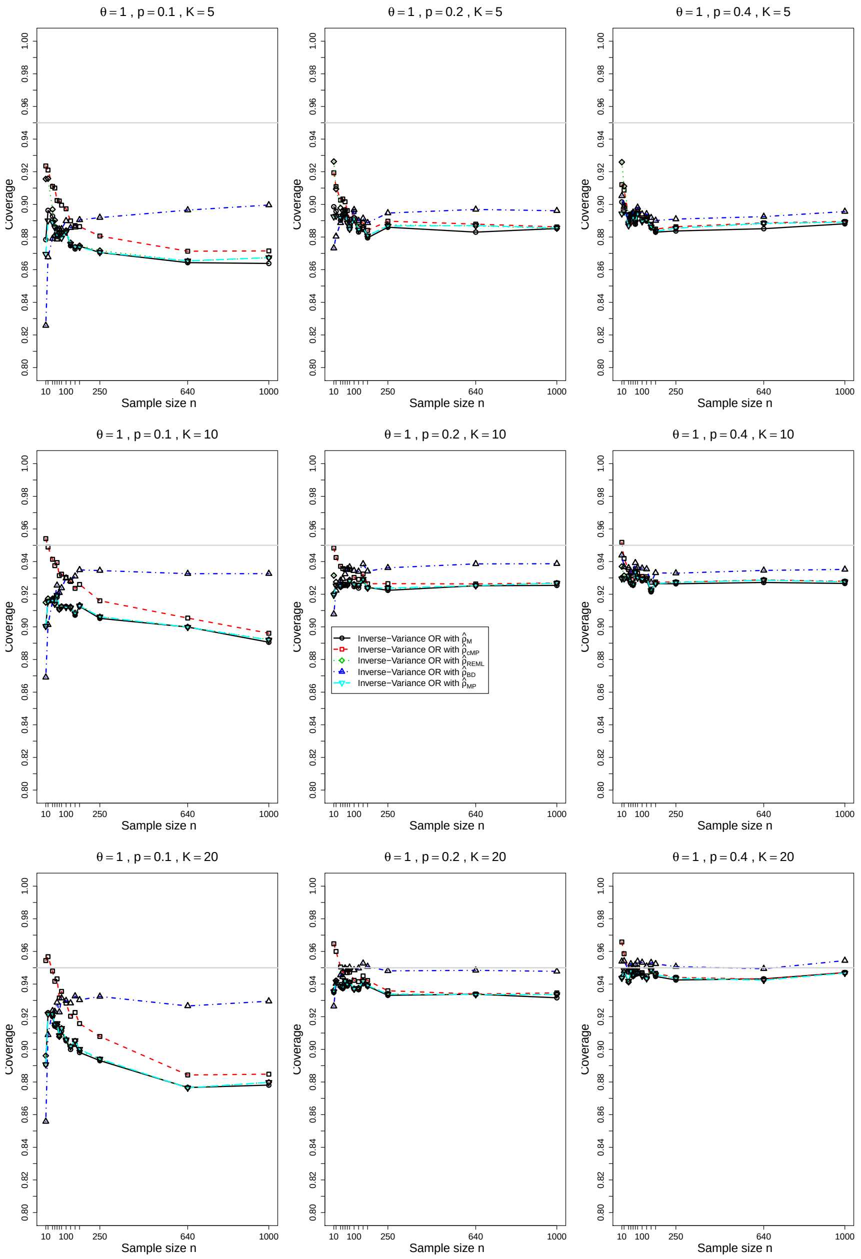

Figure B22. Coverage at the nominal confidence level of 0.95 of the Inverse-Variance overall effect measure θ_{IV} from K studies in beta-binomial model for $0.1 \le p_{2j} \le 0.4$, $\theta = 1$, $\rho = 0.1$ and $10 \le n \le 1000$. The weights of the Mandel-Haenzsel odds ratio use the estimators of ρ: circles (Moment estimator of ρ - $\hat{\rho}_M$), squares (Corrected Mandel-Paule moment estimator for ρ based on gamma approximation for Q distribution - $\hat{\rho}_{cMP}$), diamonds (Restricted maximum likelihood estimator for ρ - $\hat{\rho}_{REML}$), triangles (Breslow-Day estimator for ρ based on χ^2 distribution - $\hat{\rho}_{BD}$) and reverse-triangles (Mandel-Paule estimator of ρ - $\hat{\rho}_{MP}$). Light grey line at 0.95 for coverage.

Copyright © 2016 John Wiley & Sons, Ltd.

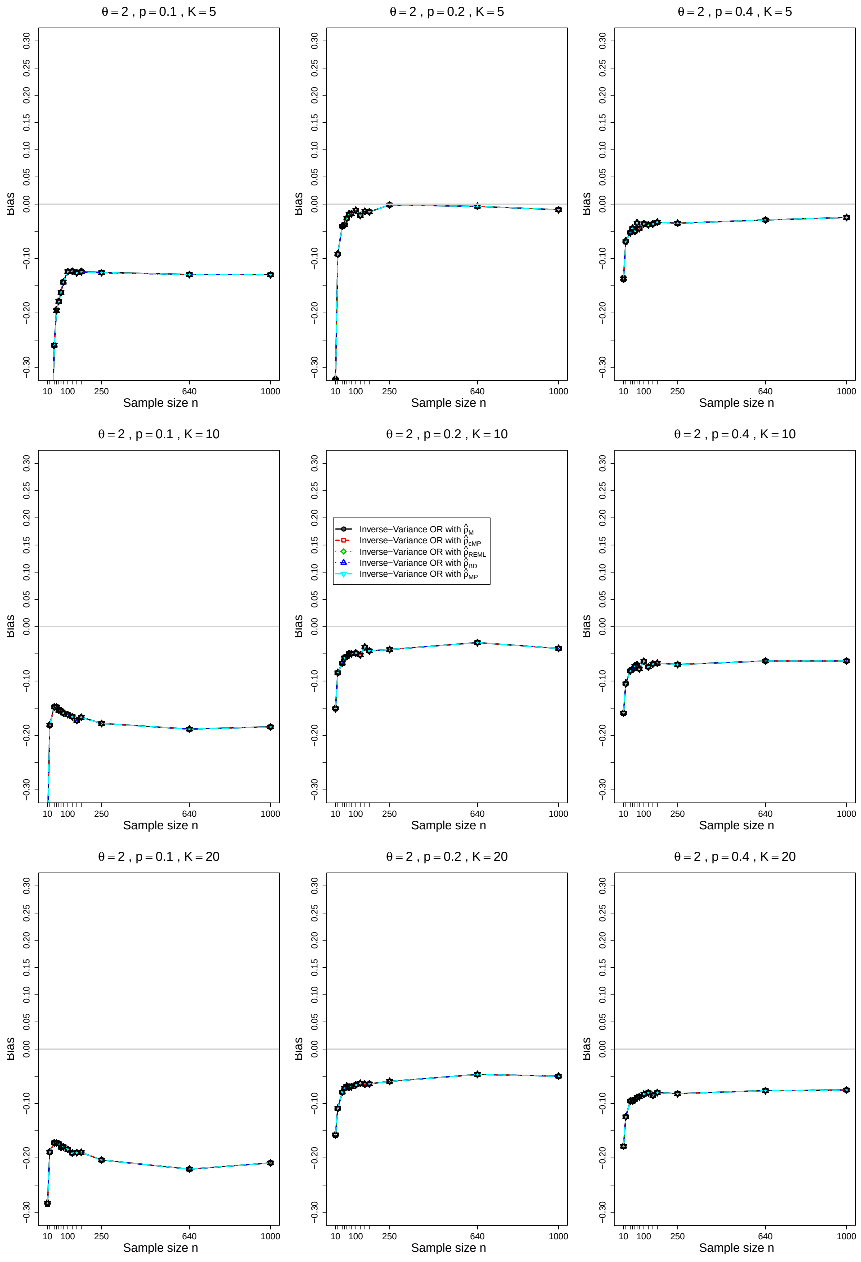

Figure B23. Bias of the inverse-variance overall effect measure ψ_{IV} from K studies in beta-binomial model for $0.1 \leq p_{2j} \leq 0.4$, $\theta = 2$, $\rho = 0.1$ and $10 \leq n \leq 1000$. The inverse-variance weights use the estimators of ρ: circles (Moment estimator of ρ - $\hat{\rho}_M$), squares (Corrected Mandel-Paule moment estimator for ρ based on gamma approximation for Q distribution - $\hat{\rho}_{cMP}$), diamonds (Restricted maximum likelihood estimator for ρ - $\hat{\rho}_{REML}$), triangles (Breslow-Day estimator for ρ based on χ^2 distribution - $\hat{\rho}_{BD}$) and reverse-triangles (Mandel-Paule estimator of ρ - $\hat{\rho}_{MP}$). Light grey line at 0 for bias.

 Copyright © 2016 John Wiley & Sons, Ltd.

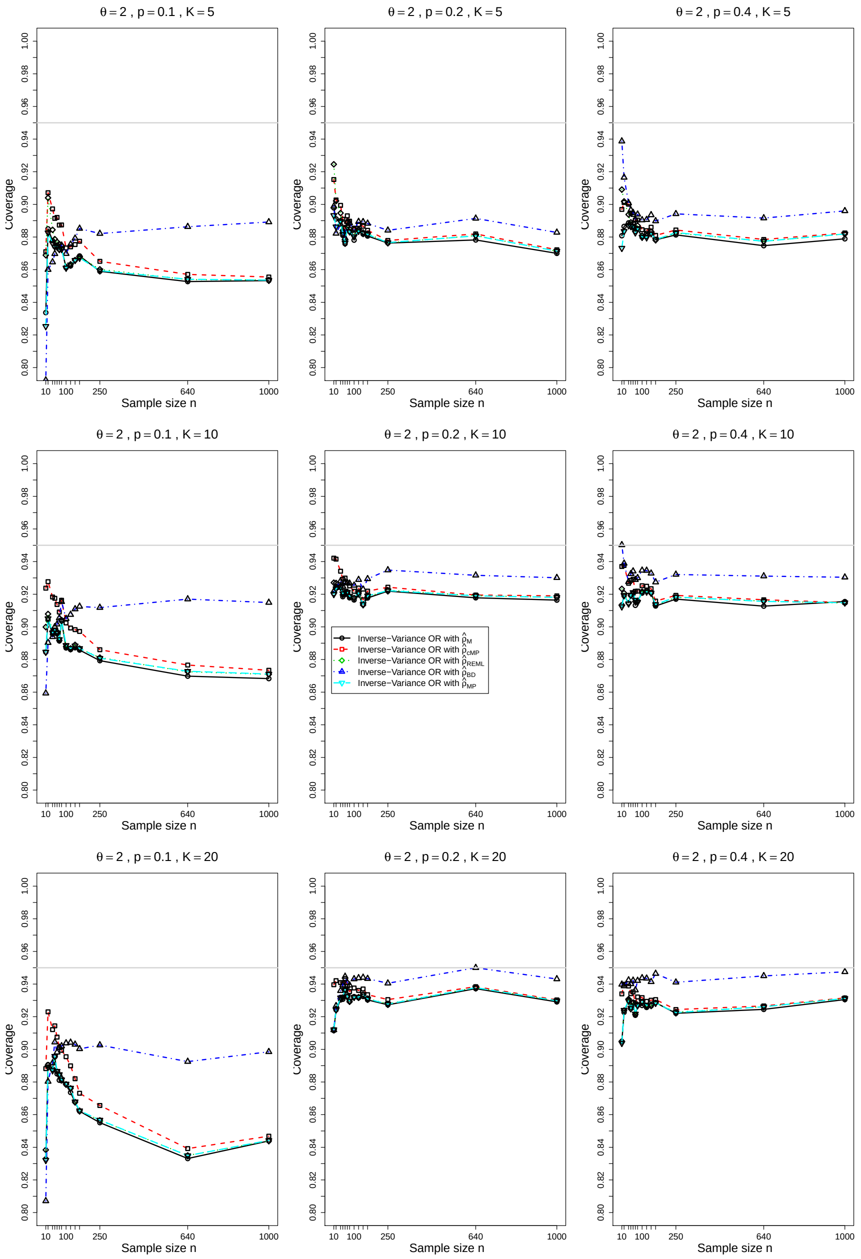

Figure B24. Coverage at the nominal confidence level of 0.95 of the Inverse-Variance overall effect measure θ_{IV} from K studies in beta-binomial model for $0.1 \leq p_{2j} \leq 0.4$, $\theta = 2$, $\rho = 0.1$ and $10 \leq n \leq 1000$. The weights of the Mandel-Haenzsel odds ratio use the estimators of ρ: circles (Moment estimator of ρ - $\hat{\rho}_M$), squares (Corrected Mandel-Paule moment estimator for ρ based on gamma approximation for Q distribution - $\hat{\rho}_{cMP}$), diamonds (Restricted maximum likelihood estimator for ρ - $\hat{\rho}_{REML}$), triangles (Breslow-Day estimator for ρ based on χ^2 distribution - $\hat{\rho}_{BD}$) and reverse-triangles (Mandel-Paule estimator of ρ - $\hat{\rho}_{MP}$). Light grey line at 0.95 for coverage.

Copyright © 2016 John Wiley & Sons, Ltd.

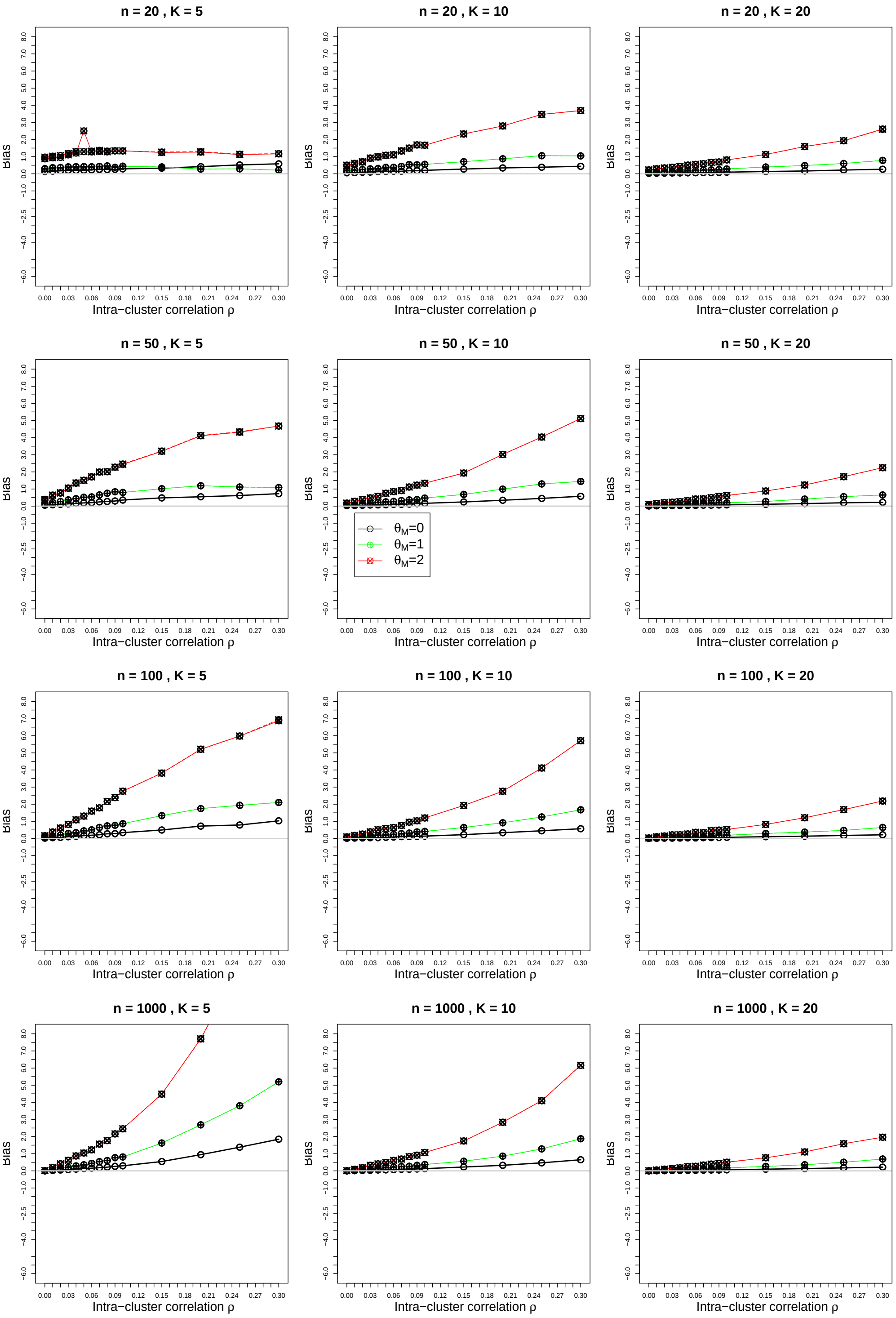

Figure B25. Bias of overall odds ratio $\hat{\psi}_{MH}$ obtained from K studies by the Mantel-Haenzsel method with the moment estimator $\hat{\rho}_M$ in the weights, for $p_{2j} = 0.1$, and $0 \leq \rho \leq 0.3$ for average sample sizes $n = 20,\ 50,\ 100$ and 1000. The biases are given for $\theta = 0$ (circles), $\theta = 1$ (circle plus), and $\theta = 2$ (circle cross). Light grey line at 0 for bias.

B.4. Bias and coverage in estimation of overall effect measure ψ_{MH}

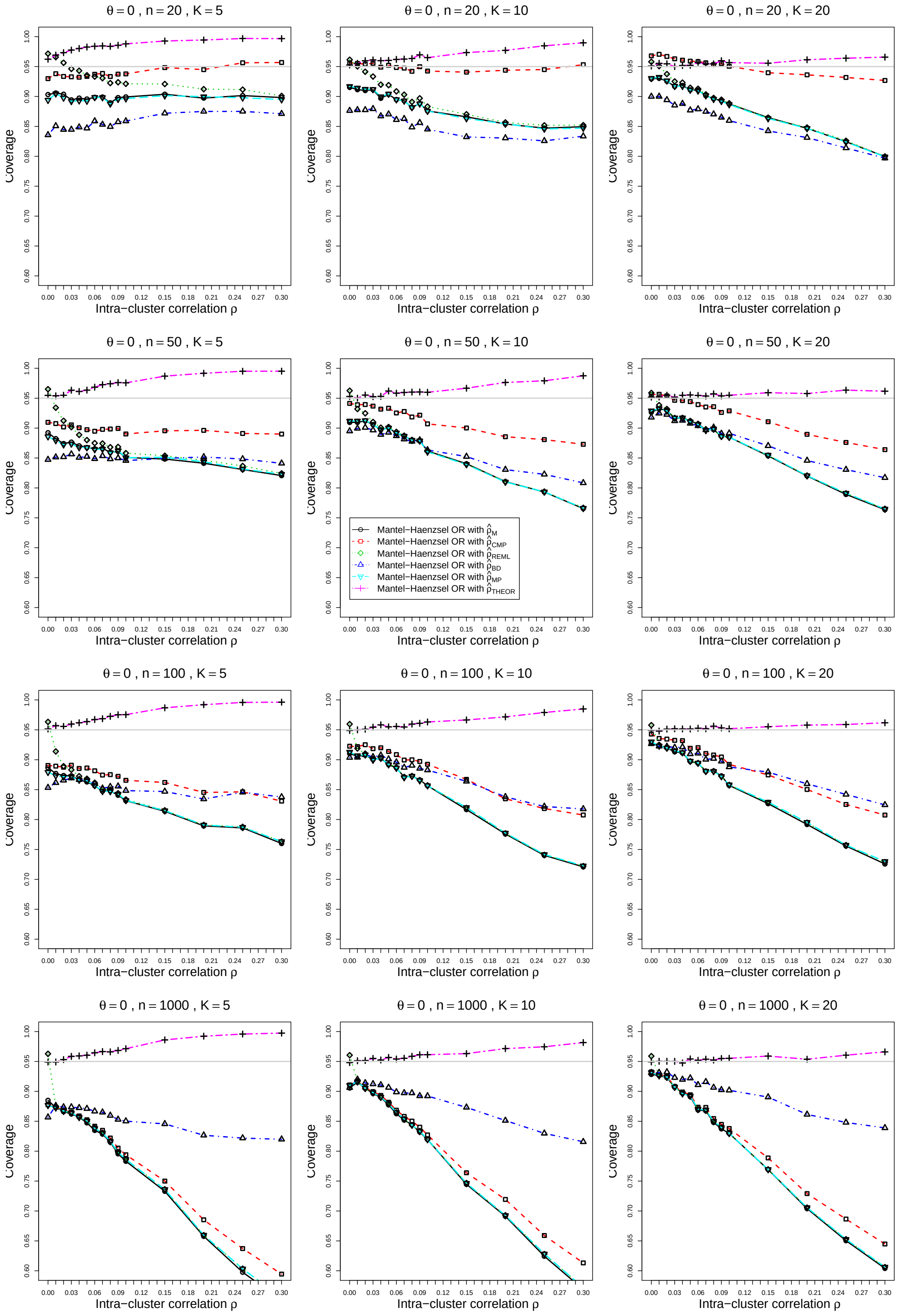

Figure B26. Coverage at the nominal confidence level of 0.95 of the Mantel-Haenzsel overall effect measure ψ_{MH} from K studies in beta-binomial model for $p_{2j} = 0.1, \theta = 0$ and $0 \leq \rho \leq 0.3$. The weights of the Mandel-Haenzsel odds ratio use the estimators of ρ: circles (Moment estimator of ρ - $\hat{\rho}_M$), squares (Corrected Mandel-Paule moment estimator for ρ based on gamma approximation for Q distribution - $\hat{\rho}_{cMP}$), diamonds (Restricted maximum likelihood estimator for ρ - $\hat{\rho}_{REML}$), triangles (Breslow-Day estimator for ρ based on χ^2 distribution - $\hat{\rho}_{BD}$) and reverse-triangles (Mandel-Paule estimator of ρ - $\hat{\rho}_{MP}$). Light grey line at 0.95 for coverage.

Copyright © 2016 John Wiley & Sons, Ltd.

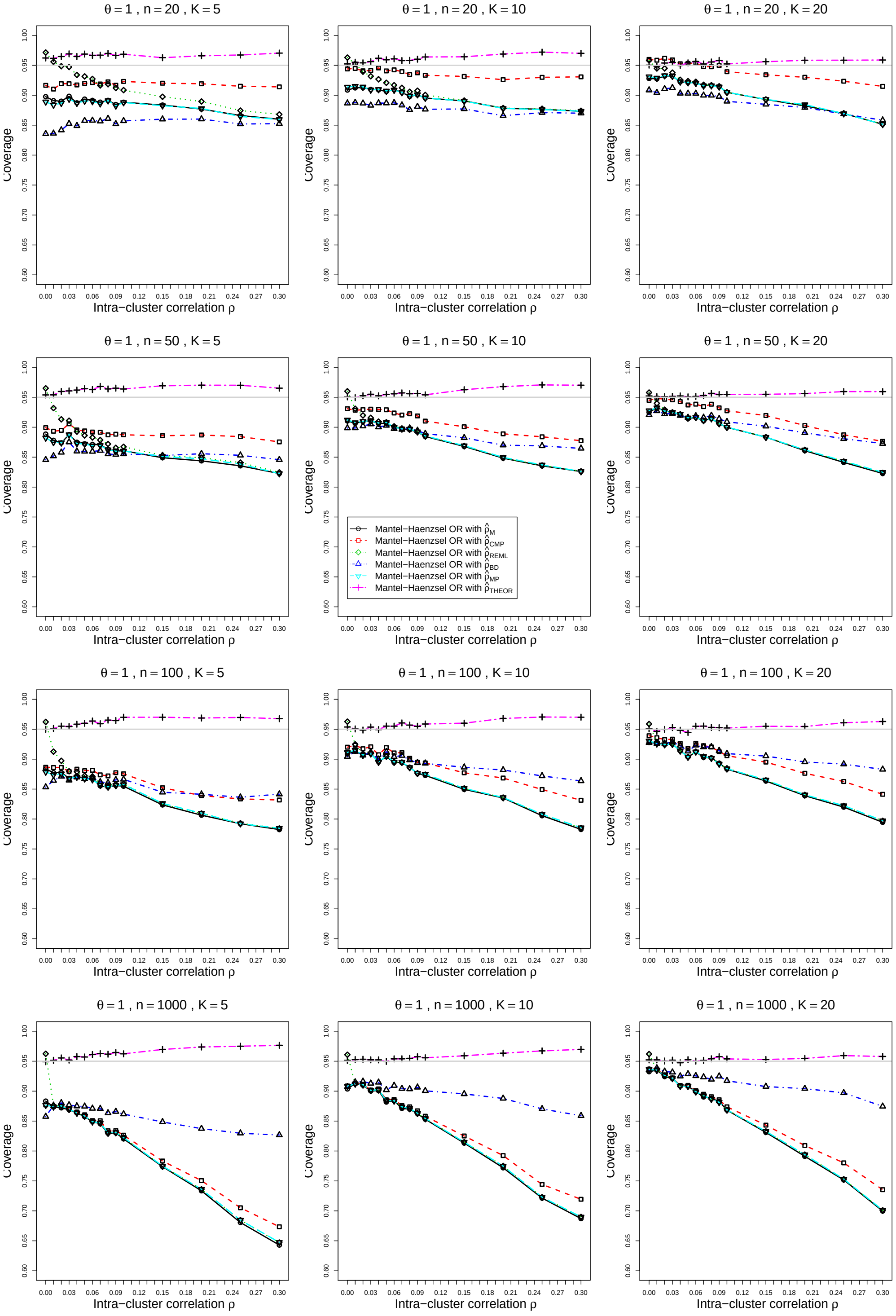

Figure B27. Coverage of the Mantel-Haenszel overall effect measure ψ_{MH} from K studies in beta-binomial model for $p_{2j} = 0.1$, $\theta = 1$ and $0 \leq \rho \leq 0.3$. The weights of the Mantel-Haenszel odds ratio use the estimators of ρ: circles (Moment estimator of ρ - $\hat{\rho}_M$), squares (Corrected Mandel-Paule moment estimator for ρ based on gamma approximation for Q distribution - $\hat{\rho}_{cMP}$), diamonds (Restricted maximum likelihood estimator for ρ - $\hat{\rho}_{REML}$), triangles (Breslow-Day estimator for ρ based on χ^2 distribution - $\hat{\rho}_{BD}$) and reverse-triangles (Mandel-Paule estimator of ρ - $\hat{\rho}_{MP}$). Light grey line at 0 for bias.

 Copyright © 2016 John Wiley & Sons, Ltd.

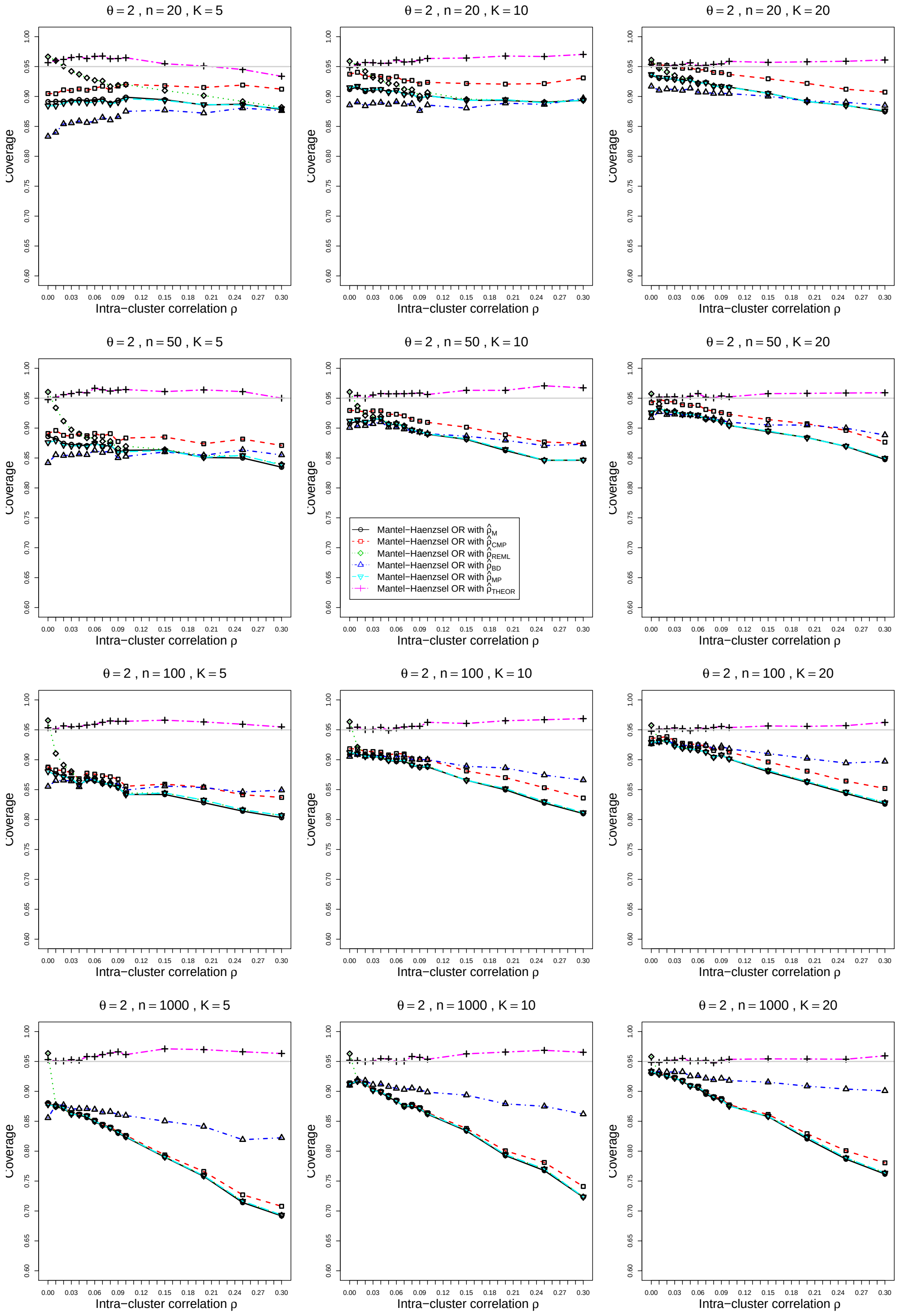

Figure B28. Coverage of the Mantel-Haenszel overall effect measure ψ_{MH} from K studies in beta-binomial model for $p_{2j} = 0.1$, $\theta = 2$ and $0 \leq \rho \leq 0.3$. The weights of the Mantel-Haenszel odds ratio use the estimators of ρ: circles (Moment estimator of ρ - $\hat{\rho}_M$), squares (Corrected Mandel-Paule moment estimator for ρ based on gamma approximation for Q distribution - $\hat{\rho}_{cMP}$), diamonds (Restricted maximum likelihood estimator for ρ - $\hat{\rho}_{REML}$), triangles (Breslow-Day estimator for ρ based on χ^2 distribution - $\hat{\rho}_{BD}$) and reverse-triangles (Mandel-Paule estimator of ρ - $\hat{\rho}_{MP}$). Light grey line at 0 for bias.

Copyright © 2016 John Wiley & Sons, Ltd.

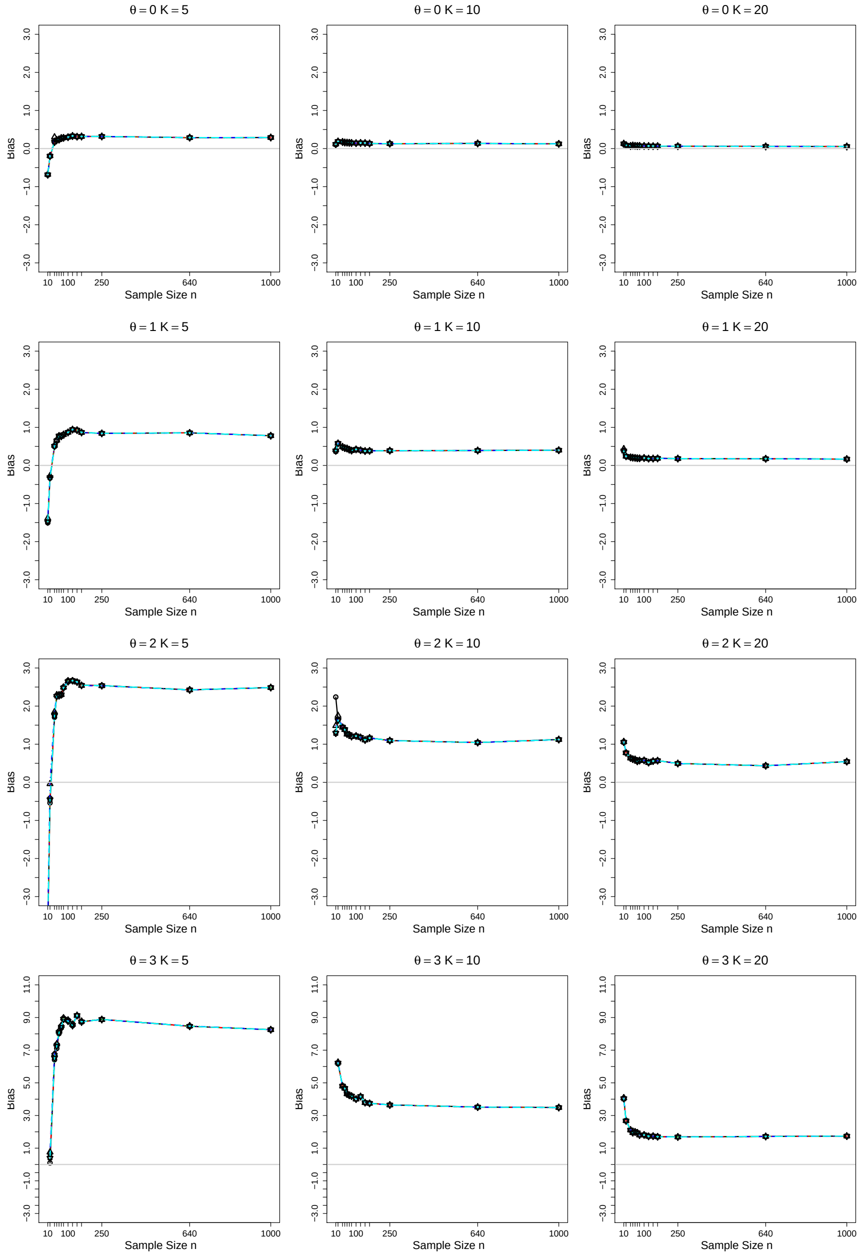

Figure B29. Bias of the Mantel-Haenzsel overall effect measure ψ_{MH} from K studies in beta-binomial model for $p_{2j} = 0.1, 0 \leq \theta \leq 3, \rho = 0.1$ and $10 \leq n \leq 1000$. The weights of the Mantel-Haenszel odds ratio use the estimators of ρ: circles (Moment estimator of ρ - $\hat{\rho}_M$), squares (Corrected Mandel-Paule moment estimator for ρ based on gamma approximation for Q distribution - $\hat{\rho}_{cMP}$), diamonds (Restricted maximum likelihood estimator for ρ - $\hat{\rho}_{REML}$), triangles (Breslow-Day estimator for ρ based on χ^2 distribution - $\hat{\rho}_{BD}$) and reverse-triangles (Mandel-Paule estimator of ρ - $\hat{\rho}_{MP}$). Light grey line at 0 for bias.

 Copyright © 2016 John Wiley & Sons, Ltd.

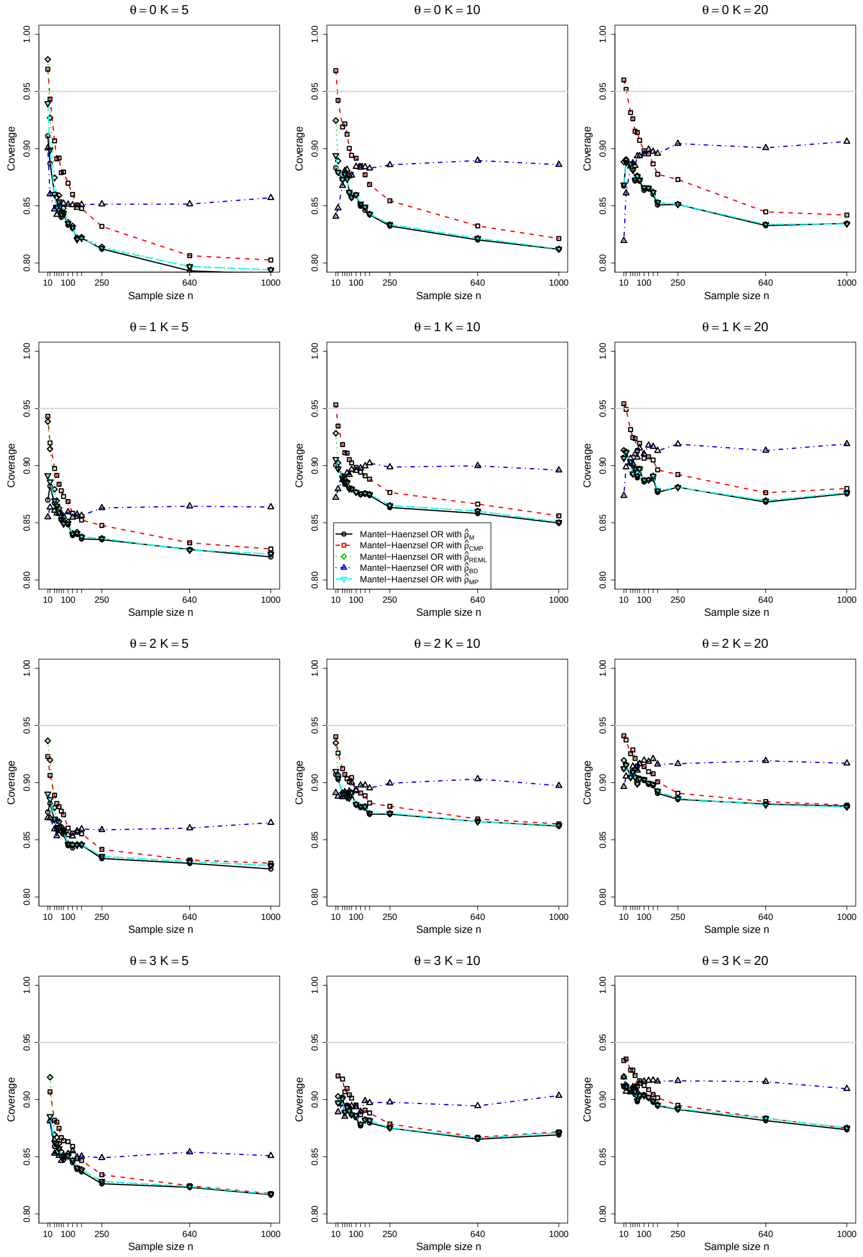

Figure B30. Coverage at the nominal confidence level of 0.95 of the Mantel-Haenzsel overall effect measure ψ_{MH} from K studies in beta-binomial model for $p_{2j} = 0.1, 0 \leq \theta \leq 3$, $\rho = 0.1$ and $10 \leq n \leq 1000$. The weights of the Mandel-Haenzsel odds ratio use the estimators of ρ: circles (Moment estimator of ρ - $\hat{\rho}_M$), squares (Corrected Mandel-Paule moment estimator for ρ based on gamma approximation for Q distribution - $\hat{\rho}_{cMP}$), diamonds (Restricted maximum likelihood estimator for ρ - $\hat{\rho}_{REML}$), triangles (Breslow-Day estimator for ρ based on χ^2 distribution - $\hat{\rho}_{BD}$) and reverse-triangles (Mandel-Paule estimator of ρ - $\hat{\rho}_{MP}$). Light grey line at 0.95 for coverage.

Copyright © 2016 John Wiley & Sons, Ltd.

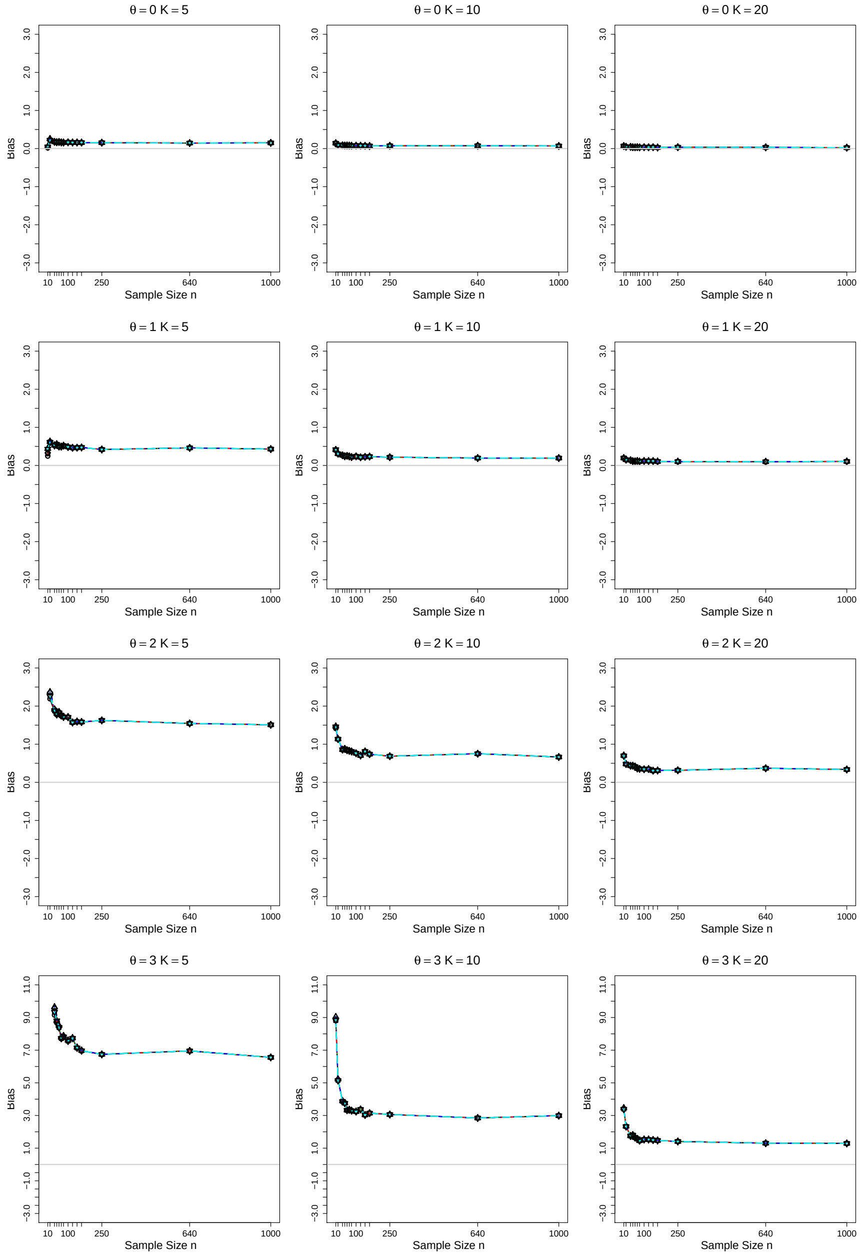

Figure B31. Bias of the Mantel-Haenzsel overall effect measure ψ_{MH} from K studies in beta-binomial model for $p_{2j} = 0.2, 0 \leq \theta \leq 3, \rho = 0.1$ and $10 \leq n \leq 1000$. The weights of the Mantel-Haenzsel odds ratio use the estimators of ρ: circles (Moment estimator of ρ - $\hat{\rho}_M$), squares (Corrected Mandel-Paule moment estimator for ρ based on gamma approximation for Q distribution - $\hat{\rho}_{cMP}$), diamonds (Restricted maximum likelihood estimator for ρ - $\hat{\rho}_{REML}$), triangles (Breslow-Day estimator for ρ based on χ^2 distribution - $\hat{\rho}_{BD}$) and reverse-triangles (Mandel-Paule estimator of ρ - $\hat{\rho}_{MP}$). Light grey line at 0 for bias.

 Copyright © 2016 John Wiley & Sons, Ltd.

Statistics
in Medicine

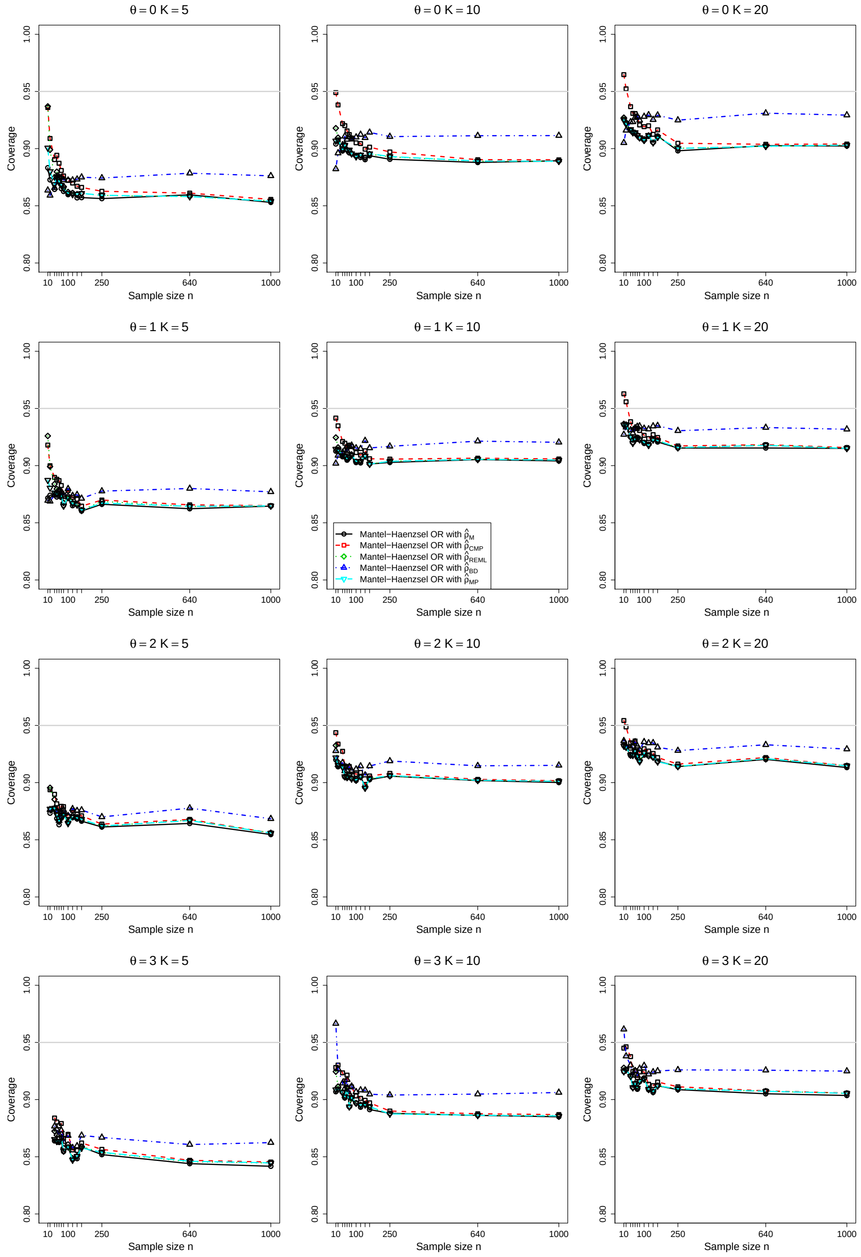

Figure B32. Coverage at the nominal confidence level of 0.95 of the Mantel-Haenszel overall effect measure ψ_{MH} from K studies in beta-binomial model for $p_{2j} = 0.2$, $0 \leq \theta \leq 3$, $\rho = 0.1$ and $10 \leq n \leq 1000$. The weights of the Mandel-Haenszel odds ratio use the estimators of ρ: circles (Moment estimator of ρ - $\hat{\rho}_M$), squares (Corrected Mandel-Paule moment estimator for ρ based on gamma approximation for Q distribution - $\hat{\rho}_{cMP}$), diamonds (Restricted maximum likelihood estimator for ρ - $\hat{\rho}_{REML}$), triangles (Breslow-Day estimator for ρ based on χ^2 distribution - $\hat{\rho}_{BD}$) and reverse-triangles (Mandel-Paule estimator of ρ - $\hat{\rho}_{MP}$). Light grey line at 0.95 for coverage.

Copyright © 2016 John Wiley & Sons, Ltd.

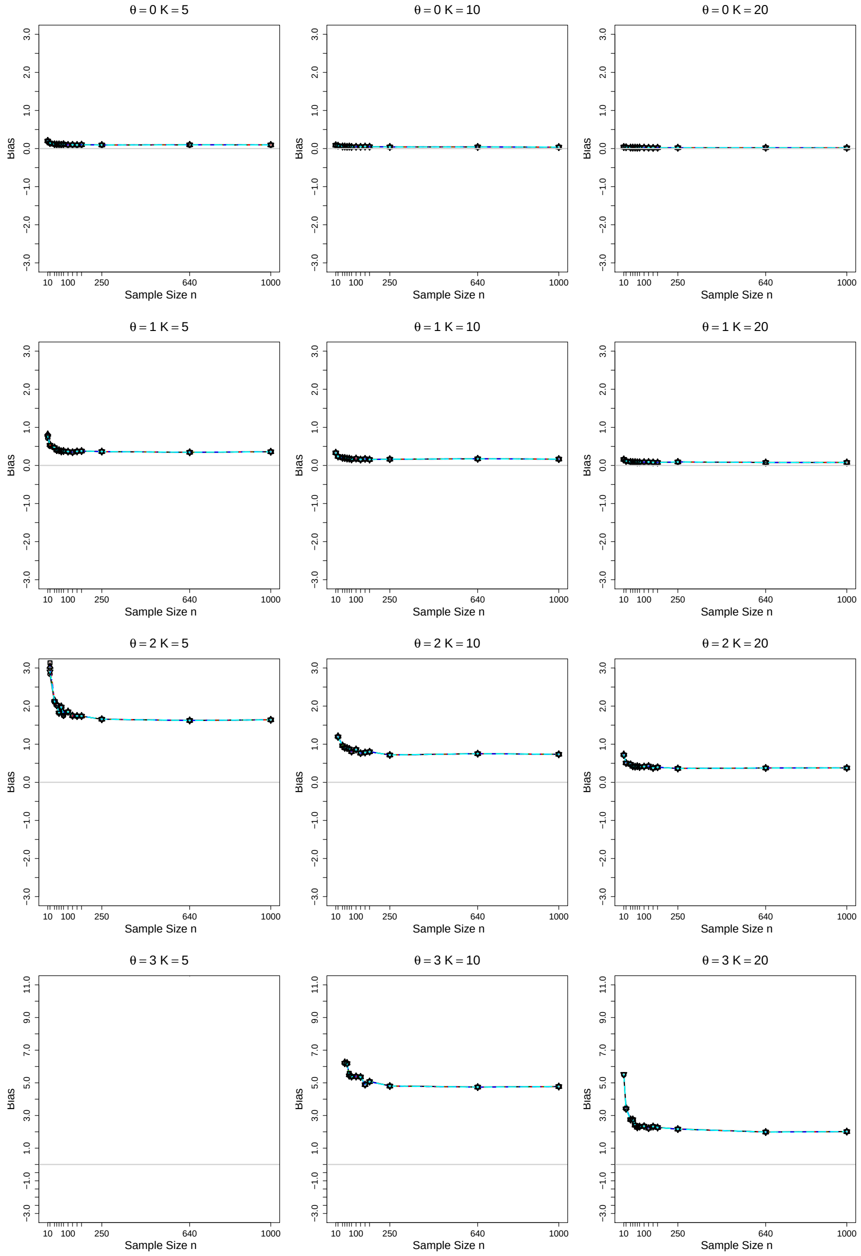

Figure B33. Bias of the Mantel-Haenzsel overall effect measure ψ_{MH} from K studies in beta-binomial model for $p_{2j} = 0.4, 0 \leq \theta \leq 3, \rho = 0.1$ and $10 \leq n \leq 1000$. The weights of the Mantel-Haenzsel odds ratio use the estimators of ρ: circles (Moment estimator of ρ - $\hat{\rho}_M$), squares (Corrected Mandel-Paule moment estimator for ρ based on gamma approximation for Q distribution - $\hat{\rho}_{cMP}$), diamonds (Restricted maximum likelihood estimator for ρ - $\hat{\rho}_{REML}$), triangles (Breslow-Day estimator for ρ based on χ^2 distribution - $\hat{\rho}_{BD}$) and reverse-triangles (Mandel-Paule estimator of ρ - $\hat{\rho}_{MP}$). Light grey line at 0 for bias.

 Copyright © 2016 John Wiley & Sons, Ltd.

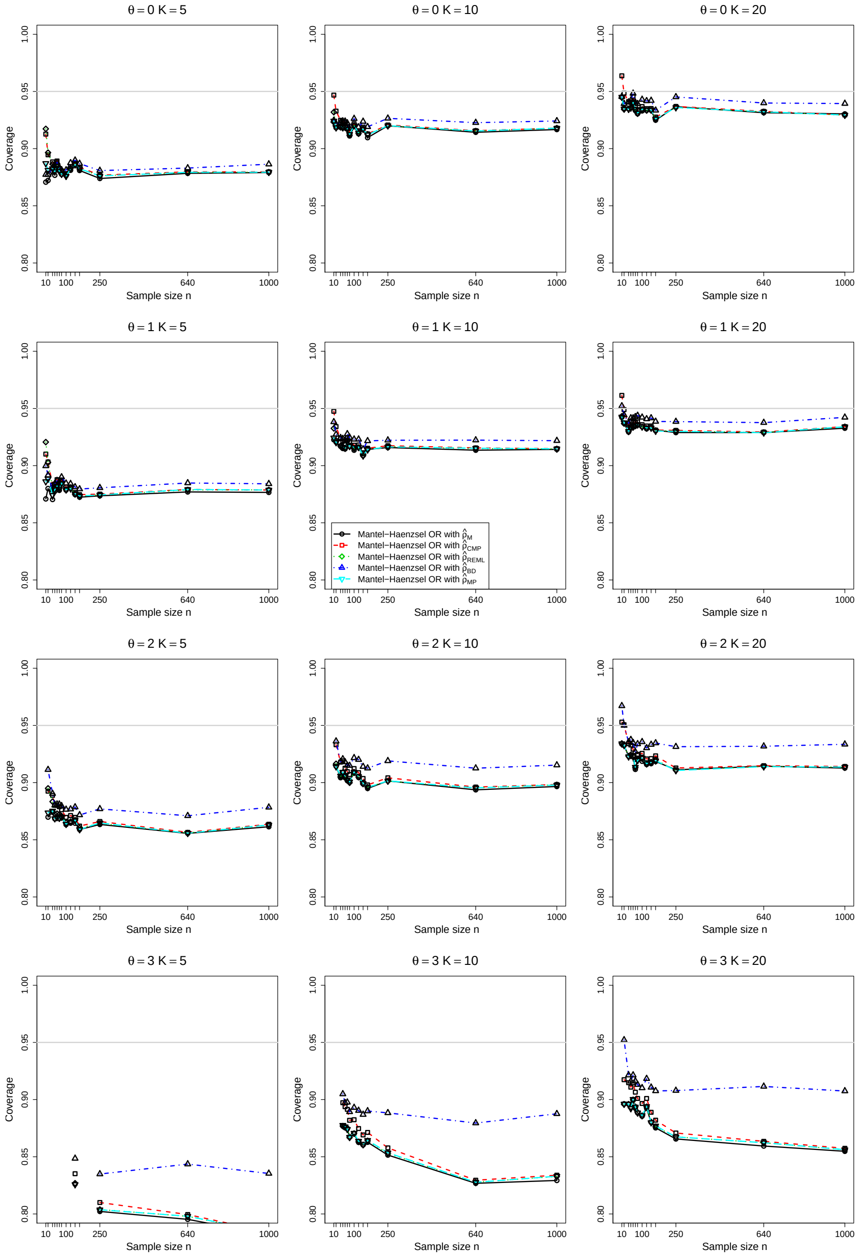

Figure B34. Coverage at the nominal confidence level of 0.95 of the Mantel-Haenszel overall effect measure ψ_{MH} from K studies in beta-binomial model for $p_{2j} = 0.4$, $0 \leq \theta \leq 3$, $\rho = 0.1$ and $10 \leq n \leq 1000$. The weights of the Mandel-Haenszel odds ratio use the estimators of ρ: circles (Moment estimator of ρ - $\hat{\rho}_M$), squares (Corrected Mandel-Paule moment estimator for ρ based on gamma approximation for Q distribution - $\hat{\rho}_{cMP}$), diamonds (Restricted maximum likelihood estimator for ρ - $\hat{\rho}_{REML}$), triangles (Breslow-Day estimator for ρ based on χ^2 distribution - $\hat{\rho}_{BD}$) and reverse-triangles (Mandel-Paule estimator of ρ - $\hat{\rho}_{MP}$). Light grey line at 0.95 for coverage.

Copyright © 2016 John Wiley & Sons, Ltd.

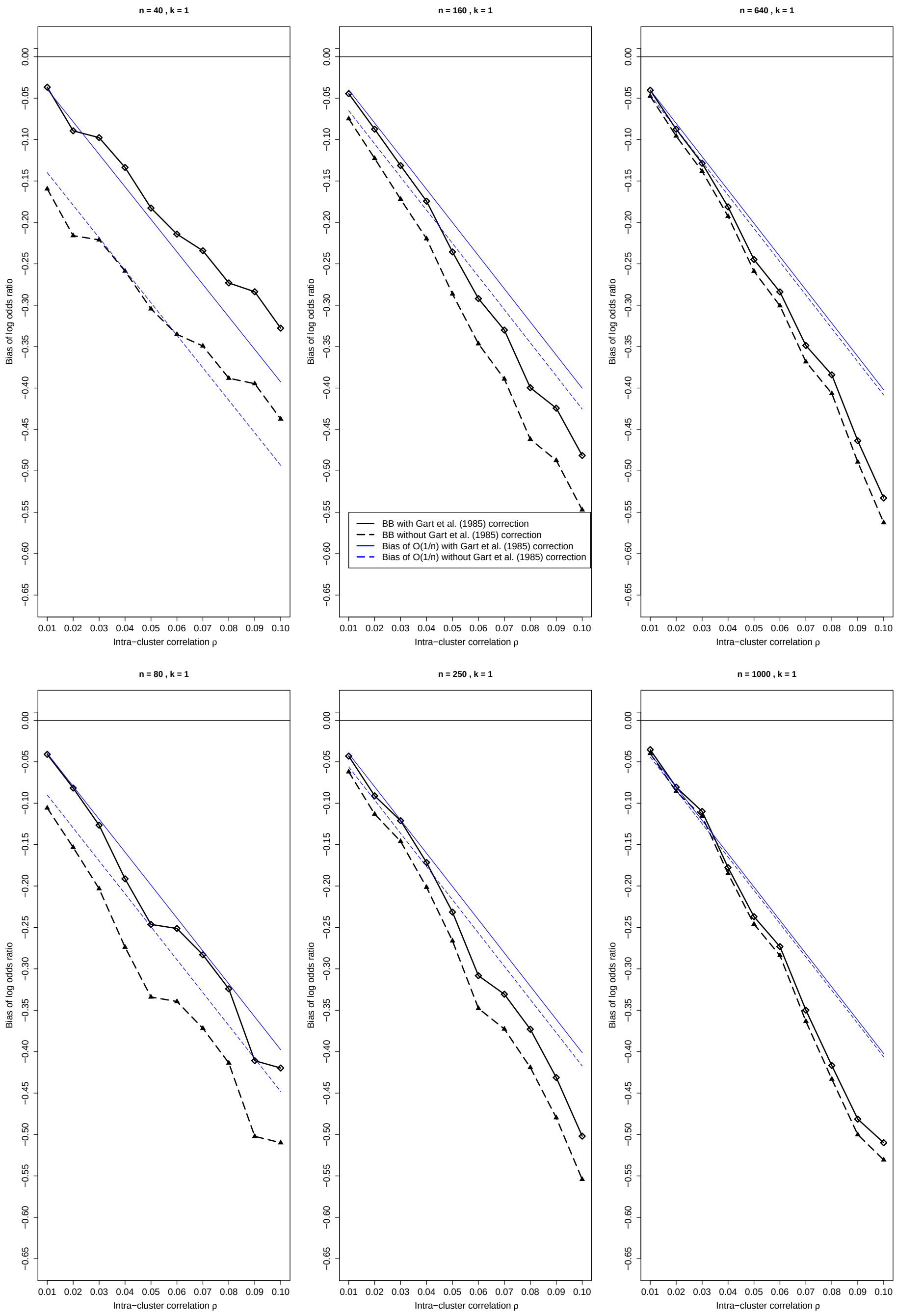

Figure C35. Bias of log-odds ratio in overdispersed binomial model for $p_{1j} = 0.1$ ($\log(p_{1j}/(1-p_{1j})) = -2.20$) and $p_{2j} = 0.4$ ($\log(p_{2j}/(1-p_{2j})) = -0.40$) and $0 \leq \rho \leq 0.1$. 10000 simulations for each value of ρ from the beta-binomial distribution (black); the first order bias term given by the first two terms of equation (6.2.3) on page 10 with known values of p and ρ (blue), with and without the continuity correction (solid and dashed lines, respectively)

C. Transformation Bias of $\hat{\theta}$

 Copyright © 2016 John Wiley & Sons, Ltd.

D. Standard REM inference when data are generated from Beta-Binomial Model

In this section we provide a simulation study to access the performance of point and interval estimators of random effect parameter τ^2 and the combined LOR θ in REM-based meta-analysis of overdispersed binary data. In contrast to the simulation study in section 6 of the main text, we estimate between-study variance as in REM rather than the intra-cluster correlation as in ODM. Our main goal is to check the robustness of standard methods in meta-analysis of LOR.
We assess six point estimators of τ^2 in respect to their bias: the DerSimonian and Laird method, the Mandel-Paule inspired method τ^2_{MP}, the corrected Mandel-Paule estimator based on the gamma approximation to Q distribution τ^2_{CMP} similar to that in section 4.3, the ML method, the REML method, and the Breslow-Day-based method similar to that described in section 4.4. We also assess four related confidence intervals for τ^2: Q-profile confidence interval, corrected Q-profile confidence interval as in section 4.3, profiled-likelihood confidence interval and profiled Breslow-Day confidence interval as in section 4.4 in respect to their coverage at the 95% confidence level. We also assess bias and coverage of the combined odds ratio or its log obtained by inverse-variance method $\hat{\theta}_w = \sum w_i(\tau^2)\hat{\theta}_i / \sum w_i(\tau^2)$.

D.1. Correspondence between ρ and τ^2

The second moment of $\hat{\theta}$ is the same in REM and ODM, when the sample sizes and the probabilities are constant across studies, equation (3.3). The standard REM-based methods would aim to estimate the between study variance τ^2. Table 1 shows the values of τ^2 obtained from equation (3.3) for sample sizes $n = (20, 50, 100, 1000)$. In the Table 1, overdispersion parameter ρ varies between 0 and 0.1 (small to moderate heterogeneity) in steps of 0.01, and between 0.1 and 0.3 in steps of 0.05 (moderate to large heterogeneity). Log odds ratio $\theta = 0$, 1 and 2. For $p_{2i} = 0.1$, the values of $\theta = 0$, 1 and 2 correspond to $p_{1i} = 0.1$, 0.23 and 0.45, respectively.

D.2. Simulation design

Sizes of the control and treatment groups were taken equal $n_{1i} = n_{2i} = n_i$ across K studies. For a given probability p_{2i}, the number of cases in the control group X_{2i} was simulated from a beta-binomial (n_{2i}, p_{2i}, ρ) distribution. The number of cases in the treatment group X_{1i} was generated from a beta-binomial (n_{1i}, p_{1i}, ρ) distribution with $p_{1i} = p_{2i}\exp(\theta)/(1 - p_{2i} + p_{2i}\exp(\theta))$ for a given LOR value of θ. When $\rho = 0$, the numbers of events for treatment and control arm X_{ji} were generated from binomial distributions with sample size n_{ji} and probabilities p_{ji}, preserving the above relationship between the probabilities in the treatment and control arms.
The following configurations of parameters were included in the simulations. The number of studies $K = (5, 10, 20)$; sample sizes in each arm are constant $n = (20, 50, 100, 1000)$; overdispersion parameter ρ varies between 0 and 0.1 (small to moderate heterogeneity) in steps 0.01, and between 0.1 and 0.3 in steps 0.05 (moderate to large heterogeneity). The corresponding value of between study variance τ^2 is obtained through equation (3.3). The values of LOR θ vary from 0 to 2 in steps of 1. The probability in the control group p_{2i} takes values 0.1, 0.2, 0.4. A total of 10000 simulations were produced for each combination.

D.3. Simulation results

Figures D36 to D41 show the bias and coverage of τ^2 estimated by the above listed methods for different combinations of K and n for the case of $p_{2i} \equiv 0.1$ and $\theta = 0,\ 1,\ 2$ for varying values of $0 \leq \rho \leq 0.3$. The corresponding true values of τ^2 were obtained from equation (3.3). The bias and coverage of true log odds ratio θ estimated by the inverse-variance method (θ_{IV}) using τ^2_{cMP} for values of $\theta = 0$, 1, 2, are shown in Figures D42 - D45, respectively. Other methods of τ^2 estimation provide very similar results for estimation of θ.

In respect to bias, τ^2_{cMP} performs the best for $n = 20$, but its performance, as well as the performance of τ^2_{BD}, deteriorates for larger sample sizes. In contrast, the standard REM methods perform reasonably well for $n = 50$ to 100, especially so for not too large values of τ^2. Finally, for large sample sizes ($n = 1000$) only the DerSimonian-Laird method provides a robust estimate of τ^2 for all values of θ. The confidence intervals for τ^2 behave similarly, with the Q-gamma-profile method being the best for $n = 20$, Q-profile and profile-likelihood for $n = 50$ and 100, and all methods deteriorating for $n = 1000$. It is interesting to compare the biases in estimation of ρ and τ^2 which appear to be in opposite direction, i.e. all methods tend to underestimate ρ (Figure 1), but to overestimate τ^2. We believe that this is due to additional estimation of probabilities included in equation (3.3). It is not clear how much substance the above results carry, as we are estimating a parameter τ^2 which is not really relevant to the true model.

Copyright © 2016 John Wiley & Sons, Ltd.

The bias of LOR θ is very considerable for $\theta = 1$ and 2, but it is once more in the opposite direction to that of Figure 3 in the main text. This bias does not visibly depend on the method of estimation of τ^2. The coverage of θ based on the Breslow-Day method is very good, but all the standard methods fail very impressively.

D.4. Methods to differentiate between REM and ODM

A good summary of existing diagnostic methods to differentiate between beta-binomial and logistic-normal model is provided by [1]. Among these, [2] suggests to fit the beta-binomial model and plot standardized residuals against fitted values. If plot indicates that the variance of these residuals decreases markedly as the fitted value approaches zero or one, then standard random effects model may be more appropriate. Another graphical method, the plot of half-normal scores against deviance residuals, is proposed by [1]. In regression analysis for binary data, the plots of standardized or deviance residuals against fitted values are particularly useful in checking the model adequacy, detecting outliers or unusual observations. However, the use of these plots to choose between models may not be the best option. According to [3], it is generally difficult to differentiate between beta-binomial and logistic-normal models. Referencing the paper by [2], [4] suggests that it is only possible to see the difference between models if there are large numbers of observations with fitted values close to zero or one. [5] warn that ”when the probabilities are small and the data are highly discrete, only limited information is present for estimating the random effects”. [6] discuss the case of sparse dependent binary data. They point out that for the asymptotic situation, when the number of studies is increasing, the maximum likelihood theory leads to inconsistent maximum likelihood estimation for odds ratio regression models. Therefore, [6] do not recommend the use of likelihood based methods for the dependent binary data, since these methods are not robust against model specification. This agrees with our results in Section D, where the moment-based DerSimonian-Laird method was considerably more robust to model misspecification than the likelihood-based methods.

References

1. Hinde J, Demétrio CG. Overdispersion: models and estimation. *Computational Statistics & Data Analysis* 1998; **27**(2):151–170.
2. Williams DA. Extra-binomial variation in logistic linear models. *Applied statistics* 1982; :144–148.
3. Gelman A, Hill J. *Data analysis using regression and multilevel/hierarchical models*. Cambridge University Press, 2006.
4. Ganio-Gibbons LM. *Diagnostic tools for overdispersion in generalized linear models*. PhD Thesis, Oregon State University, 1989. URL `http://hdl.handle.net/1957/38199`.
5. Breslow NE, Clayton DG. Approximate inference in generalized linear mixed models. *Journal of the American statistical Association* 1993; **88**(421):9–25.
6. Hanfelt JJ, Liang KY. Inference for odds ratio regression models with sparse dependent data. *Biometrics* 1998; :136–147.

 Copyright © 2016 John Wiley & Sons, Ltd.

Table 1. Values of τ^2 obtained from the correspondence between ρ and τ^2, equation (3.3)

			ρ													
N	p_C	p_T	0.01	0.02	0.03	0.04	0.05	0.06	0.07	0.08	0.09	0.1	0.15	0.2	0.25	0.3
20	0.1	0.1	0.21	0.42	0.63	0.84	1.05	1.26	1.47	1.68	1.9	2.11	3.16	4.22	5.27	6.33
50			0.21	0.43	0.65	0.87	1.08	1.30	1.52	1.74	1.96	2.17	3.26	4.35	5.44	6.53
100			0.22	0.44	0.66	0.88	1.1	1.32	1.54	1.76	1.98	2.2	3.3	4.4	5.5	6.6
1000			0.22	0.44	0.66	0.88	1.11	1.33	1.55	1.77	1.99	2.22	3.33	4.44	5.55	6.66
20	0.2	0.2	0.11	0.23	0.35	0.47	0.59	0.71	0.83	0.95	1.06	1.18	1.78	2.37	2.96	3.56
50			0.12	0.24	0.36	0.49	0.61	0.73	0.85	0.98	1.10	1.22	1.83	2.45	3.06	3.67
100			0.12	0.24	0.37	0.49	0.61	0.74	0.86	0.99	1.11	1.23	1.85	2.47	3.09	3.71
1000			0.12	0.24	0.37	0.49	0.62	0.74	0.87	0.99	1.12	1.24	1.87	2.49	3.12	3.74
20	0.4	0.4	0.07	0.15	0.23	0.31	0.39	0.47	0.55	0.63	0.71	0.79	1.18	1.58	1.97	2.37
50			0.08	0.16	0.24	0.32	0.40	0.49	0.57	0.65	0.73	0.81	1.22	1.63	2.04	2.45
100			0.08	0.16	0.24	0.33	0.41	0.49	0.57	0.66	0.74	0.82	1.23	1.65	2.06	2.47
1000			0.08	0.16	0.24	0.33	0.41	0.49	0.58	0.66	0.74	0.83	1.24	1.66	2.08	2.49
20	0.1	0.23	0.15	0.31	0.47	0.63	0.79	0.95	1.11	1.27	1.43	1.59	2.38	3.18	3.97	4.77
50			0.16	0.32	0.49	0.65	0.82	0.98	1.14	1.31	1.47	1.64	2.46	3.28	4.10	4.92
100			0.16	0.33	0.49	0.66	0.82	0.99	1.16	1.32	1.49	1.65	2.48	3.31	4.14	4.97
1000			0.16	0.33	0.50	0.66	0.83	1.01	1.17	1.33	1.50	1.67	2.51	3.34	4.18	5.02
20	0.1	0.4	0.14	0.29	0.43	0.58	0.72	0.87	1.01	1.16	1.30	1.45	2.17	2.90	3.62	4.35
50			0.14	0.29	0.44	0.59	0.74	0.89	1.04	1.19	1.34	1.49	2.24	2.99	3.74	4.49
100			0.15	0.30	0.45	0.60	0.75	0.90	1.05	1.21	1.36	1.51	2.26	3.02	3.78	4.53
1000			0.15	0.30	0.45	0.61	0.76	0.91	1.06	1.22	1.37	1.52	2.28	3.05	3.81	4.57

Copyright © 2016 John Wiley & Sons, Ltd.

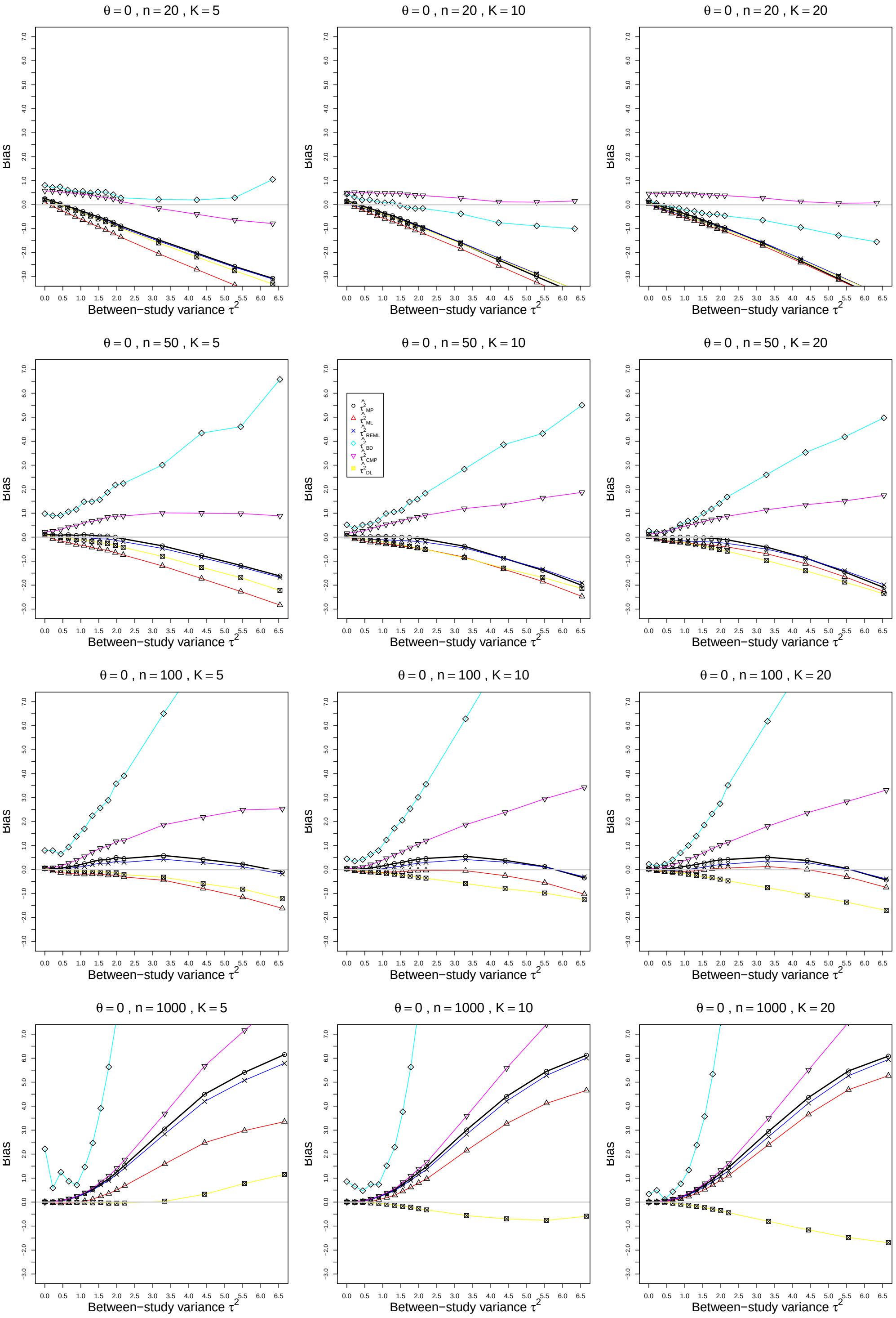

Figure D36. Bias estimated from K studies of the between study variance τ^2 in the beta-binomial model for $p_{2j} = 0.1, \theta = 0$ and $0 \leq \tau^2 \leq 6.5$ (equivalently $0 \leq \rho \leq 0.3$) for constant sample sizes $n = 20,\ 50,\ 100$ and 1000. Estimation methods: circles – Mandel-Paule estimator $\hat{\tau}^2_{MP}$, triangles – $\hat{\tau}^2_{ML}$, filled squares – Moment estimator $\hat{\tau}^2_{DL}$, crosses – $\hat{\tau}^2_{REML}$, diamonds- Breslow-Day based estimator $\hat{\tau}^2_{BD}$, reverse-triangles – Corrected Mandel-Paule estimator $\hat{\tau}^2_{cMP}$. Light grey line at 0.

D.5. Bias and coverage of between study variance

 Copyright © 2016 John Wiley & Sons, Ltd.

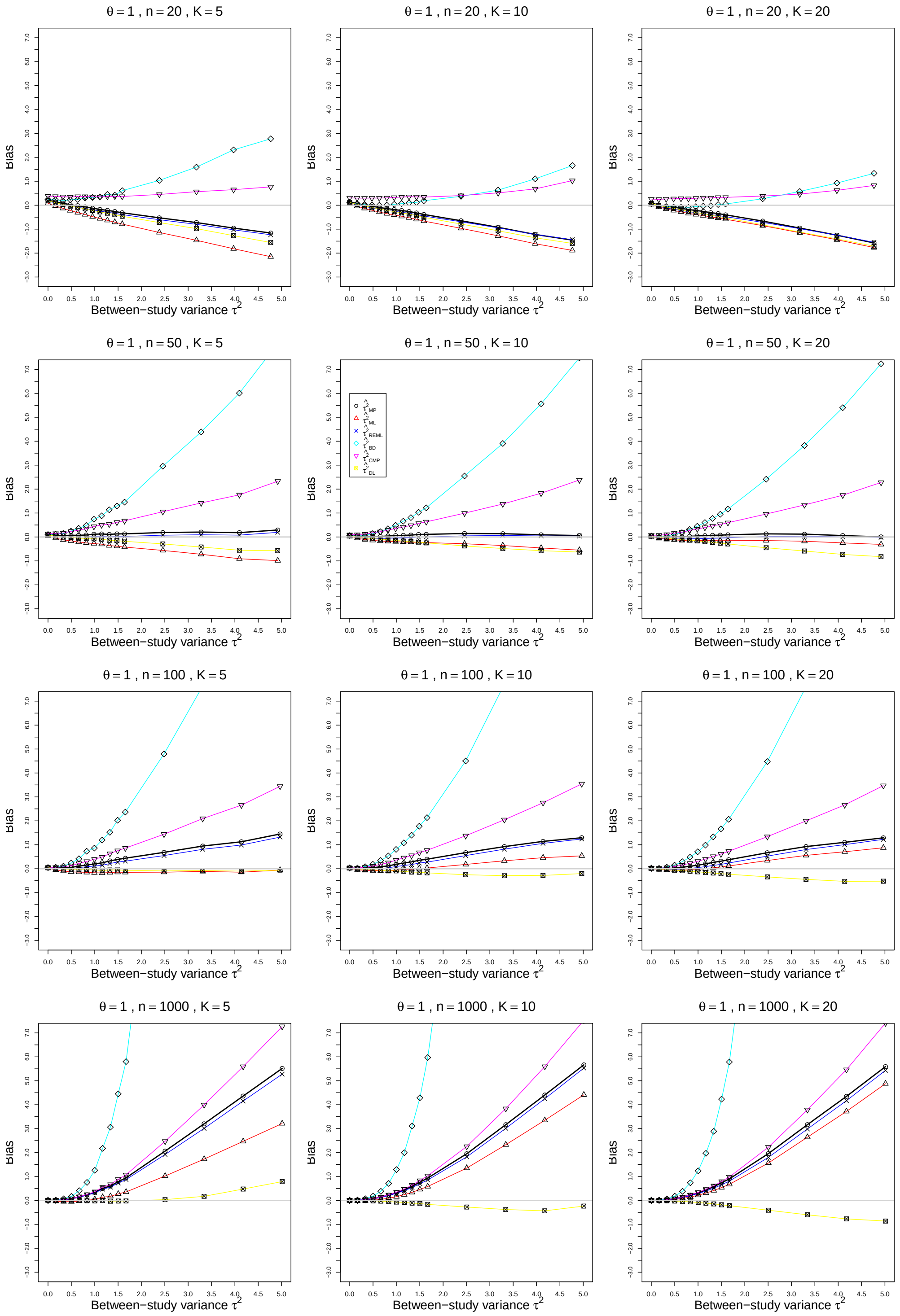

Figure D37. Bias estimated from K studies of the between study variance τ^2 in the beta-binomial model for $p_{2j} = 0.1$, $\theta = 1$ and $0 \leq \tau^2 \leq 5$ (equivalently $0 \leq \rho \leq 0.3$) for constant sample sizes $n = 20,\ 50,\ 100$ and 1000. Estimation methods: circles – Mandel-Paule estimator $\hat{\tau}^2_{MP}$, triangles – $\hat{\tau}^2_{ML}$, filled squares – Moment estimator $\hat{\tau}^2_{DL}$, crosses – $\hat{\tau}^2_{REML}$, diamonds- Breslow-Day based estimator $\hat{\tau}^2_{BD}$, reverse-triangles – Corrected Mandel-Paule estimator $\hat{\tau}^2_{cMP}$. Light grey line at 0.

Copyright © 2016 John Wiley & Sons, Ltd.

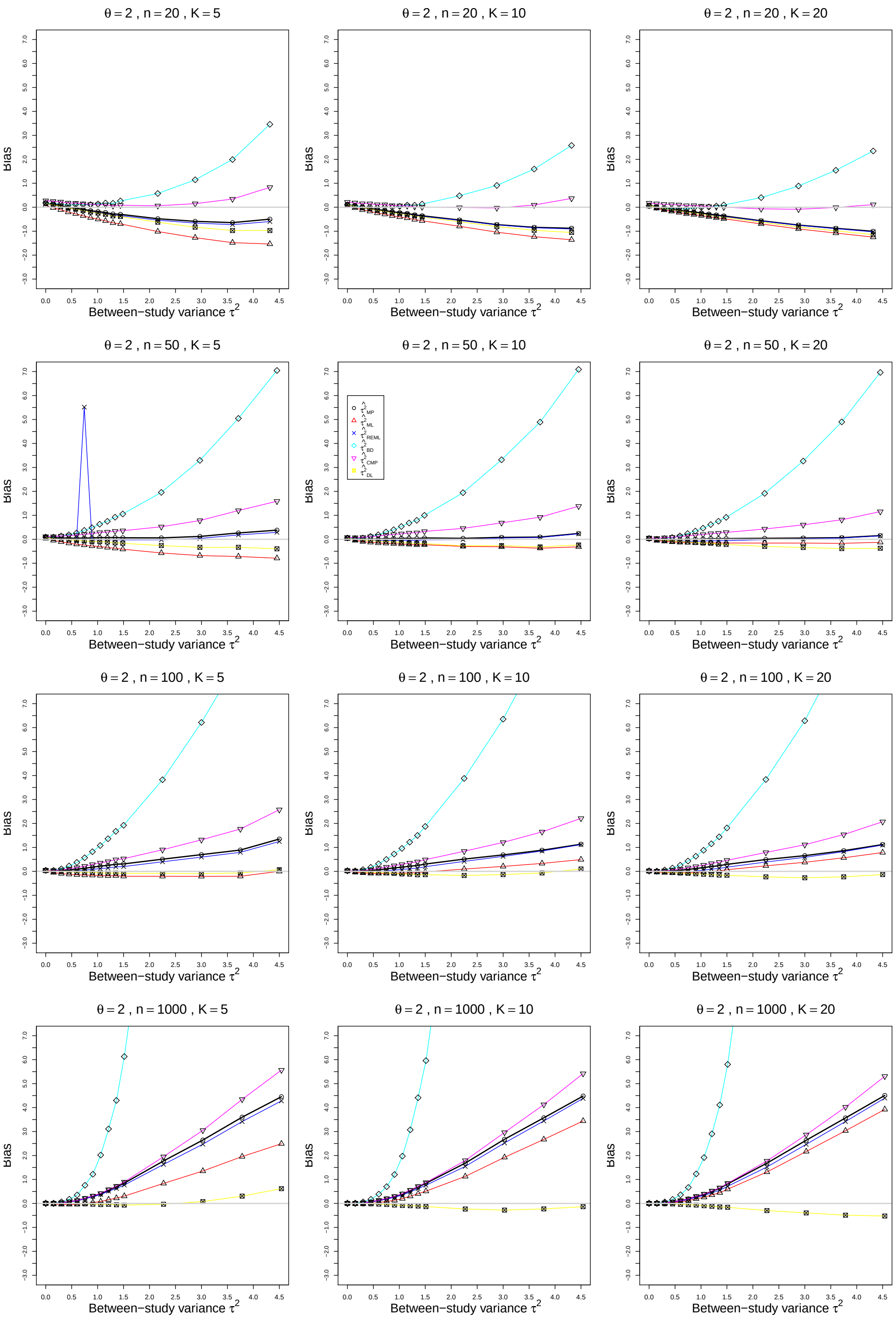

Figure D38. Bias estimated from K studies of the between study variance τ^2 in the beta-binomial model for $p_{2j} = 0.1, \theta = 2$ and $0 \leq \tau^2 \leq 4.5$ (equivalently $0 \leq \rho \leq 0.3$) for constant sample sizes $n = 20,\ 50,\ 100$ and 1000. Estimation methods: circles – Mandel-Paule estimator $\hat{\tau}^2_{MP}$, triangles – $\hat{\tau}^2_{ML}$, filled squares – Moment estimator $\hat{\tau}^2_{DL}$, crosses – $\hat{\tau}^2_{REML}$, diamonds- Breslow-Day based estimator $\hat{\tau}^2_{BD}$, reverse-triangles – Corrected Mandel-Paule estimator $\hat{\tau}^2_{cMP}$. Light grey line at 0.

Copyright © 2016 John Wiley & Sons, Ltd.

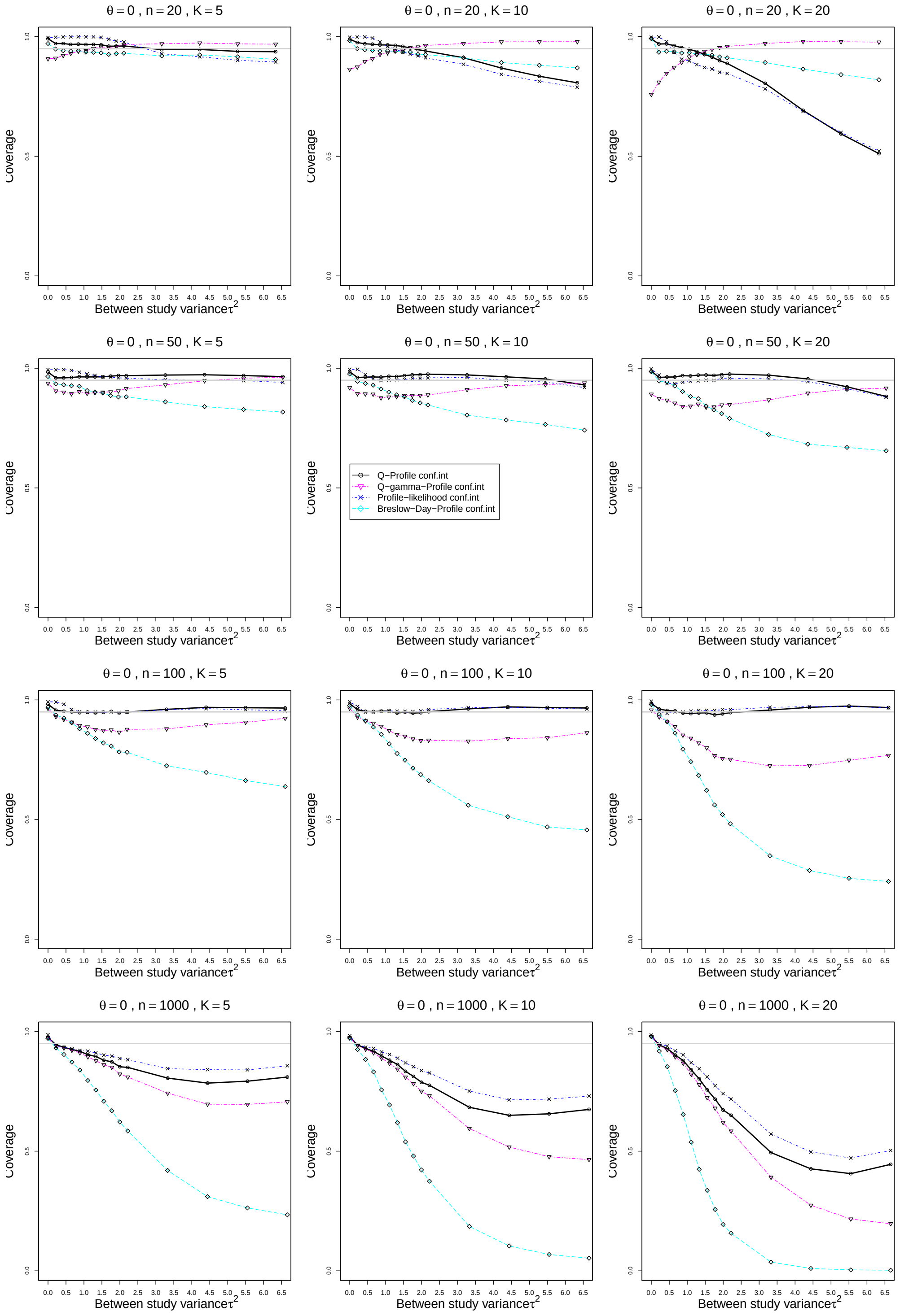

Figure D39. Coverage at the nominal confidence level of 0.95 of the between study variance τ^2 estimated from K studies in the beta-binomial model for $p_{2j} = 0.1$, $\theta = 0$ and $0 \leq \tau^2 \leq 6.5$ (equivalently $0 \leq \rho \leq 0.3$) for constant sample sizes $n = 20,\ 50,\ 100$ and 1000. Interval estimation methods: circles – Q-profile confidence interval for τ^2 based on χ^2 distribution, reverse triangles – Q-profile confidence interval for τ^2 based on $\Gamma_{r(\tau^2),\lambda(\tau^2)}$ distribution), crosses – Profile likelihood confidence intervals, diamonds – Breslow-Day-Profile confidence intervals. Light grey line at 0.95.

Copyright © 2016 John Wiley & Sons, Ltd.

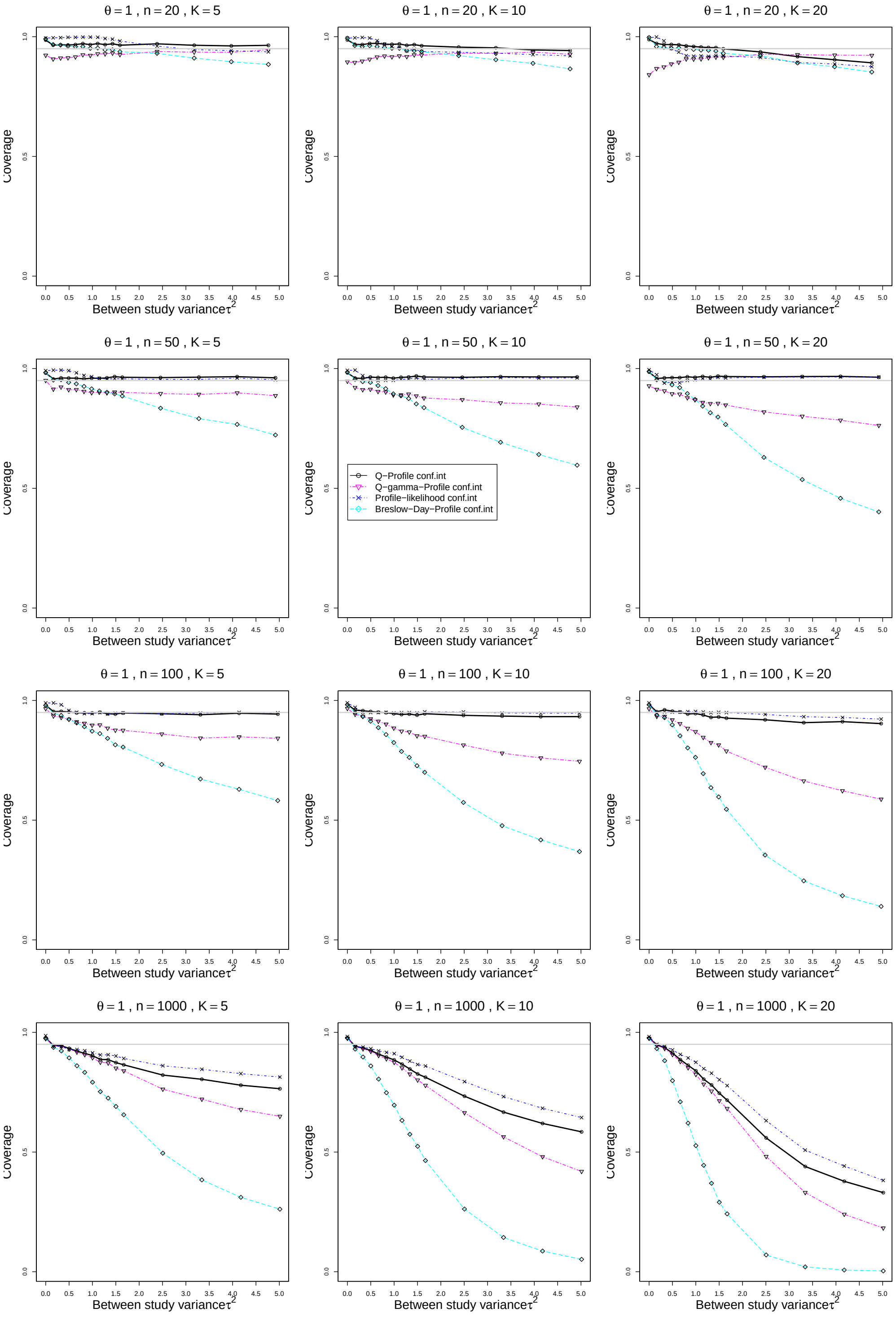

Figure D40. Coverage at the nominal confidence level of 0.95 of the between study variance τ^2 estimated from K studies in the beta-binomial model for $p_{2j} = 0.1$, $\theta = 1$ and $0 \leq \tau^2 \leq 5$ (equivalently $0 \leq \rho \leq 0.3$) for constant sample sizes $n = 20,\ 50,\ 100$ and 1000. Interval estimation methods: circles – Q-profile confidence interval for τ^2 based on χ^2 distribution, reverse triangles – Q-profile confidence interval for τ^2 based on $\Gamma_{r(\tau^2),\lambda(\tau^2)}$ distribution), crosses – Profile likelihood confidence intervals, diamonds – Breslow-Day-Profile confidence intervals. Light grey line at 0.95.

Copyright © 2016 John Wiley & Sons, Ltd.

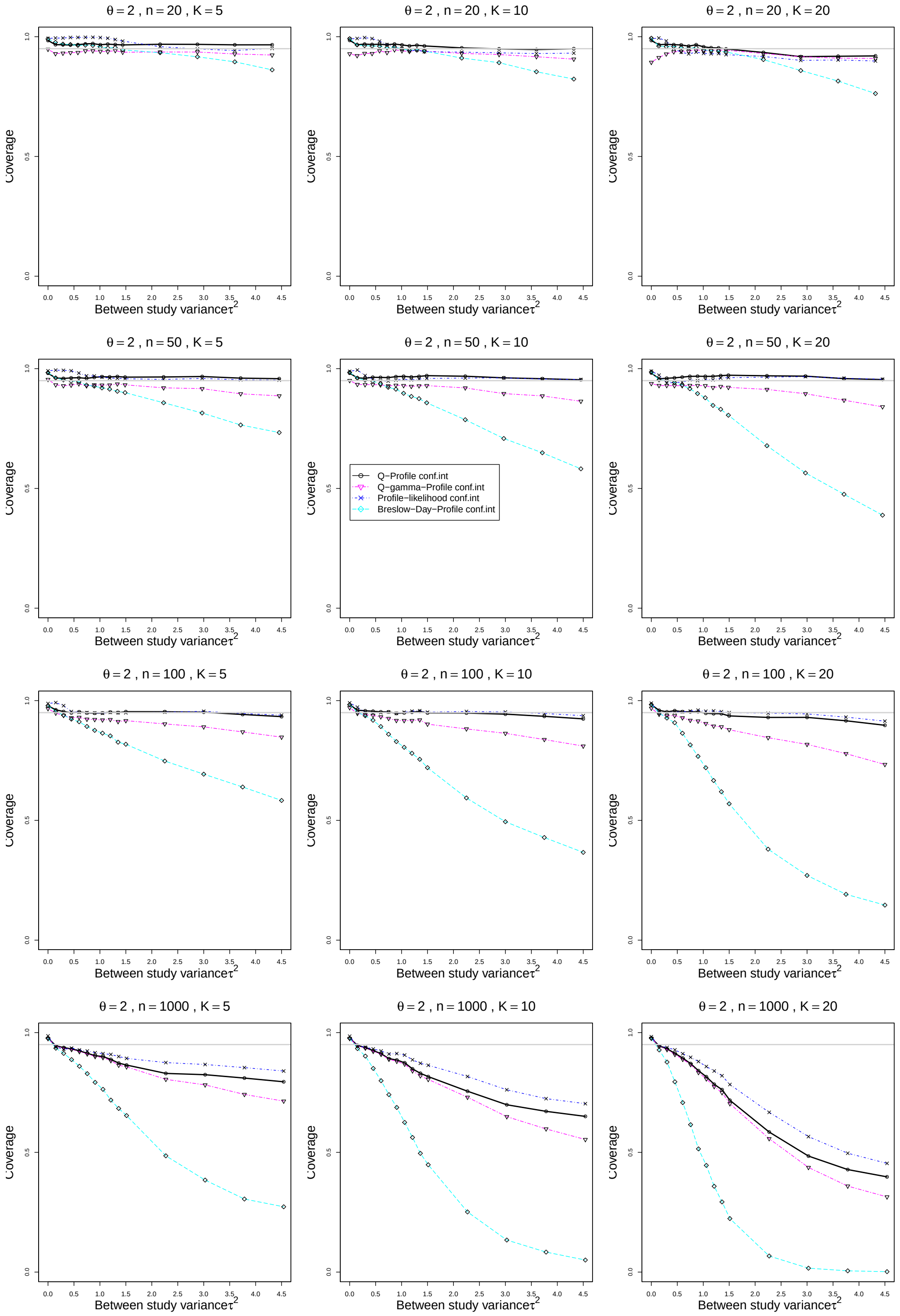

Figure D41. Coverage at the nominal confidence level of 0.95 of the between study variance τ^2 estimated from K studies in the beta-binomial model for $p_{2j} = 0.1$, $\theta = 2$ and $0 \leq \tau^2 \leq 4.5$ (equivalently $0 \leq \rho \leq 0.3$) for constant sample sizes $n = 20,\ 50,\ 100$ and 1000. Interval estimation methods: circles – Q-profile confidence interval for τ^2 based on χ^2 distribution, reverse triangles – Q-profile confidence interval for τ^2 based on $\Gamma_{r(\tau^2),\lambda(\tau^2)}$ distribution), crosses – Profile likelihood confidence intervals, diamonds – Breslow-Day-Profile confidence intervals. Light grey line at 0.95.

Copyright © 2016 John Wiley & Sons, Ltd.

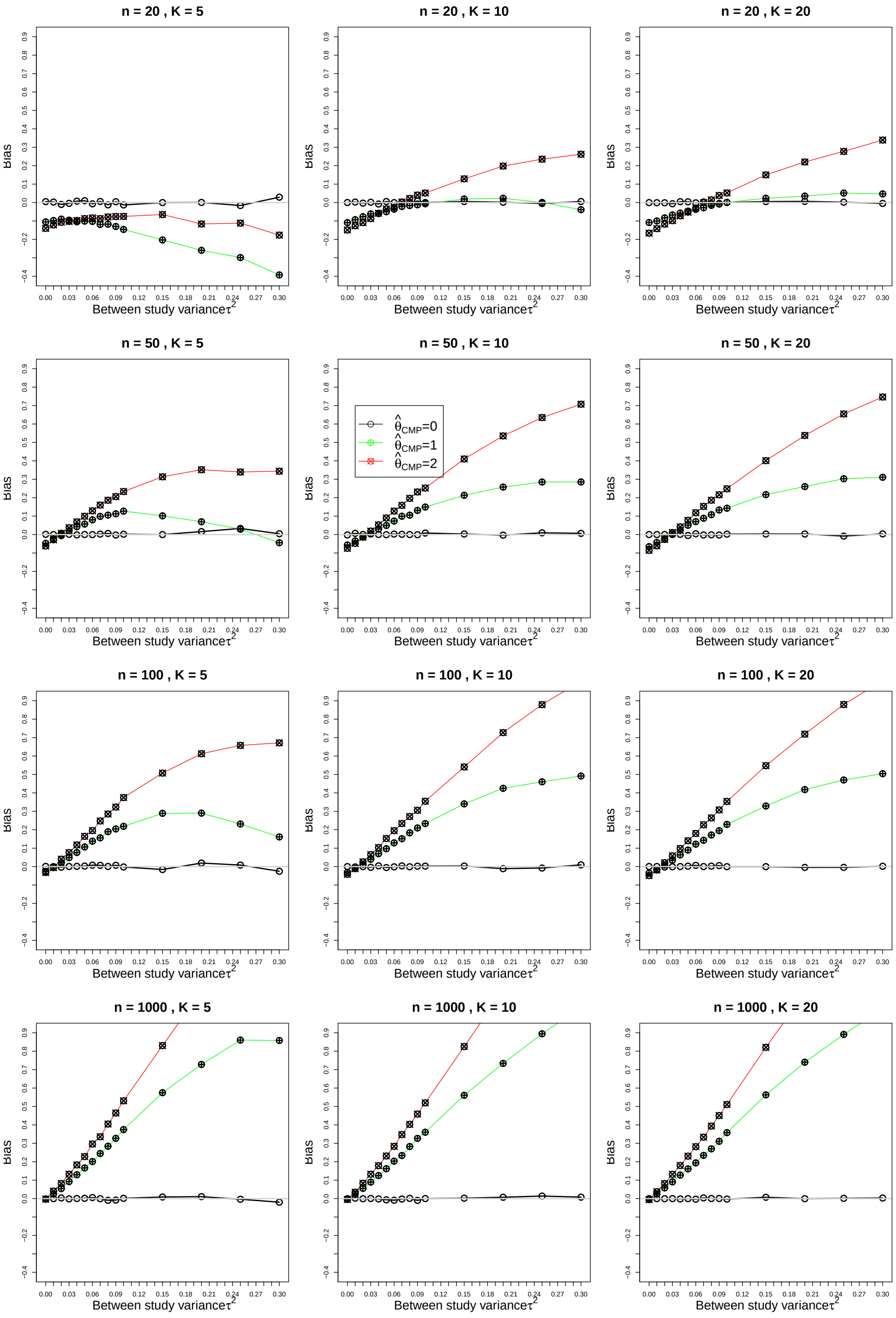

Figure D42. Bias of overall odds ratio $\hat{\psi}_{IV}$ obtained from K studies by the inverse-variance method with the moment estimator $\hat{\tau}^2_{cMP}$ in the weights, for $p_{2j} = 0.1$, and $0 \leq \rho \leq 0.3$ for constant sample sizes $n = 20,\ 50,\ 100$ and 1000. The biases are given for $\theta = 0$ (circles), $\theta = 1$ (circle plus), and $\theta = 2$ (circle cross). Light grey line at 0.

D.6. Bias and coverage of odds ratio

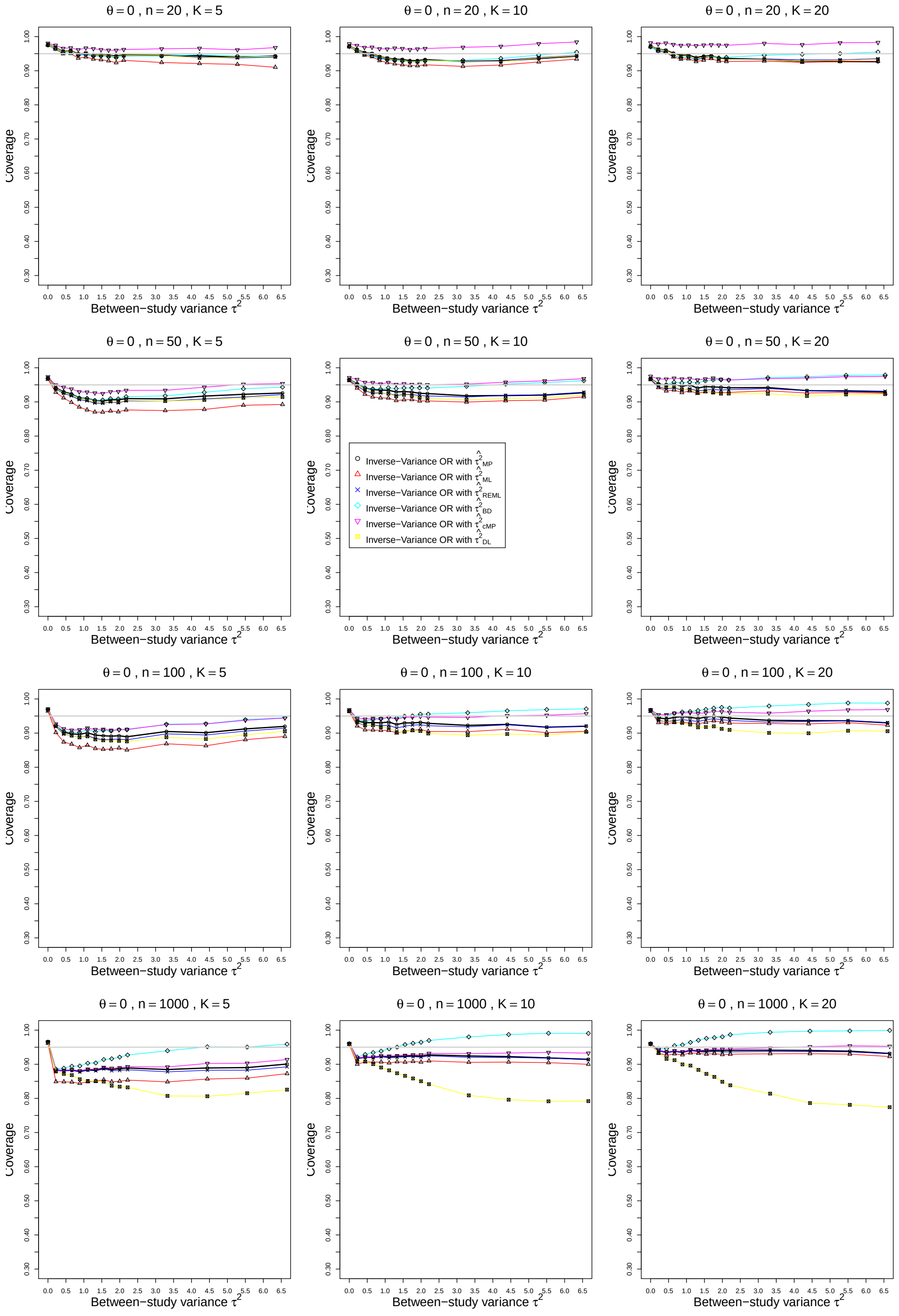

Figure D43. Coverage at the nominal confidence level of 0.95 of the overall odds ratio ψ obtained from K studies by the inverse-variance method, for $p_{2j} = 0.1$, $\theta = 0$ and $0 \leq \tau^2 \leq 6.5$ (equivalently $0 \leq \rho \leq 0.3$) for constant sample sizes $n = 20,\ 50,\ 100$ and 1000. The inverse-variance weights use the following estimators of τ^2: circles – Mandel-Paule estimator $\hat{\tau}^2_{MP}$, triangles – $\hat{\tau}^2_{ML}$, filled squares – Moment estimator $\hat{\tau}^2_{DL}$, crosses – $\hat{\tau}^2_{REML}$, diamonds- Breslow-Day based estimator $\hat{\tau}^2_{BD}$, reverse-triangles – Corrected Mandel-Paule estimator $\hat{\tau}^2_{cMP}$. Light grey line at 0.95.

Copyright © 2016 John Wiley & Sons, Ltd.

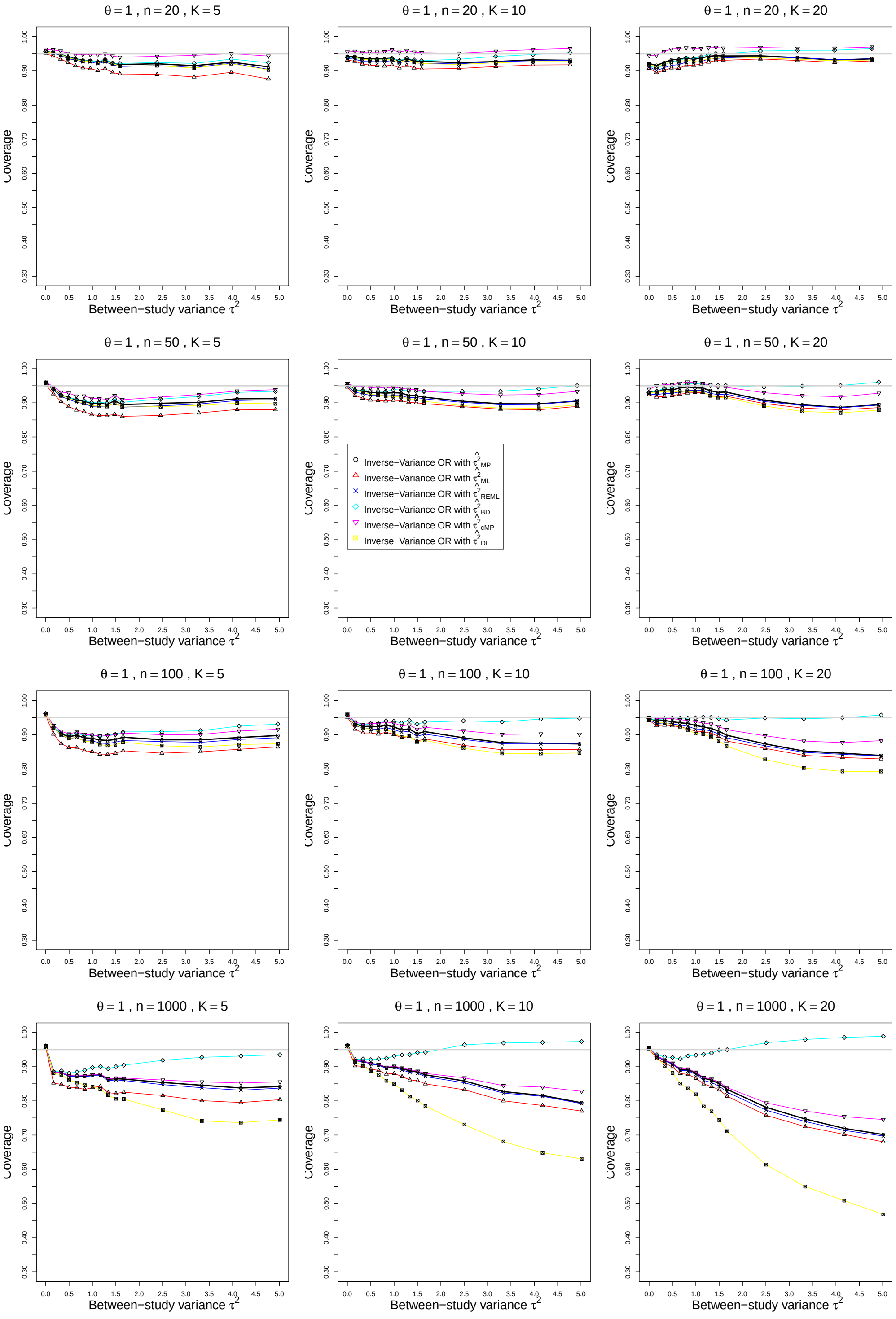

Figure D44. Coverage at the nominal confidence level of 0.95 of the overall odds ratio ψ obtained from K studies by the inverse-variance method, for $p_{2j} = 0.1$, $\theta = 1$ and $0 \leq \tau^2 \leq 5$ (equivalently $0 \leq \rho \leq 0.3$) for constant sample sizes $n = 20,\ 50,\ 100$ and 1000. The inverse-variance weights use the following estimators of τ^2: circles – Mandel-Paule estimator $\hat{\tau}^2_{MP}$, triangles – $\hat{\tau}^2_{ML}$, filled squares – Moment estimator $\hat{\tau}^2_{DL}$, crosses – $\hat{\tau}^2_{REML}$, diamonds- Breslow-Day based estimator $\hat{\tau}^2_{BD}$, reverse-triangles – Corrected Mandel-Paule estimator $\hat{\tau}^2_{cMP}$. Light grey line at 0.95.

 Copyright © 2016 John Wiley & Sons, Ltd.

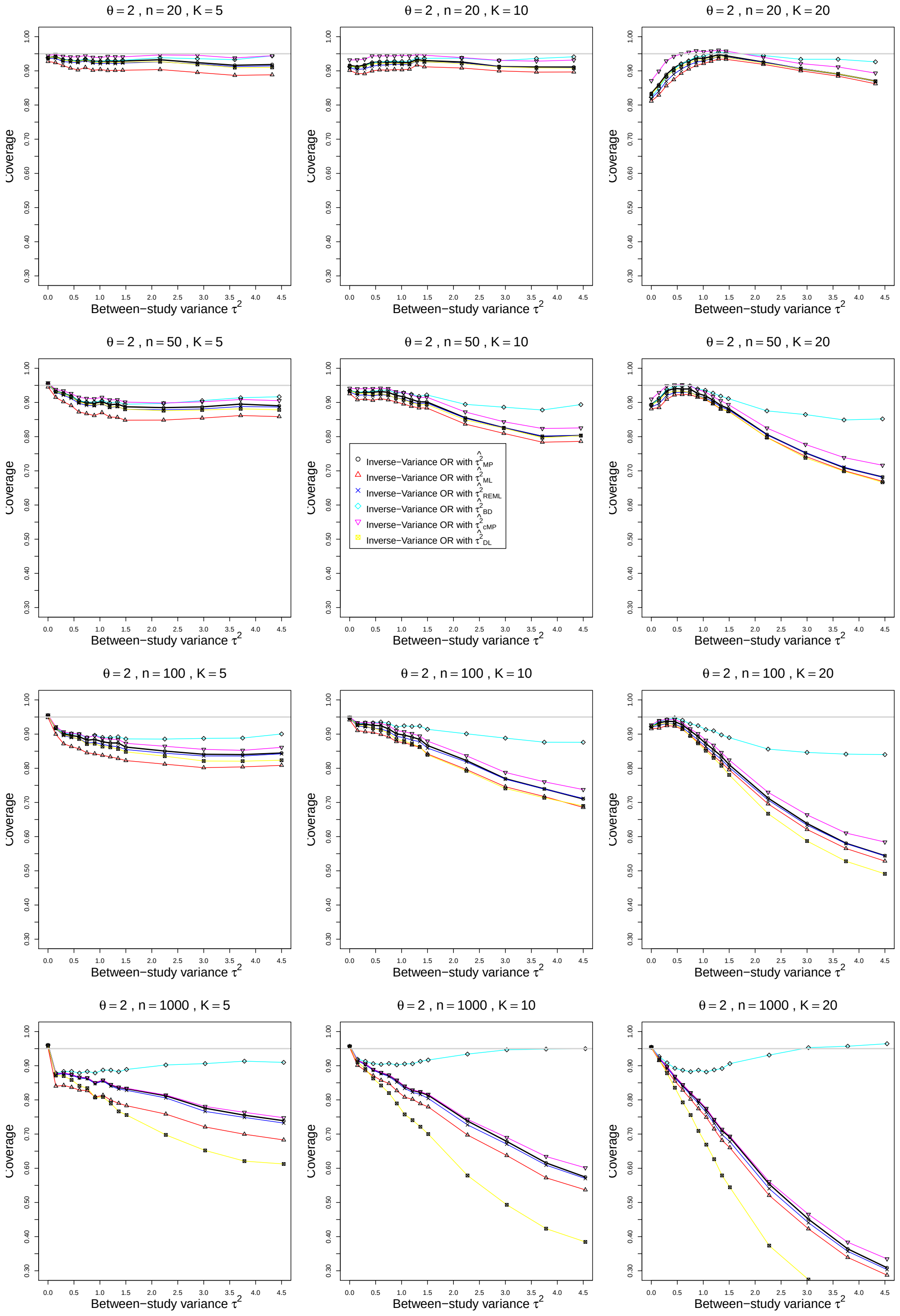

Figure D45. Coverage at the nominal confidence level of 0.95 of the overall odds ratio ψ obtained from K studies by the inverse-variance method, for $p_{2j} = 0.1$, $\theta = 2$ and $0 \leq \tau^2 \leq 4.5$ (equivalently $0 \leq \rho \leq 0.3$) for constant sample sizes $n = 20,\ 50,\ 100$ and 1000. The inverse-variance weights use the following estimators of τ^2: circles – Mandel-Paule estimator $\hat{\tau}^2_{MP}$, triangles – $\hat{\tau}^2_{ML}$, filled squares – Moment estimator $\hat{\tau}^2_{DL}$, crosses – $\hat{\tau}^2_{REML}$, diamonds- Breslow-Day based estimator $\hat{\tau}^2_{BD}$, reverse-triangles – Corrected Mandel-Paule estimator $\hat{\tau}^2_{cMP}$. Light grey line at 0.95.

Copyright © 2016 John Wiley & Sons, Ltd.
